# Leveraging Next‐Generation Tools for Genetic Assessment and Demographic Monitoring in Threatened and Elusive Humboldt Martens

**DOI:** 10.1111/eva.70277

**Published:** 2026-07-09

**Authors:** Margaret A. Hallerud, Katie Moriarty, Kristine L. Pilgrim, Cate B. Quinn, Charlotte E. Eriksson, Michael K. Schwartz, Taal Levi

**Affiliations:** ^1^ Department of Fisheries, Wildlife and Conservation Sciences Oregon State University Corvallis Oregon USA; ^2^ National Council for Air and Stream Improvement, Inc. Corvallis Oregon USA; ^3^ USDA Forest Service, Rocky Mountain Research Station National Genomics Center for Wildlife and Fish Conservation Missoula Montana USA

**Keywords:** fecal DNA, GT‐seq panel, imperiled species, noninvasive genetics, small carnivore, SNP panel

## Abstract

Conservation assessments of imperiled species require information on population structure, abundance, and connectivity which benefit from integrated demographic and genetic data. Humboldt martens (*Martes caurina humboldtensis*) are a federally threatened subspecies of Pacific marten ( *Martes caurina* ) whose conservation has been hindered by knowledge gaps and data deficiencies due to rarity and elusiveness. Here, we assessed population structure and genetic viability of Humboldt martens by exploring synergies between reduced representation sequencing and noninvasive genetic approaches. We used ddRADseq to discover 14,652 SNPs from 63 Pacific martens, then used these data to develop and validate a 94‐amplicon SNP panel for accurate individual and sex identification and broadscale genetic structure inference from Humboldt marten scats. Applying this SNP panel to 209 scats, two hair samples, and 24 low‐quality tissue samples, we identified 93 additional individuals and spatially expanded genetic structure inference relative to ddRADseq, including discovery of a previously undescribed population. We found genetic diversity was ~20% lower and inbreeding ~3 times higher on average in Humboldt martens relative to other Pacific marten populations, and Humboldt martens showed lower mitogenome nucleotide diversity than other genetically depauperate carnivore species and populations. Our combined analyses support genetic distinctness of Humboldt martens with limited evidence for hybridization with Oregon Cascades martens. Our data suggest geographic structuring of genetic viability within Humboldt martens, with strong signatures of isolation and small population size in the northernmost populations. Our approach demonstrates advantages and limitations of combining small genomic datasets and targeted SNP panels for conservation assessment and monitoring in fragmented and genetically depauperate species, and we call for expanded genetic viability assessment and monitoring in Humboldt martens.

## 1 Introduction

Assessing population structure, abundance, and viability of imperiled species is critical to effectively prioritizing conservation actions but is often hindered by data deficiencies. Conservation genetics provides an increasingly powerful toolbox (Hohenlohe et al. 2021; Forester and Lama 2023) that enables conservation assessment of rare and cryptic species where biological and logistical constraints otherwise limit direct inference on abundance, demography, and movement (Martin et al. 2022). Genetic variation is tightly linked with demographic and metapopulation processes (Frankham 1996; Baguette et al. 2013), and can therefore provide rapid insight into effective population size and connectivity. Further, genetic factors directly affect viability in small and isolated populations (Hedrick et al. 1996; Frankham 2005): small populations lose genetic diversity and accumulate harmful mutations through strong genetic drift and inbreeding, which consequently threatens long‐term adaptive capacity and population persistence (Keller and Waller 2002; Frankham 2005; Hoffmann et al. 2017; Willi et al. 2022). Connectivity can counteract these processes and improve persistence of small populations (Hanski 1991; Baguette et al. 2013), but immigration can be insufficient to overcome severe inbreeding (Kardos et al. 2017, 2025) and effects of previous genetic bottlenecks may have long‐term fitness consequences (e.g., Schmidt‐Küntzel et al. 2018). Population viability assessment of imperiled species therefore benefits from complementary genetic and demographic information (Clarke and Young 2000; Lowe and Allendorf 2010).

### 1.1 Next‐Generation Methods in Conservation Genetics: Challenges & Tradeoffs

Rapid cost declines for next‐generation DNA sequencing technologies have increased accessibility of genetic data, most notably via genomics which provides an increasingly powerful toolkit for studying non‐model species (Allendorf et al. 2010; Andrews et al. 2016; Hohenlohe et al. 2021). Genomics generates data on tens of thousands of genome‐wide markers, generally from known individuals, which can provide precise estimation of genetic structure (Willing et al. 2012), inbreeding (e.g., Robinson et al. 2016), genetic diversity, and evolutionary history (e.g., Colella et al. 2021). However, genomics requires high‐quality samples such as tissue or blood (but see Andrews et al. 2018) which are challenging to collect from imperiled species given the costs, potential regulations, and risks of live‐capturing animals. Noninvasive genetic sampling using lower‐quality DNA from hair, feathers, skin sheds, feces, and eDNA (e.g., snow tracks, water, air samples) is therefore more tractable for broad‐scale demographic and genetic monitoring of rare, elusive, and endangered species (Carroll et al. 2018). Combining deep but sample‐limited genomic information with shallow but sample‐abundant noninvasive genetic information therefore provides a promising approach for genetic inference in rare and understudied taxa. Genomics datasets also enable the development of objective‐focused targeted genotyping panels via identification of informative markers and validation of genetic inference (Hohenlohe et al. 2021).

Fecal DNA (hereafter, scat) samples are an increasingly utilized resource for diet analysis and noninvasive monitoring in wildlife, including expanding application of scats for estimating density, demographics, and genetic structure patterns (deOliviera et al. 2025). Extracting individual‐level genetic information from low‐quality samples requires targeted genotyping panels (e.g., Ekblom et al. 2021; Parker et al. 2021; Solari et al. 2025) that amplify informative loci specific to objectives (Waits and Paetkau 2005; Carroll et al. 2018). Genotyping scats is particularly challenging due to low concentrations of degraded DNA from target species and prevalence of non‐target DNA, primarily from bacteria but also from diet items and field contamination by other species (e.g., overmarking in carnivores; DeMatteo et al. 2018). Although genotyping of noninvasive genetic samples traditionally uses microsatellites (e.g., Schwartz et al. 2020; Day et al. 2022), existing microsatellite panels sometimes have low amplification success when applied to scats (e.g., Ruiz‐González et al. 2013; but see Schwartz et al. 2007; Dyck et al. 2024) and microsatellite results can be challenging to transfer among labs due to subjective allele calling. These limitations have motivated development of single nucleotide polymorphism (SNP)‐based panels that, when constructed carefully, have higher amplification success in degraded samples and facilitate reproducibility among labs. Successful methods for SNP‐based genotyping of scats include targeted sequencing using PCR primers (e.g., Eriksson et al. 2020) or hybridization probes (e.g., Parker et al. 2021), microfluidic arrays (e.g., Von Thaden et al. 2020), and mass spectrometry (e.g., Thavornkanlapachai et al. 2024). Of these, PCR‐based methods that genotype by amplicon sequencing (GBAS) are the most accessible option due to lower up‐front costs, use of standard laboratory equipment, and flexibility in running different numbers of loci and samples. Genotyping‐in‐thousands by sequencing (GT‐seq, Campbell et al. 2015) is an efficient GBAS protocol for SNP‐based genotyping of tissues using next‐generation sequencing, from which Eriksson et al. (2020) optimized methods for scat samples.

Designing reduced SNP panels for effective demographic and genetic monitoring is particularly challenging in genetically depauperate and structured populations. First, limited genetic diversity and high inbreeding increases the likelihood of multiple individuals sharing a genotype (Tokarska et al. 2009), but inaccurate individual identification also has greater consequences for inference in small populations (Waits and Leberg 2000). Second, under strong population structure, overall allele frequencies are not reflective of genotype frequencies due to the Wahlund effect, where allele fixation occurs at random in each subpopulation due to strong genetic drift (Garnier‐Géré and Chikhi 2013). Failing to account for genetic structure in candidate SNP selection consequently risks including SNPs that are monomorphic, and therefore uninformative for individual identification, within distinct populations. To avoid uninformative markers, candidate SNPs can be selected based on population‐level rather than overall allele frequencies, but this results in fewer candidates for a given minor allele frequency threshold. Lowering MAF thresholds to increase the number of candidate SNPs provides a solution and tradeoff: per‐SNP information content for individual identification is reduced, but power to detect genetic structure via modest variation in allele frequencies across populations is improved. Third, SNP panels are susceptible to ascertainment bias, the distortion of genetic inference due to unrepresentative sampling of allele frequencies during the SNP discovery process, which is exacerbated under strong population structure (Clark et al. 2005; Lachance and Tishkoff 2013). These three considerations apply to other marker types (e.g., microsatellite panels; Tokarska et al. 2009), but SNP panels are particularly susceptible due to lower levels of polymorphism and slower mutation rates. Genomics can help overcome these challenges by producing thousands of candidate markers.

### 1.2 Humboldt Martens as a Case Study

Noninvasive methods have played a key role in forest carnivore research given that alternative approaches are hindered by patchily distributed populations, low population densities, and elusive behavior (Long et al. 2008). Martens are small forest‐associated mammalian carnivores and hold conservation relevance as forest specialists that have been impacted by landscape change and predator control in recent centuries (Kurcera et al. 1996; Zielinski et al. 2001). Martens in North America include two morphologically and genetically distinct species: American martens ( *Martes americana* ) occur throughout eastern and northern portions of the continent, and Pacific martens ( *Martes caurina* ) occur in the west from southwestern British Columbia to the southern Sierra Nevada and eastwards into the Rocky Mountains (Carr and Hicks 1997; Small et al. 2002; Dawson et al. 2017; Colella et al. 2018). Hybridization between American and Pacific martens occurs along a narrow hybrid zone in the northern Rocky Mountains of Montana and on islands in southeast Alaska where American martens were introduced for fur trade (Colella et al. 2018). Evidence suggests asymmetric gene flow from American martens into Pacific martens (Lucid et al. 2020) and American martens outcompeting Pacific martens where they co‐occur (Small et al. 2002; Colella et al. 2019, 2024). Pacific martens are characterized by a patchy distribution reflecting a complex evolutionary history of colonization, isolation, and secondary contact in Pleistocene era glacial refugia (Small et al. 2002; Schwartz et al. 2020; Colella et al. 2021) and recent fragmentation and local extirpation driven by anthropogenic effects in the 1800s–1900s (Zielinski et al. 2001, 2005).

**FIGURE 1:**
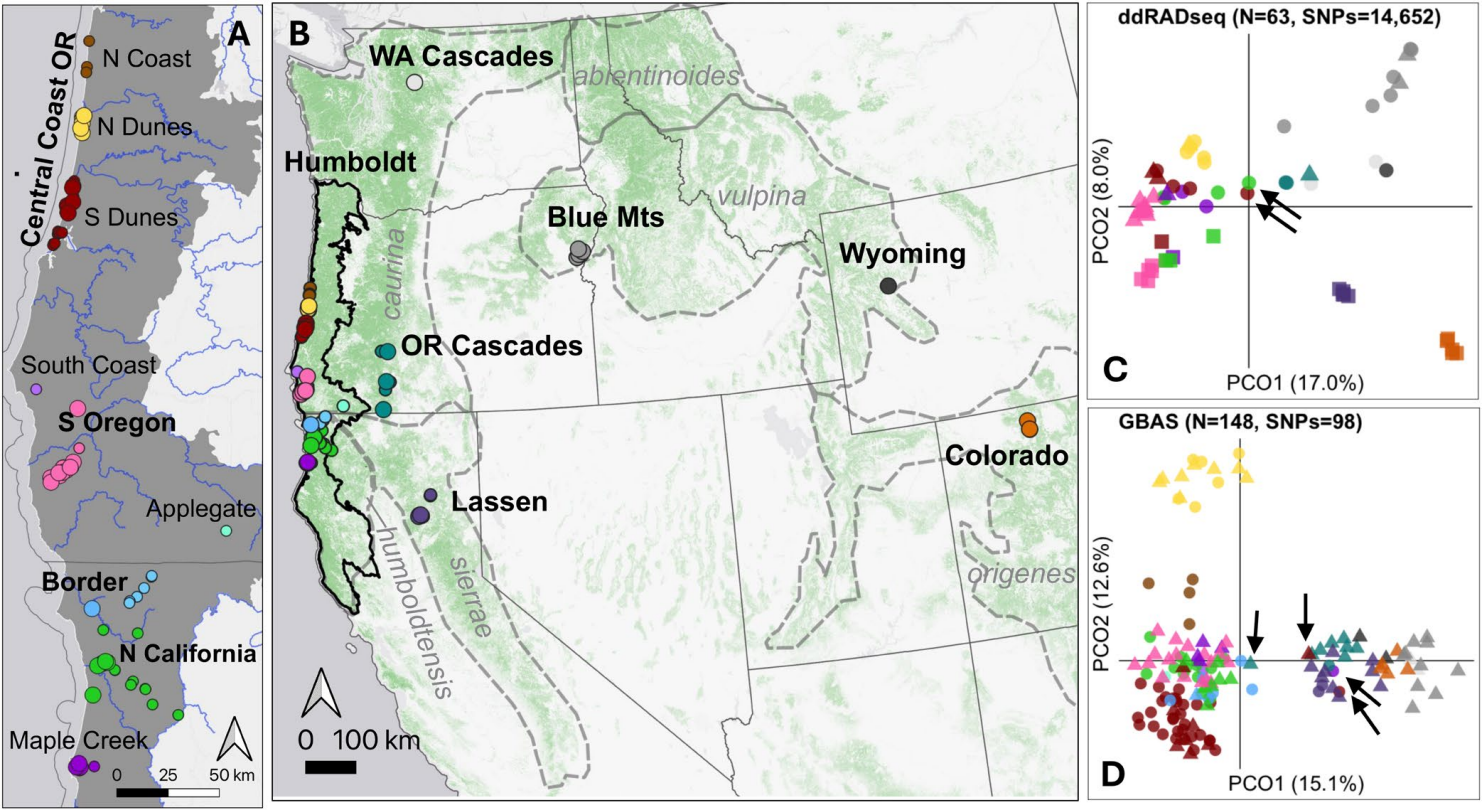
Maps of sampling effort where each point represents an individual marten included in ddRADseq (large points) or genotyped only by GBAS (small points), with colors reflecting sampling regions. (A) Humboldt marten sampling effort overlaid on updated historical range (gray; details in Supporting Information S1; shapefile in Appendix S1), extant population areas (green with bold labels; USFWS 2023), and major rivers (blue lines, National Hydrography Dataset). (B) Rangewide Pacific marten sampling effort overlaid on historical subspecies boundaries (gray, Hall 1981), updated Humboldt marten historical range map (dark outline), and current forest cover (green, National Land Cover Database [Dewitz 2023]). PCoAs show genetic structure for (C) the high‐missingness ddRADseq dataset with shapes reflecting sequencing batch and (D) GBAS dataset with circles reflecting noninvasive samples and triangles reflecting tissue samples. Point colors match sampling locations and arrows identify putative hybrids between Oregon Cascades and Humboldt martens.

Humboldt martens (also known as “coastal” martens; *M. c. humboldtensis*) are a subspecies of Pacific marten endemic to the Coast Ranges of northwestern California and western Oregon and are listed under the U.S. Endangered Species Act as a threatened Distinct Population Segment. Humboldt martens are genetically distinct based on mitochondrial genomes and microsatellite analyses (Slauson et al. 2009; Schwartz et al. 2020) and have morphological differences including smaller body size than other Pacific martens (Hall 1981) and smaller throat patches compared to Sierra Nevada martens (Grinnell and Dixon 1926). Humboldt martens are confined to 6% of their original range in four small and potentially isolated extant population areas on the Central Coast Oregon, southern Oregon, Oregon‐California border, and northern California (Linnell et al. 2018; Moriarty et al. 2021; Figure 1A). Given unresolved subspecies designations (Schwartz et al. 2020), we use the generic term ‘montane marten’ to describe inland populations of Pacific martens belonging to other subspecies. The nearest montane martens to Humboldt martens occur in the southern Cascades Mountains in Oregon and California.

Major knowledge gaps and data deficiencies hinder Humboldt marten conservation efforts (Martin et al. 2022; USFWS 2023). Defining Humboldt marten habitat has presented challenges given variation in occupied forest types, limitations of presence‐nondetection data, and the possibility that abundances of prey and co‐occurring carnivores rather than forest structure drive Humboldt marten distribution (Eriksson et al. 2019; Moriarty et al. 2021; Anderson et al. 2025; B. Barry et al. in prep). Much of the historical range of Humboldt martens remains sparsely surveyed (Moriarty et al. 2021), and consequently population definitions, connectivity, and the origin and significance of marten detections outside of known population areas remain uncertain. Rare admixture has been detected between Humboldt and montane martens using microsatellites (Schwartz et al. 2020), but microsatellites tend to overestimate hybridization and these signatures need to be further investigated with higher‐resolution information such as genome‐wide SNP markers (Szatmári et al. 2021). Fewer than 500 Humboldt martens are thought to exist rangewide (USFWS 2023), but formal abundance estimates only exist for the Central Coast Oregon (*N* = 51–87; Linnell et al. 2018) and Northern California (*N* = 73–146; Anderson et al. 2025) extant population areas. The Central Coast Oregon extant population area is geographically isolated and divided by a major river with extremely low abundances on either side (*N* = 30–51 [North], *N* = 21–36 [South]), with population viability threatened by demographic and environmental stochasticity (Linnell et al. 2018). Scat detection dogs have played a major role in research and monitoring of Humboldt marten distribution (Moriarty et al. 2021) and diet (Eriksson et al. 2019; Martin et al. 2025), yet individual‐based genetic and demographic analyses have been hampered by the ineffectiveness of microsatellite panels on marten fecal DNA (Moriarty et al. 2018).

Here, we combined genomics, GBAS panel development, and scat‐based noninvasive genetics to provide baseline genetic information and expand monitoring capacity for Humboldt martens. We used reduced representation sequencing of Pacific marten tissue samples to explore: (1) genetic distinctness of Humboldt martens from other Pacific martens, (2) genetic diversity and inbreeding of Humboldt martens relative to other Pacific marten populations, and (3) population structure within Humboldt martens. Next, we used these genomic resources to develop and validate a 94‐locus GBAS panel for demographic and genetic monitoring of Humboldt martens capable of individual and sex identification, differentiating Humboldt and montane martens, and estimating broad genetic structure within Humboldt martens. We then applied this new panel to genotype marten scats collected during survey efforts prior to the availability of an effective fecal genotyping assay. Finally, we leveraged genomic and noninvasive genetic results to synthesize current knowledge on metapopulation structure and population viability in Humboldt martens, and highlight key areas of future investigation.

## 2 Materials and Methods

### 2.1 Sample Collection and DNA Extraction

We used three types of Pacific marten samples: (1) 91 high‐quality (i.e., tissue and blood) samples from known individuals (including many samples previously analyzed in Schwartz et al. 2020), (2) 49 fresh scat samples from known individuals, including 15 scats where identifying information was masked from lab staff for GBAS panel testing, and (3) 308 noninvasively collected samples from unknown individuals, most of which were collected during USFWS‐permitted scat detection dog surveys to evaluate carnivore distribution and diet (see details in Moriarty et al. 2021; Barry et al. 2021; Martin et al. 2025). Field‐collected noninvasive samples were genetically verified as Pacific marten using 12S metabarcoding (Eriksson et al. 2019; Moriarty et al. 2021). Sample details are in Supporting Information S2 and Table S1. We used high‐quality samples with sufficient DNA (> 200 ng) for genomic sequencing, fresh known scats and high‐quality samples with > 1 ng/μl DNA remaining after genomics for GBAS panel testing and validation, and noninvasive samples from unknown individuals for application of the GBAS panel.

### 2.2 Reduced Representation Sequencing and SNP Discovery

We submitted 80 high‐quality samples (50 Humboldt martens and 30 “montane” martens; Table S2) across three libraries to Oregon State University's Center for Quantitative Life Sciences (CQLS) for double‐digest RADseq (ddRADseq; Peterson et al. 2012; details in Supporting Information S3). We included 18 technical replicates, derived from 5 individual martens, within and across libraries to estimate genotyping accuracy and library effects (Bresadola et al. 2020).

We used the *de novo* SNP discovery pipeline in Stacks v2.65 (Catchen et al. 2013; Rochette and Catchen 2017) to call SNPs following parameter optimization (Mastretta‐Yanes et al. 2014; McCartney‐Melstad et al. 2019; Paris et al. 2017). Parameter optimization details are in Supporting Information S4 and results are shown in Figures S2–S4. We ran de novo rather than reference‐based SNP calling to avoid sequencing data loss caused by using a reference genome (i.e., *Martes flavigula* , Mei et al. 2023) from a different subgenus (Koepfli et al. 2008; Bohling 2020). We strictly filtered SNPs following recommendations in O'Leary et al. (2018): retaining SNPs with MAC ≥ 2, depth ≥ 5 (mean ≥ 8), and missingness < 30% and then removing samples with > 50% missing SNPs (details in Supporting Information S5 and Table S3). We further filtered this “high‐missingness” dataset to create a linkage disequilibrium pruned (hereafter, “LD‐pruned”) dataset and a “low‐missingness” dataset with missingness ≤ 5% for samples and ≤ 10% for SNPs. LD‐pruning used a sliding window approach where SNPs with > 0.60 correlation were removed. We calculated genotyping error via program Tiger (Bresadola et al. 2020) and estimated library effects using principal variance components analysis (PVCA) (Li et al. 2009) on the high‐missingness dataset in R. We used the LD‐pruned dataset in most analyses to ensure independent markers and the low‐missingness dataset when missingness would bias results (see Section 2.4). We used R version 4.2.1.

### 2.3 GBAS Panel Development and Application

#### 2.3.1 Candidate SNP Selection

Given that our primary goal for GBAS panel development was noninvasive demographic monitoring, we focused candidate SNP selection on reliable individual identification across Humboldt marten populations and did not explicitly select SNPs for secondary goals (e.g., population assignment, relatedness estimation). Using LD‐pruned ddRADseq data from the first two libraries, we identified candidate loci with the following criteria: We removed Y‐associated loci based on alignment to the *Mustela erminea* Y chromosome (GenBank: GCA_009829155.1) and loci with SNPs in the first 25 bp or last 20 bp which could interfere with primer binding. We then selected SNPs with a minor allele frequency (MAF) ≥ 0.10 within > 3 Humboldt marten populations to avoid allele fixation that would hinder individual identification while also allowing for variation in allele frequencies among populations which reflects population structure. We further filtered candidate loci by requiring unique alignment to the *Martes flavigula* genome (GenBank: GCA_029410595.1; Mei et al. 2023) and removing known repetitive elements within mammalian carnivores by searching against CENSOR repBase (Kohany et al. 2006).

#### 2.3.2 In Silico Optimization and Specificity Checks

Following specifications in Eriksson et al. (2020), we designed primer pairs for each candidate locus using Primer3 (Untergasser et al. 2012) and predicted dimers in multiplex primer sets using MFEprimer v3.2.7 (Wang et al. 2019). We designed an initial set of 150 multiplex PCR primer pairs via in silico optimization for the minimum cumulative dimer load (Xie et al. 2022). Next, we checked multiplex PCR primer pairs for specificity against Mustelidae and known prey species (e.g., mice *Peromyscus* spp., voles *Myodes* spp., chipmunks *Tamias* spp., “squirrels,” “shrews,” birds “Aves,” black‐tailed deer *Odocoileus hemionus* , plethodontid salamanders *Plethodon* spp.; Eriksson et al. 2019; Martin et al. 2025) using PRIMER‐BLAST (Ye et al. 2012). We ordered the final set of locus‐specific primers from Integrated DNA Technologies with a 5′ overhang Illumina adapter sequence.

#### 2.3.3 Lab Optimization of Multiplex PCR

We followed GBAS PCR reaction and library prep protocols described in Eriksson et al. (2020) with slight modifications, most notably using unique dual indexes. We ran three PCR replicates for noninvasive samples and two PCR replicates for high‐quality samples. We first tested the 150‐plex GBAS panel in equimolar concentrations (0.2 μM per primer) on two tissue samples and five scat samples from known martens. We assessed on‐target rates, on‐target read counts, and primer interactions using the GT‐seq pipeline (Campbell et al. 2015). Based on results from this initial multiplex test, we removed primer pairs that failed to amplify in scats, removed one primer pair for each primer dimer interaction that affected amplification success, and adjusted primer concentrations to better distribute sequencing depth across loci. At this step, we preferentially retained primer pairs with high information content (e.g., microhaplotypes, SNPs with large allele frequency differences between Humboldt and montane martens, and SNPs with higher MAF across Humboldt populations). We tested six primer pairs for sex identification in singleplex on known male and female samples, then added sex identification primer pairs to the optimized multiplex if they consistently amplified only in known males and had an amplicon size < 200 bp (see Supporting Information S6, Table S5).

#### 2.3.4 Testing Identification Accuracy, Error Rates, and Specificity

Next, we tested the lab‐optimized GBAS panel's ability to accurately sex and identify individuals by genotyping fresh scats and tissue from known livetrapped animals. We called genotypes for each PCR replicate from sequencing data using the GT‐seq pipeline (Campbell et al. 2015), formed consensus genotypes for each sample, and identified individuals using a clustering approach where samples with high levels of pairwise SNP matches were checked for concordance with other samples in the cluster (Thavornkanlapachai et al. 2024). For consensus genotyping, we required all replicates to be homozygous to call a homozygote and each allele to be present in at least two replicates to call a heterozygote. We assessed concordance of individual identification with known identities of samples.

We calculated GBAS panel genotyping error rates based on samples belonging to known individuals. We identified genotyping errors as allele dropouts (i.e., when a SNP call was homozygous but the reference genotype was heterozygous) or false alleles (i.e., when a SNP call was heterozygous but the reference genotype was homozygous) (Broquet and Petit 2004). We first calculated genotyping error rates per PCR replicate for each sample type (*N* = 80 tissue × 2 replicates and *N* = 45 scats × 3 replicates) using each sample's consensus genotype as the reference. We then calculated sample‐level and SNP‐level genotyping error rates per fresh scat sample (*N* = 45) using tissue‐based consensus genotypes from the same known individuals (*N* = 29) as the reference. Finally, to assess specificity, we ran the GBAS panel on tissue samples from three common prey species (deer mouse *Peromyscus maniculatus* , western red‐backed vole *Clethrionomys californicus* , and Townsend's chipmunk *Neotamias townsendii*) and four closely related or co‐occurring small carnivores (American marten *Martes americana* , fisher *Pekania pennanti* , gray fox *Urocyon cineoargenteus*, and mink *Neogale vison*).

#### 2.3.5 Application for Individual Identification

We applied the optimized GBAS panel to all high‐quality samples not consumed by ddRADseq (*N* = 84), including 18 individuals excluded from our ddRADseq results due to low DNA concentrations or high data missingness, and to all field‐collected noninvasive samples available (*N* = 308). We assigned sex and individual identity to each sample with ≥ 75 genotyped SNPs, requiring ≤ 4 mismatches (≤ 4 allele dropouts and ≤ 1 false allele) between samples attributed to the same individual based on observed consensus genotyping error rates (see Section 3.4). For each individual, we then formed a consensus genotype across samples. Individuals genotyped by both ddRADseq and GBAS formed the “validation” dataset while individuals genotyped from all sample types formed the “full” GBAS dataset.

Prior to further analyses, we checked for additional SNPs (i.e., microhaplotypes) using raw sequencing data for the full GBAS dataset. We formed reference amplicons by first identifying unique sequences with read depth > 200 in at least one individual and then clustering by 95% similarity using usearch v11 (Edgar et al. 2011). We aligned unique sequences to these clustered reference loci and called SNPs using bwa mem v0.7.19 (Li and Durbin 2009) and bcftools v1.21 (Danecek et al. 2021). We genotyped SNPs discovered at this stage across all samples and included these in further analyses.

#### 2.3.6 Quantifying Power and Ascertainment Bias

We used the full GBAS dataset to quantify power and check for ascertainment bias created by focusing panel design on Humboldt martens. To assess power for individual identification, we calculated the number of SNPs, mean individual heterozygosity, PID, PID_SIB_ (Waits et al. 2001), and PID_UB_ (Paetkau and Strobeck 1994) in each sampled region following formulas in Waits et al. (2001). We assessed GBAS panel power for secondary objectives such as assigning population of origin and resolving genetic structure. To assess power for population assignment, we calculated *f*_ORCA_ (i.e., the Optimal Rate of Correct Assignment; Rosenberg 2005) for each population with *N* ≥ 5 by simulating 1000 genotypes based on observed allele frequencies within the population, assigning simulated genotypes to a population based on the maximum probability of the genotype, and then summarizing the proportion of genotypes correctly assigned. Lastly, we calculated informativeness for assignment (*I*_n_) per locus which may perform better in cases of isolation‐by‐distance (Rosenberg et al. 2003).

### 2.4 Population Genetics

#### 2.4.1 Population Structure

We described genomic structure in Pacific martens using support from multiple approaches to counteract potential biases caused by small and unbalanced sample sizes (Puechmaille 2016; Lawson et al. 2018; Elhaik 2022). Sample sizes in our genomics dataset were small but met existing guidelines indicating that population structure can be reliably estimated with as few as 2–4 individuals (Willing et al. 2012) and heterozygosity with 6–8 individuals (Nazareno et al. 2017) when thousands of SNPs are genotyped, although exact sampling requirements will vary by system (Flesch et al. 2018). Our sample sizes likely capture > 10% of Humboldt marten populations (Linnell et al. 2018; USFWS 2023) except for Northern California where sampled individuals represent ~5% of the population (Anderson et al. 2025). We did not purge close relatives prior to analyses because high relatedness is a true signal of genetic structure in small populations and purging close relatives can further bias inference (Waples and Anderson 2017).

First, we used the high‐missingness dataset to visualize patterns of genetic variation via principal coordinates analysis (PCoA) calculated in R package adegenet v2.1.11 (Jombart 2008). Second, we used ADMIXTURE models (Alexander et al. 2009) to identify genetic clusters and estimate ancestry of each individual within (1) all Pacific martens and (2) Humboldt martens. For each dataset, we tested for *K* = 1–12 clusters and identified K values that best represented our data based on minimizing 10‐fold cross‐validation error and maximizing the increase in log‐likelihood, then assessed model fit by visualizing correlation structure in residuals using evalAdmix (Garcia‐Erill and Albrechtsen 2020). We tested whether ADMIXTURE clusters were biased by close relatives (Rodriguez‐Ramilo and Wang 2012) or unbalanced sampling (Lawson et al. 2018) by rerunning models after removing first‐order relatives based on the KING‐robust estimator (Manichaikul et al. 2010) and with one sample per locality. Third, we ran fineRADstructure (Malinsky et al. 2018), which uses linkage disequilibrium patterns to model hierarchical genetic structuring ranging from population structure to individual relatedness (Lawson et al. 2012). We ran fineRADstructure on the low‐missingness dataset because high levels of missingness skew results (Malinsky et al. 2018).

We calculated fixation indices (*F*_ST_; Weir and Cockerham 1984) to quantify partitioning of genetic variation within Pacific martens using the R package hierfstat v0.5.11 (Goudet and Jombart 2022). We quantified genetic differentiation between regions using pairwise Nei's *F*_ST_ (Nei 1987) and calculated confidence intervals with 999 bootstraps over loci. We calculated population‐specific *F*_ST_ (Weir and Goudet 2017) to quantify deviation from a common ancestral state and contributions to cumulative genetic diversity relative to all Pacific martens for Humboldt and montane groups, relative to all Humboldt martens for each Humboldt marten population, and relative to all montane martens for each montane marten population. Finally, we tested for rangewide isolation‐by‐distance in Pacific martens using a Mantel test (Mantel 1967) to check for correlation between pairwise genetic differentiation (*F*_ST_) and geographic distance among sampled regions using 999 permutations in the R package vegan (Oksanen et al. 2025).

#### 2.4.2 Introgression Patterns

Mixed ancestry in genetic structure results can arise from true hybridization (i.e., secondary contact between lineages) or isolation‐by‐distance (i.e., shared ancestry across a genetic cline). To distinguish these patterns, we constructed triangle plots with hybrid index (i.e., admixture proportion) on the x‐axis and interclass heterozygosity on the y‐axis using triangulaR (Wiens and Colella 2024). To identify ancestry‐informative markers, we tested allele frequency differences between populations (δ) of 0.7, 0.8, and 0.9 and proceeded with δ=0.7, which balanced information content per SNP (δ) with the number of SNPs meeting that threshold; results were consistent with δ=0.8 but δ=0.9 returned < 10 SNPs which reduced resolution.

#### 2.4.3 Phylogenomic Analysis

We constructed a phylogeny to identify patterns of molecular evolution using IQ‐Tree2 (Minh et al. 2020) on the low‐missingness ddRADseq dataset. First, we identified the best‐supported substitution model via ModelFinder with ascertainment bias correction (Kalyaanamoorthy et al. 2017) and constructed an unrooted maximum‐likelihood phylogeny using 1000 ultra‐fast bootstraps to quantify confidence in each split. Then, we inferred the root position using a generalizable non‐reversible DNA substitution model (Naser‐Khdour et al. 2021). We visualized results in FigTree v1.4.4 (Rambaut 2018).

#### 2.4.4 Genetic Diversity and Inbreeding

Given biases in population‐level estimates when sample sizes are small (Schmidt et al. 2021; Sopniewski and Catullo 2024), we focused on individual‐based and rarefied metrics of genetic diversity and inbreeding. We estimated individual and population‐level inbreeding coefficients ($F_{\beta }$) using the allele‐matching calculations described in Weir and Goudet (2017) and implemented in R package SNPRelate (Zheng et al. 2012). We estimated genetic diversity via population‐level allelic richness (AR, a rarefied metric of allelic diversity), population‐level allelic richness of private alleles (AP), individual‐based genome‐wide heterozygosity (*H*_A_; we did not exclude sex‐linked loci following Schmidt et al. 2021), and individual‐based SNP heterozygosity (*H*_SNP_). We also calculated standard population‐level metrics including observed heterozygosity (*H*_O_), expected heterozygosity (*H*_S_), and inbreeding coefficients (*F*_IS_) which we calculated as the mean of $F_{\beta }$ (Weir and Goudet 2017). Calculations used plink v1.7 (Purcell et al. 2007) and R packages hierfstat (Goudet and Jombart 2022) and PopGenReport (Adamack and Gruber 2014).

Using ddRADseq data, our goal was to understand *relative trends* of genetic diversity and inbreeding within Humboldt martens and montane martens in this study. RADseq, however, tends to underestimate genetic diversity and provides a poor benchmark for comparison across studies given variation introduced by differences in sequencing, SNP discovery, and filtering methods (Cariou et al. 2016; Schmidt et al. 2021; Sopniewski and Catullo 2024). To provide estimates of genetic diversity in Humboldt martens that may be compared directly to other species and populations, we calculated regional nucleotide diversity for Pacific martens from published full mitochondrial genome assemblies (Schwartz et al. 2020, GenBank: MT028185‐MT028278; Colella et al. 2021, GenBank: PRJNA626623).

### 2.5 GBAS Panel Validation for Population Genetic Inference

We assessed concordance of population genetics inference between ddRADseq and GBAS via one‐to‐one comparisons using samples genotyped by both methods (*N* = 59). We compared the first two PCoA axes, pairwise fixation index (*F*_ST_) between populations, individual relatedness (𝛽_jj_, Weir and Goudet 2017), individual heterozygosity estimates (*H*_SNP_), and individual inbreeding coefficients (*F*_β_) using Pearson's correlations. We calculated PCoAs, *F*_ST_, *H*_SNP_, and *F*_β_ following methods described in Section 2.4. We estimated relatedness using the 𝛽_jj_ estimator (Weir and Goudet 2017), which is effective at estimating kinship from SNPs even in small populations (Goudet et al. 2018).

We also estimated contemporary effective population sizes (*N*_e_) based on the full ddRADseq and GBAS datasets. We estimated *N*_e_ for each sample‐year combination using the LDNE method implemented in NeEstimator v2.1 (Do et al. 2014) for the GBAS dataset and CurrentNe (Santiago et al. 2024) for the low missingness LD‐pruned ddRADseq dataset. CurrentNe provides more precise confidence intervals and consistent, accurate estimates even with small sample sizes (Santiago et al. 2024), but was not used for GBAS data because the model relies on genome‐scale resolution to identify close kin.

### 2.6 Expanding Genetic Structure Inference With GBAS

We used the full GBAS dataset (including genotypes from all sample types) to bolster sample sizes and geographically expand genetic structure information relative to ddRADseq analyses. We reran PCoA for all Pacific martens and just Humboldt martens to visualize patterns in genetic similarity, and we recalculated *F*_ST_ with a focus on characterizing genetic distance between populations and individuals found outside of previously described populations. To further explore the GBAS panel's ability to resolve genetic structure within Humboldt martens, we built a maximum‐likelihood tree based on Prevosti's distance with two American marten genotypes as the outgroup using the R package phangorn (Schliep 2011).

## 3 Results

### 3.1 Reduced Representation Sequencing Dataset

Our pre‐filtered ddRADseq dataset returned 516,545 SNPs across 73 individual Pacific martens with an effective per‐sample mean coverage of 15.8× (SD = 8.3×). The high‐missingness dataset included 14,652 SNPs and 63 martens, the LD‐pruned dataset 12,389 SNPs and 63 martens, and the low‐missingness dataset 3586 SNPs and 58 martens (Table S3). Our mean genotyping error rate across SNPs in the high‐missingness dataset was 0.8% with higher error rates in heterozygous sites (4.8%), which reflects allele dropout and was comparable with published benchmarks for RADseq (Bresadola et al. 2020). Batch effects explained 13% of variation (plate 1: 0.3%, plate 2: 5.3%, plate 3: 7.4%; Figure S7) and were likely associated with batch‐level variation in sequencing depth (Figures S5−S7). Filtering effects, sequencing depth, and per‐sample missingness did not drive genomic structure based on PCoA (Figures S8−S11).

### 3.2 Population Genomic Structure and Phylogenomics

**FIGURE 2:**
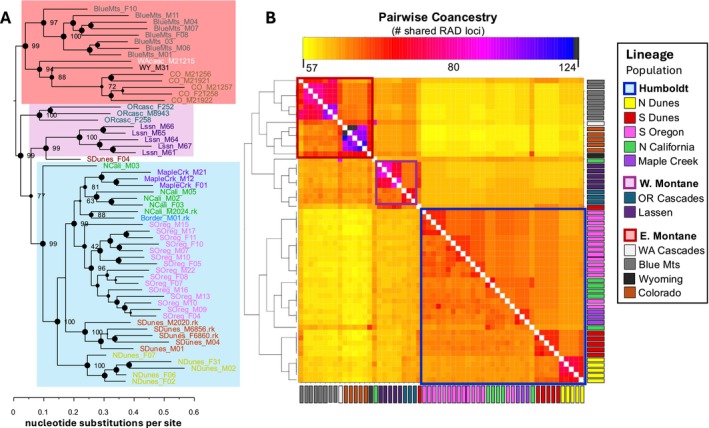
(A) Maximum‐likelihood phylogeny of Pacific martens reflecting identity‐by‐descent relationships based on the low missingness ddRADseq dataset (3586 SNPs). Branch lengths show the number of nucleotide substitutions per site and larger circles at nodes show higher bootstrap support. Bootstrap support (%) is labeled at major splits. Tip labels are colored by population and highlighted boxes identify major lineages. (B) Pairwise coancestry based on fineRADstructure where yellow shows low relatedness and blue/purple shows high relatedness between individuals. The dendrogram on the right reflects identity‐by‐state relationships. Color tabs at the lower and right edges identify sampled population and outlined boxes within the matrix identify major lineages.

**FIGURE 3:**
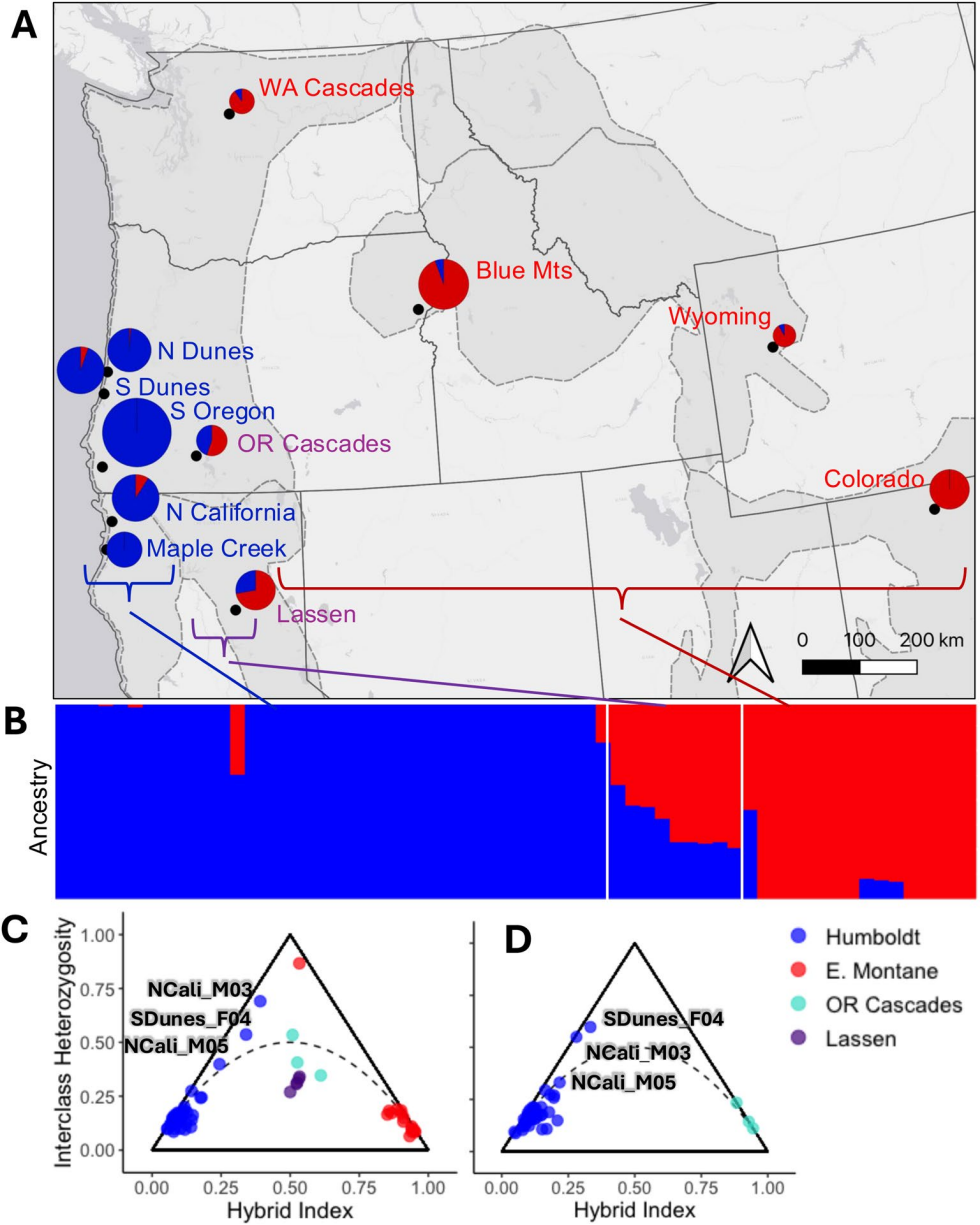
ADMIXTURE results for *K* = 2 based on 12,389 LD‐pruned SNPs where (A) pie charts represent population‐averaged ancestry with chart size scaling with sample size, and (B) the barplot shows cluster assignments for individuals. Triangle plots show that mixed ancestry reflects (C) neutral diffusion in western montane (OR Cascades/Lassen) martens and (D) true hybridization in 2 Humboldt martens (SDunes_F04 and NCali_M03) based on 180 and 197 SNPs, respectively. ADMIXTURE models at higher *K* values and evaluation metrics are found in Figure S12, and corresponding evalAdmix residual plots in Figure S13.

Our genomic structure and phylogenomic results supported major western and eastern lineages within Pacific martens. The first axis of our PCoA showed that 17% of genetic variation within our dataset follows an east–west gradient that splits Humboldt from montane martens east of the Cascades, with Oregon Cascades and Lassen National Forest martens in between (Figure 1C). ADMIXTURE (Figure 3) and fineRADstructure (Figure 2B) models similarly identified two main ancestral clusters aligning with Humboldt and eastern montane lineages, with mixed or intermediate ancestry in the Oregon Cascades and Lassen. Our phylogenomic tree placed the Oregon Cascades and Lassen populations within a western lineage which includes Humboldt martens as a well‐supported clade; this western lineage is split from an ‘eastern montane’ lineage including martens in the Rocky Mountains and Washington Cascades (Figure 2A).

Given narrow 95% confidence intervals (mean range = 0.02; Table S4), we present raw estimates of pairwise *F*_ST_ (Table 1). Humboldt martens had moderate genetic differentiation (Nei's *F*_ST_ = 0.16) from montane martens. Based on F_ST_ of each lineage relative to all Pacific martens (Table 2), Humboldt martens had the highest deviation from a shared ancestral state (*F*_ST_ = 0.295) followed by western montane martens (*F*_ST_ = 0.155) and eastern montane martens, which had low deviation (*F*_ST_ = 0.056). Genetic substructure within each lineage reflected geographical clustering of samples (Figures 1C, 2B, Figures S12−S13). Rangewide isolation‐by‐distance among Pacific marten populations in our dataset was moderately supported by a Mantel test (*r* = 0.53, *p* = 0.001).

**TABLE 1:** Pairwise Nei's *F*_ST_ (Nei 1987) calculated between each pair of geographically defined populations based on ddRADseq and GBAS datasets, with higher values (red) indicating increased genetic differentiation between populations and values at 0 (blue) indicating no genetic differentiation.

	*N*	N. Dunes	S. Dunes	S. Coast	S. Oregon	Applegate, OR	OR/CA Border	N. California	Maple Creek	OR Cascades	Lassen	WA Cascades	Blue Mtns, OR	Wyoming	Colorado
ddRADseq (12,389 SNPs)															
**N. Dunes**	6	—	0.25	—	0.26	—	—	0.24	0.32	0.28	0.38	0.43	0.34	0.47	0.41
**S. Dunes**	7	0.25	—	—	0.15	—	—	0.14	0.23	0.22	0.32	0.36	0.30	0.37	0.38
**S. Oregon**	15	0.26	0.15	—	—	—	—	0.09	0.16	0.21	0.31	0.35	0.30	0.36	0.37
**N. California**	7	0.24	0.14	—	0.09	—	—	—	0.08	0.18	0.27	0.28	0.26	0.30	0.32
**Maple Creek**	4	0.32	0.23	—	0.16	—	—	0.08	—	0.24	0.34	0.39	0.32	0.41	0.40
OR Cascades	3	0.28	0.22	—	0.21	—	—	0.18	0.24	—	0.19	0.18	0.18	0.16	0.25
Lassen	5	0.38	0.32	—	0.31	—	—	0.27	0.34	0.19	—	0.30	0.26	0.30	0.32
WA Cascades	2	0.43	0.36	—	0.35	—	—	0.28	0.39	0.18	0.30	—	0.20	0.13	0.25
Blue Mtns, OR	8	0.34	0.30	—	0.30	—	—	0.26	0.32	0.18	0.26	0.20	—	0.18	0.25
Wyoming	1	0.47	0.37	—	0.36	—	—	0.30	0.41	0.16	0.30	0.13	0.18	—	0.22
Colorado	5	0.41	0.38	—	0.37	—	—	0.32	0.40	0.25	0.32	0.25	0.25	0.22	—
GBAS (98 SNPs)															
**N. Coast OR**	4	0.31	0.27	0.12	0.23	0.33	0.28	0.23	0.32	0.34	0.38	0.42	0.46	0.42	0.51
**N. Dunes**	14	—	0.43	0.32	0.34	0.43	0.40	0.36	0.38	0.37	0.47	0.49	0.52	0.46	0.55
**S. Dunes**	37	0.43	—	0.11	0.15	0.16	0.17	0.14	0.22	0.23	0.29	0.37	0.39	0.35	0.43
**S. Coast OR**	2	0.32	0.11	—	0.02	−0.26	0.04	0.03	0.13	0.16	0.22	0.19	0.33	0.18	0.37
**S. Oregon**	19	0.34	0.15	0.02	—	0.08	0.07	0.05	0.09	0.20	0.24	0.33	0.36	0.31	0.40
**Applegate, OR**	1	0.43	0.16	−0.26	0.08	—	0.14	0.10	0.22	0.25	0.29	0.37	0.46	0.43	0.56
**OR/CA Border**	7	0.40	0.17	0.04	0.07	0.14	—	0.03	0.12	0.21	0.25	0.31	0.35	0.31	0.37
**N. California**	19	0.36	0.14	0.03	0.05	0.10	0.03	—	0.05	0.19	0.24	0.29	0.35	0.29	0.37
**Maple Creek**	5	0.38	0.22	0.13	0.09	0.22	0.12	0.05	—	0.20	0.24	0.29	0.34	0.27	0.40
OR Cascades	9	0.37	0.23	0.16	0.20	0.25	0.21	0.19	0.20	—	0.17	0.14	0.20	0.21	0.30
Lassen	11	0.47	0.29	0.22	0.24	0.29	0.25	0.24	0.24	0.17	—	0.21	0.27	0.28	0.36
WA Cascades	2	0.49	0.37	0.19	0.33	0.37	0.31	0.29	0.29	0.14	0.21	—	0.15	0.11	0.26
Blue Mtns, OR	10	0.52	0.39	0.33	0.36	0.46	0.35	0.35	0.34	0.20	0.27	0.15	—	0.22	0.27
Wyoming	2	0.46	0.35	0.18	0.31	0.43	0.31	0.29	0.27	0.21	0.28	0.11	0.22	—	0.17
Colorado	5	0.55	0.43	0.37	0.40	0.56	0.37	0.37	0.40	0.30	0.36	0.26	0.27	0.17	—

*Note:* Negative values indicate high within‐ relative to between‐population variation and can be interpreted as low differentiation. Confidence intervals are found in Tables S4 and S7. Humboldt marten populations are in bold.

**TABLE 2:** Population genetic metrics based on LD‐pruned ddRADseq (12,389 SNPs) for geographically defined Pacific marten ( *Martes caurina* ) populations including fixation index (*F*_ST_) for each population relative to the broad group (i.e., Humboldt or montane) and for each group relative to the full dataset, population‐level inbreeding coefficients (*F*_IS_), observed heterozygosity (*H*_O_), expected heterozygosity (*H*_S_), average individual genome‐wide heterozygosity (*H*_a_), average individual SNP heterozygosity (*H*_SNP_), allelic richness rarefied to *N* = 3 diploid individuals (*A*_R_), and private allelic richness rarefied to *N* = 3 diploid individuals (*A*_P_).

Population	*N*	*F*_ST_	*F*_IS_	*H*_S_	*H*_O_	*H*_a_	*H*_SNP_	*A*_R_	*A*_P_
**Humboldt**	**39**	**0.30**	**0.27**	**0.18**	**0.17**	**0.0033**	**0.17**	**1.61**	**1.28**
North Dunes	6	0.34	0.32	0.13	0.16	0.0030	0.16	1.29	1.12
South Dunes	7	0.21	0.33	0.15	0.16	0.0030	0.16	1.35	1.11
S. Oregon	15	0.14	0.25	0.16	0.17	0.0033	0.18	1.36	1.14
N. California	7	0.04	0.16	0.18	0.20	0.0037	0.20	1.42	1.14
Maple Creek	4	0.27	0.36	0.14	0.16	0.0029	0.15	1.40	1.23
**Montane**	**24**	**0.03**	**0.09**	**0.25**	**0.21**	**0.0040**	**0.21**	**1.84**	**1.72**
OR Cascades	3	0.17	0.08	0.22	0.21	0.0040	0.22	1.61	1.37
Lassen	5	0.31	0.10	0.18	0.21	0.0039	0.21	1.40	1.18
Blue Mtns, OR	8	0.20	0.01	0.21	0.23	0.0043	0.23	1.47	1.32
WA Cascades	2	0.22	0.20	0.21	0.20	0.0037	0.19	2.00	—
Wyoming	1	—	0.06	—	0.22	0.0041	0.22	2.00	—
N. Colorado	5	0.27	0.19	0.19	0.19	0.0036	0.19	1.42	1.29

We further investigated mixed cluster assignments and intermediate coancestry between montane and Humboldt martens. Triangle plots (Figure 3C) and phylogenomics (Figure 2A) suggested that mixed ancestry in western montane (i.e., Oregon Cascades and Lassen) martens (*N* = 8) was caused by intermediate coancestry rather than true admixture between Humboldt and eastern montane lineages. In contrast, two of three Humboldt martens (NCali_M03 and SDunes_F04 in Figure 3D) with mixed ancestry showed high interclass heterozygosity and moderate hybrid indexes consistent with a first‐generation backcross of Humboldt‐western montane hybrids with pure Humboldt martens (Wiens and Colella 2024), while the remaining individual (NCali_M05 in Figure 3D) showed patterns consistent with neutral diffusion. Evidence for the origin of this gene flow was conflicting: phylogenomics showed that Humboldt martens share a most recent common ancestor with Lassen martens but genetic structure based on fineRADstructure showed higher coancestry between putative hybrids and Oregon Cascades martens (Figure 2B). Further, F_ST_ values were lower between Oregon Cascades and Humboldt populations relative to Lassen, indicating less genetic differentiation (Table 1).

**FIGURE 4:**
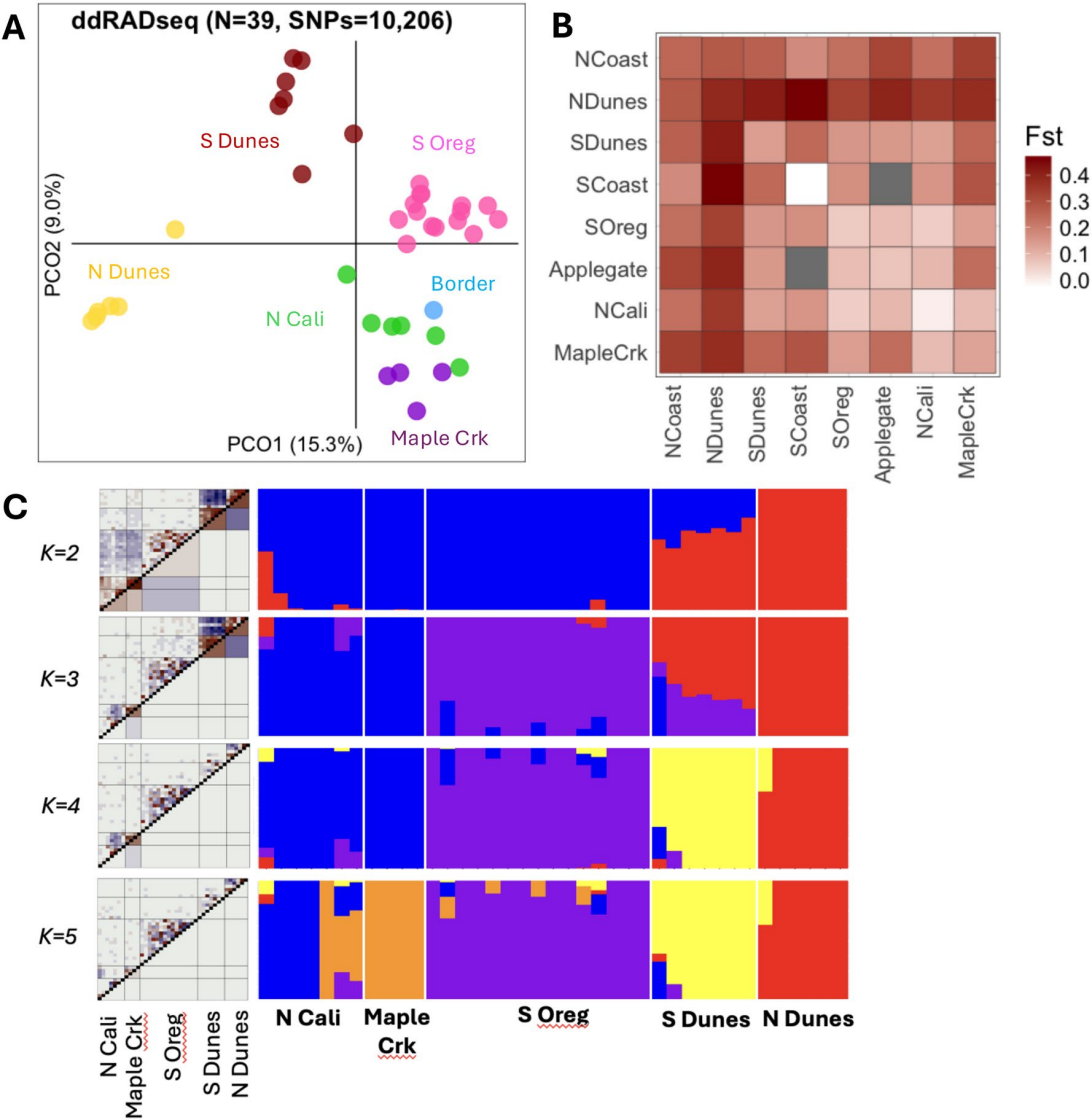
Genetic structure in Humboldt martens based on: (A) PCoA of the high‐missingness ddRADseq dataset, (B) genetic differentiation between sampling regions as estimated by pairwise *F*_ST_ from GBAS data (*N* = 108 individuals, 98 SNPs), and (C) ADMIXTURE results based on LD‐pruned ddRADseq dataset (9239 SNPs in Humboldt martens) on the right and correlations in model residuals based on evalAdmix on the left. ADMIXTURE model results at higher *K* values can be found in Figure S14 and corresponding evalAdmix residual plots in Figure S15.

Within Humboldt martens, our genomic data suggested four clusters supported by PCoA (Figure 4A), fineRADstructure (Figure 2B), and ADMIXTURE (Figure 4C) that align with the contemporary North Dunes, South Dunes, Southern Oregon, and Northern California plus individuals from a new ‘Maple Creek’ group located to the south, with some support for Maple Creek individuals forming a fifth cluster. A roadkill marten located between the Northern California and Border population areas clustered closely with Northern California samples in all analyses and was binned with Northern California samples for population‐level calculations. Martens in Northern California retain most genetic variation observed in the subspecies (*F*_ST_ = 0.05), and other populations show increasing deviation from shared Humboldt genetic variation (*F*_ST_, Table 2) and increasing genetic differentiation between populations when moving away from Northern California (Table 1).

Genetic structure and signatures of isolation increased moving away from Northern California. Specifically, Northern California showed low differentiation from adjacent Southern Oregon and Maple Creek populations (Table 1). Southern Oregon Humboldt martens formed a relatively distinct cluster and clade, yet some individuals shared ancestry with Northern California Humboldt martens (Figures 1C, 2, 4C). In contrast, the Dunes populations showed the highest deviation from the shared Humboldt state (population‐specific *F*_ST_ for N Dunes = 0.34, S Dunes = 0.21), and genetic differentiation between these populations was high (pairwise *F*_ST_ = 0.25) despite moderate levels of coancestry (Figure 2B). The North Dunes population had high levels of differentiation compared to all other Humboldt marten populations (*F*_ST_ ≥ 0.24) and formed a highly distinct cluster (Figure 4). High within‐population coancestry (Figure 2B) in the North Dunes, South Dunes, Maple Creek, and groups of Southern Oregon martens likely reflects shared kinship.

### 3.3 Inbreeding and Genetic Diversity

**FIGURE 5:**
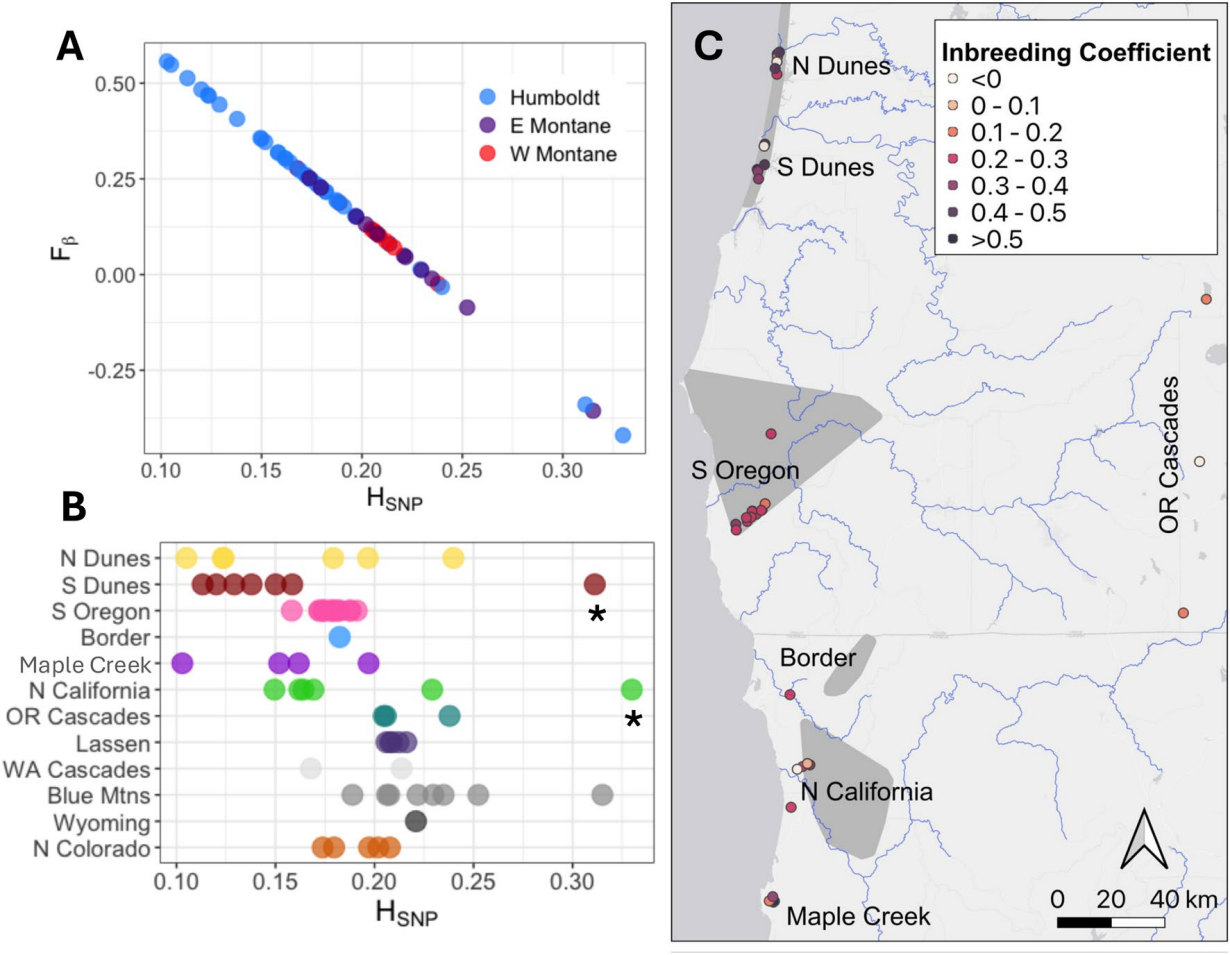
(A) Individual genetic diversity versus individual inbreeding estimates based on LD‐pruned ddRADseq data showing high correlation. (B) Individual heterozygosity estimates grouped by population, with putative hybrids marked with stars. (C) Map of individual inbreeding coefficients for Humboldt martens and nearby western montane martens in the Oregon Cascades showing highest inbreeding in the Dunes and Maple Creek populations. Humboldt martens with *F*_*β*_ < 0 indicate outbreeding (i.e., hybrids).

**FIGURE 6:**
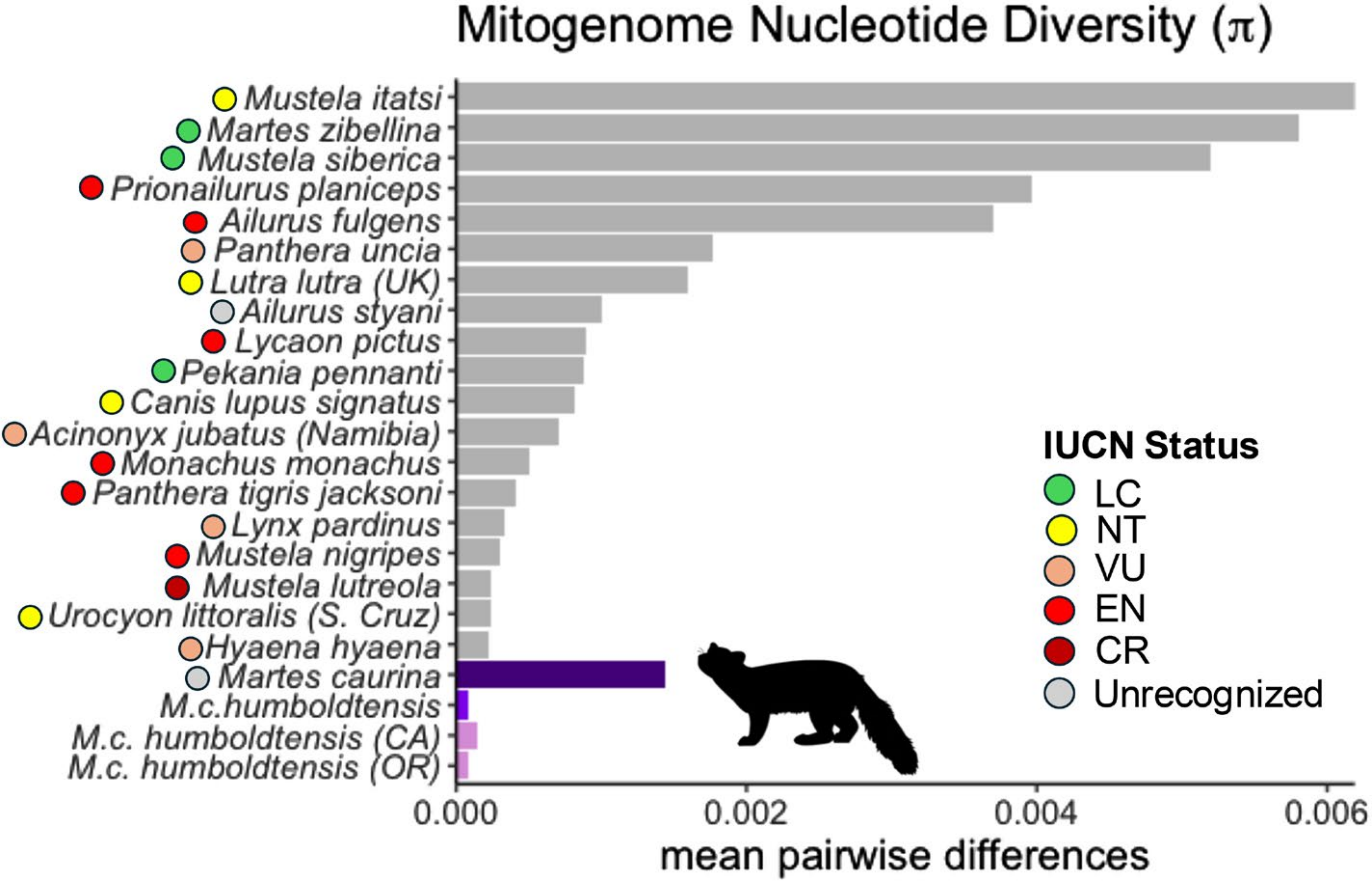
Mitogenome nucleotide diversity of Pacific martens (dark purple), Humboldt martens (medium purple), and Humboldt marten subpopulations (light purple) relative to published values for other carnivore species, including genetically depauperate species and populations. Subspecies and population‐level estimates were used whenever available to better enable comparison; sample sizes are found in Table S8. Common names and sources in order: Striped hyena: *Hyaena hyaena* (Westbury et al. 2018); Santa Cruz island gray fox: *Urocyon littoralis* (Hofman et al. 2015); European mink: *Mustela lutreola* (Skorupski et al. 2025); black‐footed ferret: *Mustela nigripes* (Etherington et al. 2022); Iberian lynx: *Lynx pardinus* (Casas‐Marce et al. 2013); Malayan tiger: *Panthera tigris jacksoni* (Liu et al. 2018); Mediterranean monk seal: *Monachus monachus* (Rey‐Iglesia et al. 2021); African cheetah (Namibia population): *Acinonyx jubatus* (Dobrynin et al. 2015); Iberian wolf: *Canis lupus signatus* (Salado et al. 2023); fisher: *Pekania pennanti* (Knaus et al. 2011); African wild dog: *Lycaon pictus* (Tensen et al. 2025); Chinese red panda: *Ailurus styani* and Himalayan red panda: *Ailurus fulgens* (Hu et al. 2020); United Kingdom Eurasian otter: *Lutra lutra* (du Plessis et al. 2025); snow leopard: *Panthera uncia* (Wang et al. 2025); flatheaded cat: *Prionailurus planiceps* (Patel et al. 2017); Siberian weasel: *Mustela siberia* and Japanese weasel: *Mustela itatsi* (Shalabi 2016); and sable: *Martes zibellina* (Li et al. 2021).

Population‐level estimates based on ddRADseq showed low to moderate levels of inbreeding across all Pacific marten populations, but Humboldt marten populations (except for Northern California) had higher inbreeding and lower genetic diversity than montane marten populations (Table 2). Individual Humboldt martens had SNP heterozygosity ~20% lower on average than montane martens and individual inbreeding coefficients about three times higher on average, although individual variation was higher in Humboldt than montane martens (Figure 5). Mitogenome nucleotide diversity showed similar patterns of higher mitogenome nucleotide diversity estimates in most montane marten populations compared to Humboldt martens (Table 3), which showed levels lower than other genetically imperiled populations (Figure 6; Table S8).

**TABLE 3:** Estimates of mitochondrial nucleotide diversity (π), the average number of pairwise differences between individuals' mitogenomes, and its standard deviation (SD) within Pacific marten ( *Martes caurina* ) populations based on previously published mitochondrial genomes.

Population	*N*	π	SD
**OR Coast** ^a^	23	7.88E‐05	5.75E‐05
**CA Coast** ^a^	16	1.41E‐04	9.16E‐05
OR Cascades^a^	22	3.71E‐04	2.05E‐04
CA Shasta^a^	7	5.35E‐05	4.88E‐05
CA Sierra^a^	8	7.45E‐04	4.30E‐04
Olympia^a^	7	1.51E‐04	1.06E‐04
WA Cascades^a^, ^b^	8	1.82E‐03	1.02E‐03
Lassen^a^	4	1.83E‐03	1.22E‐03
Blue Mountains^a^	2	0^c^	0^c^

*Note:* Humboldt marten populations in bold.

^a^ Schwartz et al. (2020).

^b^ Colella et al. (2019).

^c^ Note low sample size in this population.

Individual inbreeding and heterozygosity estimates had a strong negative correlation (r = −0.99). Most montane martens had relatively high heterozygosity (*H*_SNP_ > 0.2) and low inbreeding coefficients (*F*_β_ < 0.15), with similar values in Humboldt martens only observed in the North Dunes and putatively admixed individuals (Figure 5). The lowest heterozygosities (*H*_SNP_ < 0.15) and highest inbreeding coefficients (most with *F*_β_ > 0.40) occurred in the North Dunes, South Dunes, and one marten from Maple Creek. Southern Oregon, Northern California, and other Maple Creek martens generally had moderate heterozygosity (*H*_SNP_ 0.15–0.20) and inbreeding (*F*_β_ 0.17–0.35) which overlapped with estimates in some montane martens. Heterozygosity estimates were not affected by sequencing depth (Figure S16).

### 3.4 GBAS Panel Development & Assessment

Our in silico optimization identified a 150‐plex with 23 predicted dimers from 2469 candidate loci. We ordered 147 primer pairs after in silico specificity screening. During initial lab testing on scats, we removed 38 primer pairs due to primer interactions affecting amplification success in scats, 11 pairs that had low or no amplification, and six pairs that amplified putative paralogs (i.e., non‐unique regions within the genome) based on high (> 4) SNP density within the amplicon. We added two primer pairs targeting the sex‐determining region Y (SRY) for sex identification. Our optimized 94‐locus panel had 92 SNP amplicons including six microhaplotypes (98 total SNPs) and two sexing loci (details in Supporting Information S7, Table S6). ddRADseq allele frequencies suggested high resolution for individual identification within Humboldt martens (population‐level PID_SIB_ 2.6 × 10^−14^–5.4 × 10^−13^).

**FIGURE 7:**
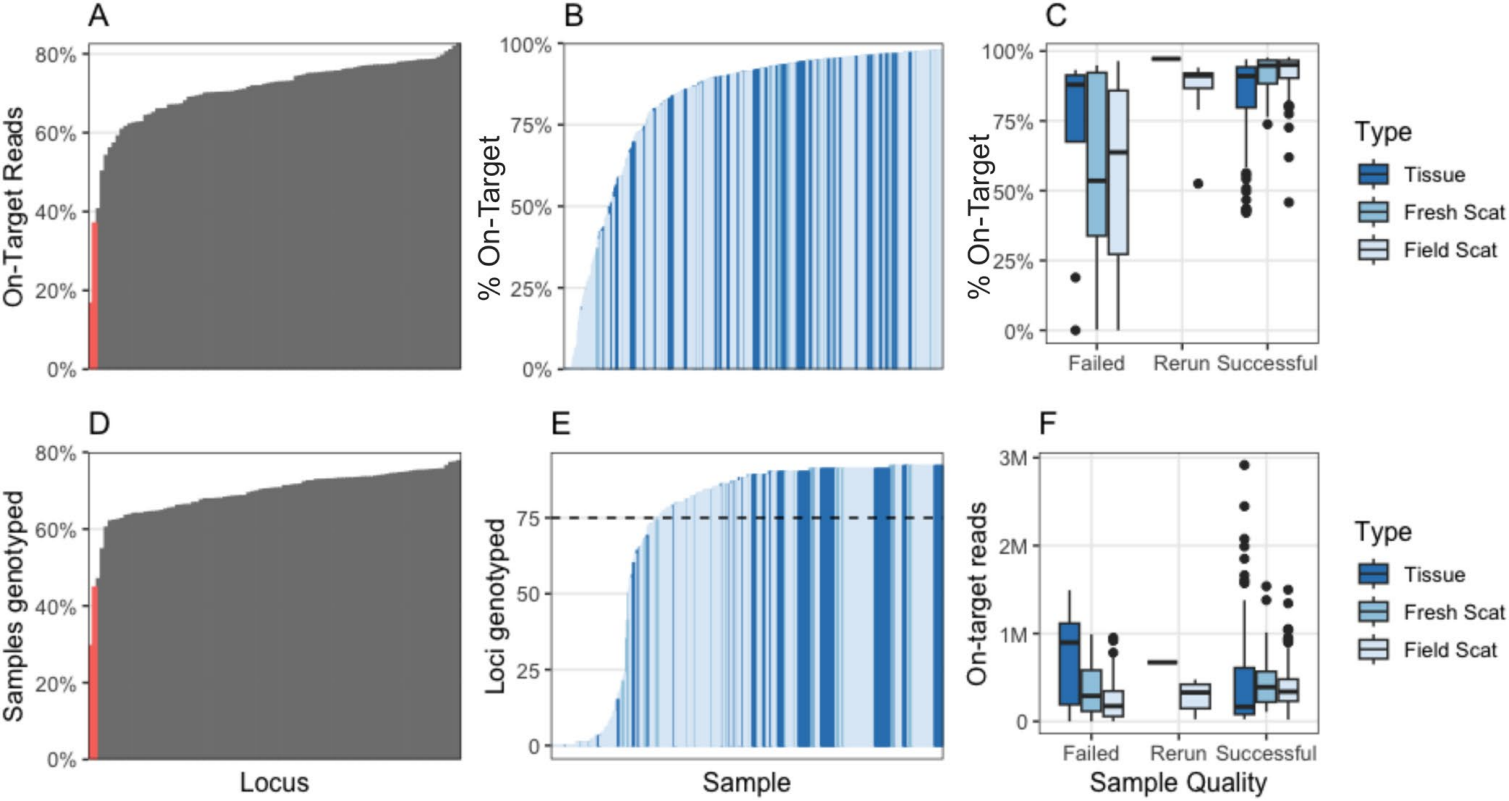
GBAS panel performance as demonstrated by high on‐target amplification and genotyping rates across loci (A and D), samples (B and E), and sample qualities (C and F) where “Successful” samples are those that initially genotyped at ≥ 75 SNPs, “Rerun” samples successfully genotyped only with additional PCR replicates, and “Failed” samples genotyped at < 75 SNPs. Per‐locus on‐target rates were calculated as the mean percent of on‐target reads across all samples.

The final GBAS panel had high amplification success, low genotyping error, and correctly assigned scats to known individuals. Our trapping‐collected scats successfully genotyped at ≥ 75 SNPs with 80% success (39/49). All 39 scats from known individuals were accurately identified and sexed. Scats generally had > 85% on‐target rates (i.e., the percent of sequencing reads belonging to a locus targeted by PCR) (Figure 7A) and 90/92 SNP amplicons showed genotyping rates > 60% (Figure 7D). The mean genotyping error rate in PCR replicates was 0.0002 for tissue samples (allele dropout rate [AD] = 0.0007, false allele rate [FA] = 0) and 0.038 for scat samples (AD = 0.13, FA = 0.0009). When comparing consensus genotypes between tissue and scats collected from known individuals, the mean genotyping error rate per fresh scat sample was 0.014 (SD = 0.038; AD = 0.015, FA = 0.0003) with an average of about 1 allele dropout per scat. Mean per‐SNP genotyping error in scats was 0.008 (SD = 0.018, AD = 0.034, FA = 0.009). Genotyping error rates, especially allele dropouts, increased substantially in marginal quality samples with lower genotyping success (i.e., < 75 SNPs genotyped). Our loci amplified in American marten (88 SNPs), fisher (52 SNPs), mink (20 SNPs), and red‐backed vole (1 SNP) samples, but not in deer mice, Townsend's chipmunk, or gray fox scats. All non‐marten carnivore amplification was homozygous, and most amplicons had ≥ 1 bp difference from martens. One locus amplified in western red‐backed vole tissue but the amplicon was distinct from marten.

Based on our data, the GBAS panel has high resolution for individual identification and moderate resolution for population assignment. All populations had PID and PID_SIB_ < 10^−8^ (Table 4) suggesting high resolution for individual identification across Pacific martens. Three SNPs were apparently fixed in montane martens but variable in Humboldt martens, and the probability of a Humboldt marten sharing the genotype of a montane marten at these sites is ~1%, indicating that these markers combined have reasonable power to distinguish Humboldt from montane martens. All populations had > 99% correct population assignment of simulated individuals (Table 4) with most incorrect assignments to Northern California regardless of population of origin, although small sample sizes of inbred individuals likely drove overestimation of *f*_ORCA_ given no clear differences between some populations based on genetic variation from GBAS (Figure 1D). Mean per‐SNP information content (*I*_n_) was 0.22 (SD = 0.12). Polymorphic loci in the GBAS panel and individual heterozygosity generally declined with increasing distance from Humboldt martens (Table 4) indicating lower information content, although sample sizes in montane populations were often small. Fewer SNPs were also observed in northern Humboldt marten populations on the Dunes, possibly caused by SNP discovery using few and closely related individuals.

**TABLE 4:** Metrics of GBAS panel information content per population including the number of individuals per population (*N*), number of polymorphic loci and SNPs, mean SNP heterozygosity (*H*_SNP_), theoretical probability of identity (PID, the probability that two random individuals within a population have the same genotype), unbiased probability of identity (PID_UB_) which accounts for sample size, probability of identity of siblings (PID_SIB_, the probability of two full siblings sharing the same genotype), and *f*_ORCA_ (the simulated proportion of individuals assigned to the correct population).

Population	*N*	Loci	SNPs	*H*_SNP_	PID_SIB_	PID_ub_	PID	*f*_ORCA_
Humboldt	109	92	96	0.31	1.70E‐16	6.20E‐32	1.50E‐31	—
N Coast	4	58	58	0.26	4.10E‐10	NA	4.80E‐19	—
N Dunes	16	59	60	0.25	1.90E‐10	1.00E‐21	1.20E‐16	1.000
S Dunes	45	84	88	0.29	8.40E‐13	2.30E‐25	2.20E‐24	0.995
S Oregon	18	83	85	0.33	1.50E‐14	7.10E‐30	1.20E‐27	0.996
N California	19	87	89	0.35	5.10E‐15	8.10E‐31	1.30E‐28	0.993
Maple Creek	5	74	77	0.31	5.60E‐13	5.70E‐41	1.10E‐24	—
Montane	39	87	91	0.27	7.20E‐16	3.70E‐31	3.80E‐30	—
OR Cascades	9	82	78	0.34	1.70E‐14	7.20E‐32	1.90E‐27	1.000
Lassen	11	81	77	0.29	1.10E‐12	2.80E‐27	5.70E‐24	1.000
WA Cascades	2	46	49	0.24	3.70E‐10	NA	7.30E‐19	—
Blue Mountains	10	58	62	0.23	2.30E‐11	3.20E‐24	2.50E‐21	1.000
WY	2	48	51	0.30	3.30E‐10	NA	3.70E‐19	1.000
Colorado	5	47	50	0.22	6.70E‐09	5.60E‐23	1.10E‐16	1.000

### 3.5 GBAS Panel Validation

**FIGURE 8:**
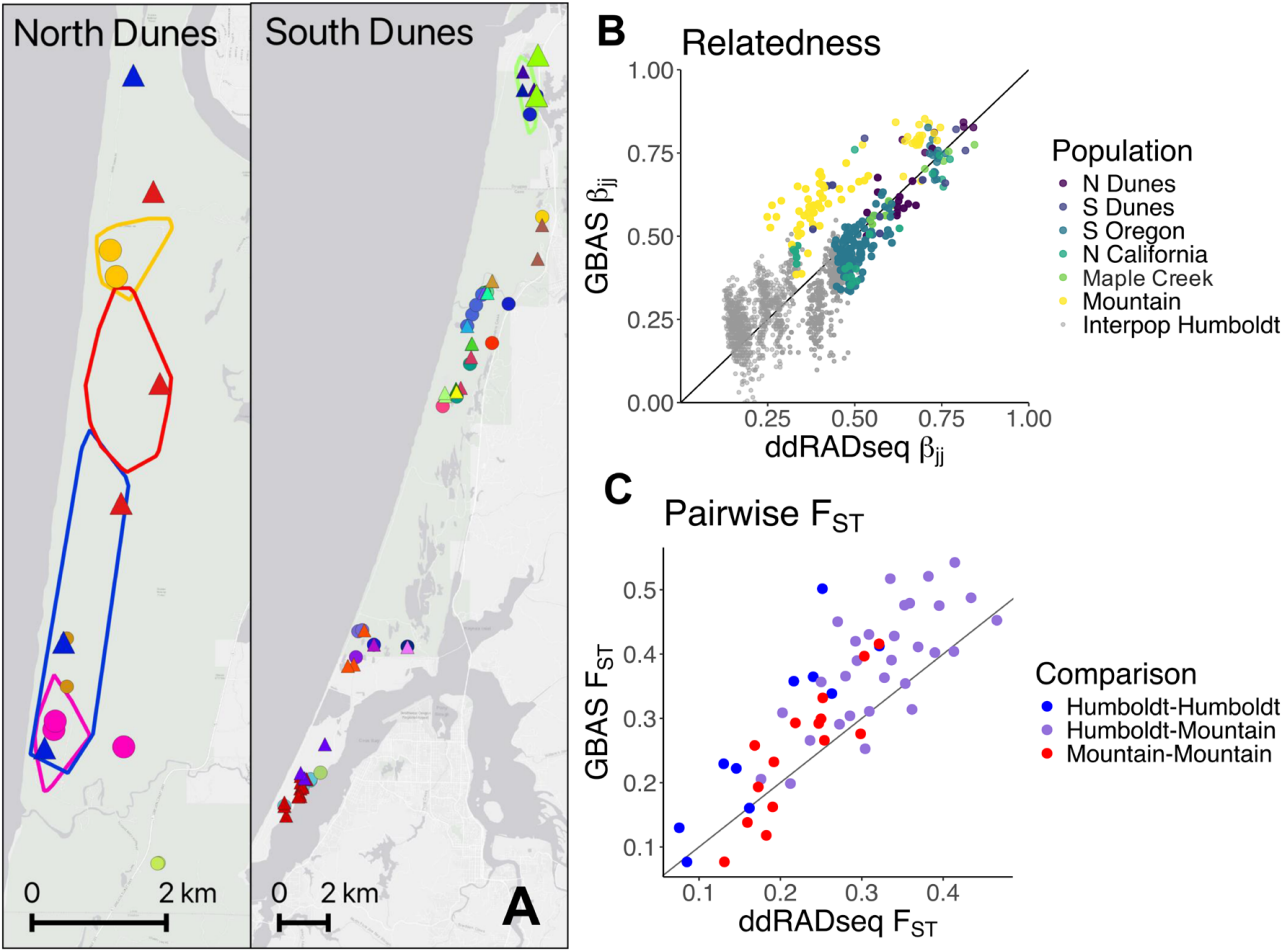
We validated GBAS results by checking whether scats from genotyped individuals were geographically plausible (A) and by assessing concordance of estimates between GBAS and ddRADseq datasets for 57 individuals (B and C). (A) Maps of genotyped scats (female = circle, male = triangle, colors represent unique individuals) on the North and South Dunes showing alignment of genotyped scats from known individuals (larger points) with known home ranges (polygons; GPS data from Linnell et al. 2018). (B) Individual relatedness estimates for GBAS and ddRADseq with 1:1 line where colors represent intra‐population comparisons and small gray points represent inter‐population comparisons within Humboldt martens. (C) Pairwise *F*_ST_ estimates for GBAS and ddRADseq with 1:1 line where colors represent comparisons of populations within Humboldt martens (blue), within montane martens (red), or one population within each group (purple).

Fifty‐nine individuals were successfully genotyped by both ddRADseq and GBAS. Concordance between datasets showed moderate to high resolution of GBAS for estimating genetic structure and relatedness in Humboldt martens. The first two PCoA axes were highly correlated between ddRADseq and GBAS (PCO1: *r* = 0.95, PCO2: *r* = −0.88) with the first axis effectively splitting Humboldt and montane martens. Clusters in the GBAS PCoA were less distinct, especially in eastern montane martens, suggesting lower power for detecting finer‐resolution genetic structure (Figure S17). Pairwise *F*_ST_ estimates between populations had high correlation (*r* = 0.83) between GBAS and ddRADseq datasets (Figure 8C), although GBAS tended to overestimate *F*_ST_ values for the North Dunes (Table 1). Pairwise relatedness (β_jj_) had moderate overall correlation between GBAS and ddRADseq estimates (*r* = 0.59) but higher correlation when only considering Humboldt martens (r = 0.69; Figure 8B). GBAS overestimated β_jj_ in montane martens and trends were imperfect and varied by population within Humboldt martens, including some overlap in GBAS β_jj_ estimates between individuals from different populations (e.g., Southern Oregon, Figure S18), indicating that the GBAS panel should be used only for broad trends in relatedness with context‐specific interpretation. The GBAS panel was unsuitable for estimating individual heterozygosity and inbreeding (*H*_SNP_: *r* = −0.09, *F*_β_: *r* = −0.09; Figure S19).

Contemporary effective population size (*N*_e_) estimates were generally consistent between ddRADseq and GBAS datasets, although precise estimates for GBAS required larger sample sizes given lower marker information content (Table 5). Otherwise, *N*_e_ estimates were imprecise when a small proportion of the population was sampled. *N*_e_ estimates based on the GBAS dataset were relatively consistent across years, although *N*_e_ point estimates in the South Dunes increased with sample size suggesting that larger samples are needed to avoid underestimation. Our estimates suggest low effective population sizes in the North and South Dunes and Southern Oregon, and higher but more uncertain estimates in Northern California. Our *N*_e_ estimates are biased by overlapping generations, failure to account for migration (Ryman et al. 2019), and spatially clustered sampling of related individuals in Southern Oregon and Northern California (Waples 2024). We expect our *N*_e_ estimates for the South Dunes, however, to be relatively accurate given isolation, large sample sizes relative to *N*_e_, and similarity of estimates over time.

**TABLE 5:** Effective population size estimates for each population‐year based on CurrentNe applied to the LD‐pruned low missingness ddRADseq dataset and NeEstimator v2.1 applied to the GBAS dataset, associated sample and marker set sizes, and confidence intervals.

Population	Year	ddRADseq (CurrentNe)				GBAS (NeEstimator)			
		*N*	SNPs	*N*_e_	90% CI	*N*	SNPs	*N*_e_	95% CI
**N Dunes**	2015	5	802	8.6	5.0–15.2	14	62	4.1	2.5–12.8
**S Dunes**	2015	5	1095	10.8	5.8–19.9	9	84	3.4	2–11.2
	2017	—	—	—	—	16	71	14.4	6.5–35.7
	2020	—	—	—	—	13	82	10.7	3.9–26.9
**S Oregon**	2023	14	1411	13.9	10.6–18.4	19	91	20.7	12.1–40.5
**N California**	2020	6	1570	24.1	11.8–49.2	10	87	35.4	7.4 – ∞
	2022	—	—	—	—	13	89	36.5	15.9–466
**Maple Creek**	2022	—	—	—	—	5	80	9.2	1.6 – ∞
Lassen	2024	5	1322	5.5	5.0–8.7	7	67	18.9	7.2–276
Blue Mtns	—	8	1841	15.2	9.5–24.3	10	64	77.8	26.5 – ∞
Colorado	—	5	1366	68.9	20.5–232	5	50	∞	9.2 – ∞

*Note:* Infinite estimates often reflect small sample size relative to *N*_e_. Humboldt marten populations in bold.

### 3.6 Baseline Scat‐Based Humboldt Marten Monitoring

We successfully genotyped 182 of 306 noninvasively collected scats (58%) at ≥ 75 SNPs during initial genotyping with 3 PCR replicates. After rerunning 44 samples with 3 more PCR replicates, we successfully genotyped an additional 27 scats for a final success rate of 68%. Genotyping success was substantially lower in scats collected during rainy (60%) compared to summer (88%) months. Samples had similar read counts (Figure 7F) and on‐target rates (Figure 7B,C) across sample types and quality, but on‐target sequences in failed/rerun samples were attributed to fewer loci (Figure 7E), suggesting that panel failure may be caused by degradation of target DNA rather than primer interactions, inhibitors, or non‐target DNA.

We identified 36 females and 35 males from successfully genotyped, noninvasively collected samples, including matching genotypes of four females and three males to tissue samples from martens live‐trapped on the Oregon Dunes in 2016–17 (Moriarty et al. 2017; Linnell et al. 2018). Scat detections of these individuals aligned within or near their respective home ranges (Figure 8A). Detections outside of known population areas included four females north of the Oregon Dunes (North Coast), two males around Port Orford, OR (South Coast), and one male near Applegate, OR 45 km eastward of other Humboldt marten detections (Figure 1A). Consistent with the spatial ecology of the species (Moriarty et al. 2017), distances between detections per individual were larger for males (2.0 ± 2.7 km, range 0.13–8.5, *N* = 13) than females (0.42 ± 0.44 km, range 0.01–1.4, *N* = 13). Our full GBAS dataset included genotypes from 148 individuals, including 59 individuals which had high‐quality samples represented in ddRADseq (*N* = 59) plus 89 individuals identified from noninvasively collected scats alone (*N* = 63), hair collected via baited hair snares (*N* = 2), and tissue samples with insufficient DNA quantity and quality for RADseq (*N* = 24).

Population genetic structure based on GBAS supported ddRADseq results: Rangewide PCoA results showed a similar pattern to ddRADseq (Figure 1) where most genetic variation aligned along an east–west axis which effectively separated Humboldt from montane martens. Within Humboldt martens, the North Dunes, South Dunes, and North Coast populations clustered separately from others (Figure S20), driven by monomorphic SNPs in these populations which are likely the result of genetic drift and inbreeding. Pairwise F_ST_ values showed high differentiation of the North Dunes and moderate differentiation of the South Dunes from all other populations, low differentiation of Maple Creek from other populations except Northern California, and little to no differentiation between Southern Oregon and Northern California populations. Our GBAS maximum‐likelihood phylogeny (Figure S21) showed consistency with broad relationships seen in the ddRADseq phylogeny (Figure 2A), but branching topology had low support and placement of Humboldt martens within clades was only consistent for the Dunes populations.

GBAS results further expanded genetic inference through broader spatial sampling. PCoA revealed four putatively admixed individuals between Humboldt and montane martens, including the two individuals identified as likely hybrids by ddRADseq and two additional individuals including a roadkill female from the South Dunes that clustered with montane martens and a male marten in the Oregon Cascades that clustered with Humboldt martens (Figure 1D). North Coast individuals clustered between the North Dunes and other Humboldt martens (Figure S21, Figure 1D), suggesting shared coancestry with the North Dunes despite high pairwise differentiation indicating lack of connectivity. South Coast and Applegate individuals clustered at the edge of the Southern Oregon‐Northern California group (Figure 1D). Low pairwise *F*_ST_ (Table 1) with Northern California, the Border, and Southern Oregon suggests an ambiguous origin for the Applegate individual, although the maximum‐likelihood phylogeny (Figure S21) suggests minimum genetic distance to Southern Oregon, South Coast, and Border individuals based on GBAS. Low F_ST_ between the North and South Coast is likely due to small sample sizes in both populations.

## 4 Discussion

We filled conservation‐relevant knowledge gaps in threatened Humboldt martens by maximizing information content from few high‐quality and many low‐quality DNA samples. We provided the first descriptions of extremely low genetic diversity, hybridization with montane martens, and geographic structuring of genetic viability in Humboldt martens. Although our genomic analyses were limited by small sample sizes and clustered sampling, genotyped scats expanded spatial coverage of sampling and produced broad population structure inference consistent with ddRADseq. We highlight the power of reduced SNP panels to fill key demographic and genetic knowledge gaps and provide for effective monitoring and management of an elusive and imperiled species.

### 4.1 Noninvasive Genetic Sampling for Fragmented Species Using GBAS

To facilitate broad‐scale population monitoring of Humboldt martens, we designed, tested, and rigorously validated a novel SNP‐based panel for genotyping fecal DNA. Our overall genotyping success rate of 68% of scats at ≥ 75 SNPs without prior screening for target‐specific DNA content considerably exceeds microsatellite‐based success rates reported for Pacific marten scats (17%; Moriarty et al. 2018), reflecting advantages of shorter targets for degraded DNA. Our reduced genotyping success during wet season collections (60%) compared to summer (88%) was consistent with experimental findings for European pine marten, where DNA amplification failure for scats exposed to rainfall increased from 28% to 65% over a 16‐day period (Kubasiewicz et al. 2016), and we recommend adjusting survey timing accordingly. By leveraging low‐quality tissue samples and previously untapped genetic information in scat samples, we more than tripled our sample size of individual Humboldt martens and more than doubled that of western montane martens. We add to a growing body of literature (Natesh et al. 2019; Hayward et al. 2022; Eriksson et al. 2020; Burgess et al. 2022; Solari et al. 2025) emphasizing the efficiency and effectiveness of optimized GBAS panels for noninvasive genetics in wildlife. Although our GBAS panel is smaller than other panels (Natesh et al. 2019; Hayward et al. 2022; Burgess et al. 2022; Solari et al. 2025), reflecting a focus on accurate and cost‐effective individual identification, we demonstrate the utility of a moderate number of SNPs for broad and exploratory population structure inference and demographic estimation (Figure 8, Table 5).

Our GBAS panel also showed ascertainment bias, particularly when applied to non‐target eastern montane martens, driven by strong genetic structure within our system (Table 4). We provide the following recommendations for developing and applying reduced genotyping panels for individual identification in genetically depauperate species with strong population structure: (1) Select markers based on allele frequencies within each population rather than overall allele frequencies to help avoid allele fixation. (2) Include more markers when designing panels intended for application on populations unrepresented during the design process to mitigate against allele fixation. (3) Prior to application in non‐target populations, test panels with samples from unique individuals to ensure reasonable power and accuracy for individual identification, particularly when panels are small and population structure is strong. (4) Unless validated, we recommend against applying reduced SNP panels for population genetic inference in fragmented systems given the high risk of ascertainment bias (Clark et al. 2005; Lachance and Tishkoff 2013), especially for genetic diversity and inbreeding metrics which are directly affected by marker selection.

Genotyping scats expands the toolbox for researching Humboldt martens. We expect that our GBAS panel can be applied to estimate density, demographic rates, broad patterns of gene flow, and coarse spatial ecology and movement patterns (Figure 8A). We found that detection dog teams generally detected multiple martens ($\bar{X}$ = 2 martens/survey, SD = 1.5, range = 1–7) multiple times during each survey ($\bar{X}$ = 2.5 scats/marten/survey, range = 1–12), suggesting that properly standardized scat detection dog surveys and multiple visits paired with GBAS genotyping will provide sufficient data for spatial capture‐recapture estimates of abundance and demography. We also showed that our GBAS panel produces reasonable estimates of effective population size (*N*_e_) given sufficient sampling. Further, *N*_e_ estimates based on noninvasive sampling enable broader and more representative sampling of populations, thereby overcoming small sample sizes and spatial constraints which may bias *N*_e_ estimates in tissue‐based genetic datasets like ddRADseq (Table 5). Our GBAS panel can also be used to monitor genomic erosion in Humboldt martens by tracking population size and isolation in terms of both genetic differentiation and documented migration (Leroy et al. 2018). Future work may focus on modular SNP panel design for higher‐resolution estimation of parameters such as kinship for pedigree reconstruction (e.g., Ekblom et al. 2021) and identification of hybrid order between Humboldt and montane martens.

### 4.2 Pacific Marten Population Structure

Our results provide additional nuclear genomic DNA support for previously described phylogeographic patterns (Schwartz et al. 2020). Specifically, we confirmed western and eastern lineages in Pacific martens, additional genetic structuring within each lineage aligning with geography, and that Humboldt martens form a genetically distinct clade from nearby western montane martens in the Oregon Cascades and Lassen (Figures 1, 2, 3). We developed an updated historical distribution for Humboldt martens (*M. c. humboldtensis*) that incorporates molecular information (Slauson et al. 2009; Schwartz et al. 2020; and herein), historical detection records (Zielinski et al. 2001), and historical forest cover (Oregon Natural Heritage Program) (Supporting Information S1; Figure S1; Appendix S1). Our analyses include previously uncharacterized Rocky Mountain populations, and we revealed that Pacific martens in eastern Oregon and the Washington Cascades were most closely related to Rocky Mountain martens. Projection of species distribution models onto Pleistocene environmental conditions could be used to explore how potential glacial refugia and suitable conditions drive complex phylogeographic patterns (e.g., Quinn et al. 2024) in Pacific martens. We recommend additional sampling in the Rocky Mountains, Canada, and Alaska to fully resolve outdated subspecies boundaries (Hall 1981) in Pacific martens.

### 4.3 Evidence of Hybridization Between Oregon Cascades and Humboldt Martens

Consistent with previous microsatellite results (Schwartz et al. 2020), we found genomic evidence for gene flow from western montane martens in the southern Cascades/Siskiyou region into Humboldt martens. We identified signatures consistent with first‐generation backcrossing in one South Dunes female and one Northern California male (Figure 3D). Although less sensitive, GBAS results showed two additional putatively admixed individuals in the South Dunes and Oregon Cascades (Figure 1D). This Oregon Cascades male was located near Upper Klamath Lake ~65 km northeast of the easternmost detection of Humboldt martens near Ashland, OR (Schwartz et al. 2020; Moriarty et al. 2021); two other martens within 20 km had no indications of admixture based on GBAS. Gene flow between the Oregon Cascades and Humboldt martens is plausible given continuous forest landcover and extreme natural dispersal capacity of martens (occasionally > 100 km in American martens, Johnson et al. 2009) and aligns with a hybrid zone hotspot driven by secondary contact following divergence in Pleistocene glacial refugia (Swenson and Howard 2005). Gene flow from the Oregon Cascades may improve viability in Humboldt martens via natural genetic rescue (Frankham 2015; Clarke et al. 2024) and outbreeding depression, though possible, is unlikely given that these lineages are closely related and occur in similar environments (Frankham et al. 2011).

### 4.4 Genetic Diversity and Inbreeding in Humboldt Martens

Our estimates indicate baseline levels of inbreeding in all Pacific martens sampled, likely reflecting strong population structure and low abundances throughout the species' evolutionary history (Small et al. 2002; Dawson et al. 2017; Colella et al. 2021). Genetic bottlenecks during a species' evolutionary history can have both negative (Schmidt‐Küntzel et al. 2018) and positive (Robinson et al. 2022; Kyriazis et al. 2023) effects on fitness consequences of recent bottlenecks. Future research on the demographic history of Humboldt martens is necessary to understand genetic diversity and population structure within the subspecies prior to anthropogenic impacts.

Compared to montane martens, nuclear genomic diversity in Humboldt martens was extremely low and inbreeding severe in some individuals. This is corroborated by lower mitogenome nucleotide diversity in Humboldt compared to other Pacific marten populations (Table 3) even though evolutionary mechanisms differ between nuclear genomes and maternally inherited mitogenomes. Mitogenome nucleotide diversity for Pacific martens is comparable to other carnivores of conservation concern, but the Humboldt subspecies had about one‐third the level of diversity observed in the most genetically depauperate carnivore populations (Figure 6) including striped hyenas ( *Hyaena hyaena* ; Westbury et al. 2018), Iberian lynx ( *Lynx pardinus* ; Skorupski et al. 2025), and Channel Island gray foxes ( *Urocyon littoralis* ; Hofman et al. 2015). Low genetic diversity and high inbreeding levels likely threaten short‐term individual fitness (Reed and Frankham 2003) and long‐term adaptive potential (Hoffmann et al. 2017) in Humboldt martens.

### 4.5 Genetic Structure and Viability in Humboldt Martens

Genetic viability is geographically structured in Humboldt martens. The apparent subspecies stronghold is in Northern California and supports viability of surrounding populations, and the lowest viability is in the isolated Central Coast Oregon populations. Despite extinction concerns and subsequent rediscovery in 1996 (Kurcera et al. 1996; Zielinski et al. 2001), Northern California martens preserve the greatest amount of genetic variation in the subspecies and show high connectivity with martens from the OR‐CA Border, which were previously considered a distinct population (Moriarty et al. 2021). Higher genetic diversity in Northern California may be attributed to occasional gene flow with the Oregon Cascades. Northwestern California has also experienced substantially less forest change relative to the Oregon Coast Range (Strittholt et al. 2006), which may have allowed Humboldt martens to maintain higher abundance and connectivity and thereby support higher genetic diversity. Maple Creek martens to the south form a well‐supported clade that is closely related to the Northern California population, with moderate to high inbreeding and low differentiation suggesting that martens in the Maple Creek vicinity may form a small peripheral population with limited connectivity to Northern California.

Genetic structure results indicate recent gene flow between the Northern California and Southern Oregon populations, including shared coancestry, low levels of differentiation, and similar levels of genetic diversity. However, our genomic results also show signatures of small population size forces in Southern Oregon such as high within‐population relatedness, relatively high inbreeding, and forming a separate cluster and clade. These signals suggest that limited connectivity with Northern California may support genetic variation in Southern Oregon despite low abundance. Recent forest megafires (e.g., Chetco, Bar, Klondike) in Northern California and Southern Oregon may threaten gene flow in this region and signatures of gene flow should therefore be interpreted with caution given the time lag of multiple generations between loss of gene flow and accumulation of genetic signatures (Epps and Keyghobadi 2015; Gargiulo et al. 2025). Continued monitoring is critical to understanding whether gene flow persists, especially given vulnerability of a small Southern Oregon population without connectivity.

Population structure was strongest in the Central Coast Oregon populations (South Dunes, North Dunes, and North Coast), each of which deviated strongly from Humboldt martens to the south and showed high genetic differentiation from one another despite close geographic proximity (Figure 8). These patterns were consistent with extreme genetic drift and inbreeding caused by low abundances (North Dunes: *N* = 30–51, South Dunes; *N* = 21–36; Linnell et al. 2018) and geographic isolation driven by large rivers. Our results indicate that South Dunes martens are at the highest risk of extinction given the lowest genetic diversity and highest inbreeding levels (Figure 5). Conversely, we found similarly low levels of genetic diversity and high inbreeding in the North Dunes but with high variation among individuals, along with extremely small but uncertain *N*_e_ estimates. Paradoxically high genetic diversity has only been documented twice in small and isolated populations (*Ovis gmelini*, Kaeuffer et al. 2007; *Tortrix viridana*, Schroeder et al. 2010) where heterozygosity is thought to be maintained by selection for overdominance (Schou et al. 2017). Individual variation in heterozygosity may also be caused by rare immigration into the North Dunes (e.g., Jamieson 2010; Hasselgren et al. 2021) or by spatial genetic structure within the population (Black et al. 2024). We detected four females on the North Coast via scats which supports existence of nearby cryptic populations and emphasizes the need for increased survey effort throughout the Humboldt marten's historical range.

We emphasize that our results are the first in‐depth examination of population genetics in this imperiled subspecies and we recommend additional genetic sampling and demographic estimation, particularly in the Northern California/Border, North Coast Oregon, South Coast Oregon, and Applegate/Ashland regions.

### 4.6 Conservation Implications

Based on our results, all Humboldt marten populations have effective population size (*N*_e_) estimates below the recommended minimum of 50 for short‐term persistence (Franklin 1980; Frankham et al. 2014). Inbreeding and genetic drift appear strongest on the Oregon Dunes and likely increase extinction risk beyond previous estimates based on demographic stochasticity alone (Linnell et al. 2018; Frankham 2005). Maple Creek and Southern Oregon martens may face similar threats without connectivity to Northern California. Similar to recommendations for improving demographic outcomes in Humboldt martens (Slauson et al. 2019), we recommend managing habitat for population expansion and restoring or maintaining genetic connectivity following a framework such as the “Defend the Core, Grow the Core” strategy implemented for sage grouse (*Centrocercus* spp.; Naugle et al. 2024). Long‐term persistence of critically small populations may be best supported by repeated genetic rescue and habitat restoration to enable population persistence and expansion (Hedrick et al. 2019). For example, Grauer et al. (2017) showed that modest levels of immigration (i.e., 1 female immigrant per year) from a large source population supported population expansion and reduced demographic extinction probabilities in small American marten populations.

Translocations are common in *Martes* and relatives (Powell et al. 2012; Facka 2016) and our results suggest that Northern California martens would be the best source population for maintaining genetic variation within Humboldt martens. Benefits of artificial gene flow must be carefully weighed against potential negative demographic and genetic effects of removing animals from a limited source population (Furlan et al. 2020). Efforts to restore gene flow in Humboldt martens should also carefully consider genetic load of deleterious recessive variants, which accumulate in small populations via inbreeding and can threaten long‐term persistence by reducing individual fitness (Kyriazis et al. 2021; Mathur and DeWoody 2021; Dussex et al. 2023). Immigration from large populations into small, isolated populations (such as those on the Dunes) can unintentionally introduce strongly deleterious variation which small, isolated populations may have previously purged (Robinson et al. 2018; Hedrick et al. 2019; Mathur and DeWoody 2021; Dussex et al. 2023). If translocation is considered for genetic management of Humboldt martens, we strongly recommend first quantifying genetic load to assess the potential for introducing additional deleterious mutations into isolated populations (e.g., Quinn et al. 2024). We further recommend integrating estimates of genetic load with demography to understand how inbreeding in Humboldt martens impacts individual fitness and population dynamics (Kardos et al. 2025). Consequences of inbreeding depression are multi‐faceted and impacts on fitness components (e.g., survival and reproduction) rather than specific traits are more commonly observed in wild animals (Keller and Waller 2002; Hasselgren and Norén 2019). Creative approaches could be explored for measuring traits commonly impacted by inbreeding such as litter size, birth weight, body size, and susceptibility to disease and parasites (Hasselgren and Norén 2019).

## Funding

This work was supported by U.S. Fish and Wildlife Service, FWS‐ES2021002361. National Science Foundation, Graduate Research Fellowship Program.

## Conflicts of Interest

The authors declare no conflicts of interest.

## Supporting information


**Appendix S1:** eva70277‐sup‐0001‐AppendixS1.zip.

**Data S1:** eva70277‐sup‐0002‐Supinfo.docx.
**Section S1**. Humboldt marten historical range delineation.
**Section S2**. Sample sourcing details.
**Section S3**. Reduced representation sequencing library details.
**Section S4**. Parameter optimization for de novo SNP discovery.
**Section S5**. SNP filtering methods.
**Section S6**. Sex primer development and testing.
**Section S7**. GBAS panel protocols.
**Figure S1:** Humboldt marten historical range.
**Figure S2:** Total SNPs during parameter optimization.
**Figure S3:** R80 SNPs during parameter optimization.
**Figure S4:** Percent of variance explained in PC1‐2 during parameter optimization.
**Figure S5:** Sequencing depth for ddRADseq batches.
**Figure S6:** Per‐sample allele balance plots for ddRADseq.
**Figure S7:** PVCA showing batch effects.
**Figure S8:** PCoA of ddRADseq filtering versus sequencing batch.
**Figure S9:** PCoA of ddRADseq filtering versus missingness.
**Figure S10:** PCoA of ddRADseq filtering versus population.
**Figure S11:** PCoA on the high missingness ddRADseq dataset.
**Figure S12:** Rangewide ADMIXTURE models.
**Figure S13:** evalAdmix for rangewide ADMIXTURE models.
**Figure S14:** Humboldt marten ADMIXTURE models.
**Figure S15:** evalAdmix for Humboldt marten ADMIXTURE.
**Figure S16:** Heterozygosity versus sample depth.
**Figure S17:** PCoA comparison between ddRADseq and GBAS.
**Figure S18:** Comparison of individual relatedness estimates.
**Figure S19:** Comparison of genetic diversity and inbreeding estimates.
**Figure S20:** PCoA for Humboldt martens based on GBAS.
**Figure S21:** GBAS ML tree.
**Table S1:** Sample sourcing details.
**Table S2:** Reduced representation sequencing sample details.
**Table S3:** ddRADseq SNP filtering steps.
**Table S4:** *F*_ST_ Bootstrap C.I.s for ddRADseq.
**Table S5:** Details on tested sexing primers.
**Table S6:** GBAS primer sequences and concentrations.
**Table S7:** *F*_ST_ Bootstrap C.I.s for GBAS.
**Table S8:** Mitogenome nucleotide diversity estimates.

## Data Availability

Raw ddRADseq data is available on the NCBI Sequence Read Archive under PRJNA1470952. Raw GBAS panel genotypes and all code is openly available on GitHub at https://github.com/mhallerud/HumboldtMarten_PopGen_and_SNPpanel.
