# Supplementary material for: Leveraging Next‐Generation Tools for Genetic Assessment and Demographic Monitoring in Threatened and Elusive Humboldt Martens
Source: Evol Appl. 2026 Jul 9;19(7):e70277. doi: 10.1111/eva.70277 (PMC13351330; doi:10.1111/eva.70277)
Supplement: Supplementary file 2 — Data S1: eva70277‐sup‐0002‐Supinfo.docx. Section S1. Humboldt marten historical range delineation. Section S2. Sample sourcing details. Section S3. Reduced representation sequencing library details. Section S4. Parameter optimization for de novo SNP discovery. Section S5. SNP filtering methods. Section S6. Sex primer development and testing. Section S7. GBAS panel protocols. Figure S1: Humboldt marten historical range. Figure S2: Total SNPs during parameter optimization. Figure S3: R80 SNPs during parameter optimization. Figure S4: Percent of variance explained in PC1‐2 during parameter optimization. Figure S5: Sequencing depth for ddRADseq batches. Figure S6: Per‐sample allele balance plots for ddRADseq. Figure S7: PVCA showing batch effects. Figure S8: PCoA of ddRADseq filtering versus sequencing batch. Figure S9: PCoA of ddRADseq filtering versus missingness. Figure S10: PCoA of ddRADseq filtering versus population. Figure S11: PCoA on the high missingness ddRADseq dataset. Figure S12: Rangewide ADMIXTURE models. Figure S13: evalAdmix for rangewide ADMIXTURE models. Figure S14: Humboldt marten ADMIXTURE models. Figure S15: evalAdmix for Humboldt marten ADMIXTURE. Figure S16: Heterozygosity versus sample depth. Figure S17: PCoA comparison between ddRADseq and GBAS. Figure S18: Comparison of individual relatedness estimates. Figure S19: Comparison of genetic diversity and inbreeding estimates. Figure S20: PCoA for Humboldt martens based on GBAS. Figure S21: GBAS ML tree. Table S1: Sample sourcing details. Table S2: Reduced representation sequencing sample details. Table S3: ddRADseq SNP filtering steps. Table S4: F ST Bootstrap C.I.s for ddRADseq. Table S5: Details on tested sexing primers. Table S6: GBAS primer sequences and concentrations. Table S7: F ST Bootstrap C.I.s for GBAS. Table S8: Mitogenome nucleotide diversity estimates. [file EVA-19-e70277-s001.docx]

# **Supplemental Information for:**

**Leveraging next-generation tools for genetic assessment and demographic monitoring in threatened and elusive Humboldt martens**

# **Table of Contents:**

| **Section S1. Humboldt marten historical range delineation** | Page 3 |
| --- | --- |
| **Section S2. Sample sourcing details** | Page 3 |
| **Section S3. Reduced representation sequencing library details** | Page 4 |
| **Section S4. Parameter optimization for de novo SNP discovery** | Page 5 |
| **Section S5. SNP filtering methods** | Page 6 |
| **Section S6. Sex primer development and testing** | Page 6 |
| **Section S7. GBAS panel protocols** | Page 7 |
| **Figure S1. Humboldt marten historical range** | Page 9 |
| **Figure S2. Total SNPs during parameter optimization** | Page 10 |
| **Figure S3. R80 SNPs during parameter optimization** | Page 11 |
| **Figure S4. Percent of variance explained in PC1-2 during**  **parameter optimization** | Page 12 |
| **Figure S5. Sequencing depth for ddRADseq batches** | Page 13 |
| **Figure S6. Per-sample allele balance plots for ddRADseq** | Page 14 |
| **Figure S7. PVCA showing batch effects** | Page 20 |
| **Figure S8. PCoA of ddRADseq filtering vs. sequencing batch** | Page 21 |
| **Figure S9. PCoA of ddRADseq filtering vs. missingness** | Page 22 |
| **Figure S10. PCoA of ddRADseq filtering vs. population** | Page 23 |
| **Figure S11. PCoA on the high missingness ddRADseq dataset** | Page 24 |
| **Figure S12. Rangewide ADMIXTURE models** | Page 25 |
| **Figure S13. evalAdmix for rangewide ADMIXTURE models** | Page 26 |
| **Figure S14. Humboldt marten ADMIXTURE models** | Page 28 |
| **Figure S15. evalAdmix for Humboldt marten ADMIXTURE** | Page 29 |
| **Figure S16. Heterozygosity vs. sample depth** | Page 30 |
| **Figure S17. PCoA comparison between ddRADseq and GBAS** | Page 31 |
| **Figure S18. Comparison of individual relatedness estimates** | Page 32 |
| **Figure S19. Comparison of genetic diversity and inbreeding**  **estimates** | Page 33 |
| **Figure S20. PCoA for Humboldt martens based on GBAS** | Page 34 |
| **Figure S21. GBAS ML tree** | Page 35 |
| **Table S1. Sample sourcing details** | Page 36 |
| **Table S2. Reduced representation sequencing sample details** | Page 37 |
| **Table S3. ddRADseq SNP filtering steps** | Page 38 |
| **Table S4. F_ST_ Bootstrap C.I.s for ddRADseq** | Page 39 |
| **Table S5. Details on tested sexing primers** | Page 40 |
| **Table S6. GBAS primer sequences and concentrations** | Page 41 |
| **Table S7. F_ST_ Bootstrap C.I.s for GBAS** | Page 46 |
| **Table S8. Mitogenome nucleotide diversity estimates** | Page 47 |
| **Literature Cited** | Page 48 |

#

# Section S1. Humboldt marten historical range delineation

Coastal marten historical range was delineated by comparing all known historical marten detections with EPA ecoregions and General Land Office historical vegetation data. The starting point was the U.S. EPA Level III ecoregions of the Coast Range and Klamath Mountains south of the Columbia River. The EPA level IV ecoregions of the Willamette Valley Foothills, North Coast Range Eastern Slopes, and Sonoma-Mendocino Mixed Forest were added based on historical marten detections and historic vegetation composition. The Klamath and Trinity Rivers were set as the boundary between genetically distinct Marble Mountains martens to the east and coastal martens to the west based on evidence in Schwartz and others (2020). The updated coastal marten historical range map covers 64,396 square kilometers and extends from Santa Rosa, California to the Columbia River, Oregon.

Possible and potential natural hybrid zones between coastal martens (*M. c. humboltensis*) and martens in the Oregon Cascades (*M. c. caurina* based on Hall 1981) or Lassen/Shasta National Forest areas (*M. c. sierrae* based on Hall 1981) were also delineated. There is a possible existing hybrid zone between coastal martens and Cascades martens in the inland Siskiyous between Grants Pass and Roseburg. Possible historical hybrid zones that may have acted as filters between coastal and Cascades martens include the Portland/Vancouver Basin, the southernmost extent of the Willamette Valley Foothills south of present-day Eugene, the Umpqua Interior Foothills surrounding present-day Roseburg, and areas surrounding the present-day Cascade-Siskiyou National Monument. Although there is little evidence of hybridization between coastal and Lassen/Shasta martens, the most likely route would be on the Six Rivers National Forest by crossing the South Fork Trinity or eastern reaches of the Klamath River. Areas included in hybrid zones capture the majority of uncertainty about the eastern edge of coastal marten range.

See Figure S1 and Appendix 1 for a shapefile of the historical range map.

# Section S2. Sample sourcing details

We sourced 91 high-quality Pacific marten samples from archived DNA extracts (including many previously analyzed in Schwartz et al. 2020), roadkill, and ear biopsy punches collected during recent live-trapping efforts under USFWS Recovery Permit #ESPER0011953-0. We also collected 49 fresh scats belonging to 30 unique individuals during live-trapping of Humboldt martens in Northern California and Southern Oregon and montane martens in Lassen National Forest. Identifying information from 15 fresh Humboldt marten scats from known individuals was redacted prior to shipment to the Levi Lab in order to anonymously test the GBAS panel.

We collected most noninvasive samples between 2014 and 2021 using USFWS-permitted scat detection dog surveys by Rogue Detection Teams. The Karuk Tribe shared additional hair samples collected during hair snare surveys in 2023-2024. Additional scat samples were collected opportunistically during camera-trapping led by CE in 2016-2017 and MH in 2021.

We stored ear biopsy punches in sterile silica desiccant or in a -20 C freezer in 95% ethanol and DNA extracts in -80C freezer; sample storage otherwise varied widely given opportunistic sourcing and previously analyzed DNA extracts often experienced multiple freeze-thaw cycles. Sample source information can be found in Table S1.

We extracted DNA from all new samples using DNeasy Blood and Tissue kits (Qiagen). We followed standard protocols for tissue and blood samples and a slightly modified bead-beating protocol (Roffler et al. 2021) for scat and hair samples, plus the addition of 50 µl of 1 µM DTT to lysis buffer for hair samples. DNA was extracted in separate batches for high-quality and noninvasive samples in a PCR-free lab, and we included an extraction blank within each batch.

# Section S3. Reduced representation sequencing library details

Eighty samples (50 Humboldt martens and 30 ‘montane’ martens) had sufficient DNA quantity (>150 ng) based on Qubit dsDNA quantitation (Thermo Scientific) for genomic sequencing. We submitted samples in three separate batches for ddRADseq library preparation and sequencing, with each batch normalized to 180 ng / 20 µl or 200 ng / 20 µl. We included 19 total technical replicates within and across libraries to estimate genotyping accuracy and batch effects (Bresadola et al. 2020). We visualized DNA fragment size for a subset of samples to ensure genomic DNA quality via standard gel electrophoresis and automated electrophoresis on a 2100 Bioanalyzer system (Agilent) visualized using TapeStation software v4.1 (Agilent).

We submitted normalized plates to Oregon State University’s Center for Quantitative Life Sciences (CQLS) for double-digest RADseq (ddRADseq) with enzymes PstI and MspI followiing the protocol by Poland et al. (2012). Briefly, DNA extracts were normalized to 20 ng/μL and digested with enzymes PstI (CTGCAG) and MspI (CCGG). The digested product was then size-selected for 175-600 bp fragments using BluePippin (Sage Science, Beverly, MA, USA). Adapters with unique barcodes were ligated followed by pooling and purification using QIAquick PCR Purification Kit (Qiagen). The ligated samples were PCR-amplified for 15 cycles consisting of 98°C (10 min), 68°C (30 s), 72°C (30 s), 72°C (5 min) to create the final library. Fragment size of each library was verified via Bioanalyzer trace (Agilent). Finally, each library was sequenced on an Illumina NextSeq 2000 P2 lane for 150-bp paired end reads.

We used FastQC (Andrews 2010) to check quality of raw sequencing data. Plate 2 had slightly higher per-sample read counts (Figure S2A) and sequencing depths (Figure S2B), while plate 1 which included failed samples with lower starting input DNA had the lowest. Samples with <2 million reads had substantially higher levels of missing SNPs; most of these samples belonged to plate 1 (Figure S2C). Allele balance plots (Figure S3) suggest that data is reliable despite lower read counts for these individuals. Samples that passed filtering and were included in ddRADseq analyses are in Table S2.

# Section S4. Parameter optimization for *de novo* SNP discovery

We quality-checked raw reads using FastQC (Andrews 2010) and demultiplexed using the process_radtags module in Stacks v2 (Catchen et al. 2013). We used the *de novo* SNP discovery pipeline in Stacks v2 (Catchen et al. 2013) to call SNPs. We optimized *de novo* Stacks parameters using 5 random samples from 5 geographically-recognized populations and varied parameters (in order) for m (minimum depth per locus per sample) from 2-6, M (number of mismatches allowed when merging reads within a sample) from 1-8, and n (number of mismatches allowed when merging stacks across individuals) from M-1 to M+1. For parameter optimization, we considered the number of SNPs, number of loci present in >80% of samples (“R80”), proportion of heterozygous individuals, cumulative variation explained in the first 5 principal components, the correlation between pairwise missingness and genetic dissimilarity, and the percent of inferred paralogs (Mastretta-Yanes et al. 2015; McCartney-Melstad et al. 2015; Paris et al. 2017; Rochette and Catchen 2017). Paralogs were inferred via excess heterozygosity (>60%), excess depth (>200), and unbalanced allele depth ratios (AB>5 or AB<-5). We identified optimal parameters as m=4, M=4, and n=5 primarily based on maximizing the total number of SNPs (Figure S4), maximizing the number of R80 loci (Figure S5), and maximizing the percent of variation explained in the first 5 principal components (Figure S6).

Given concerns about over-merging loci due to relatively high M and n parameters, we compared the proportion of Stacks merged at the respective steps with those merged at more conservative values and found nominal differences. Our more conservative values allowed a maximum difference of 2 SNPs at a given locus both within a sample (M) and between samples (n). During initial stack assembly within a sample in ustacks, the average percent of stacks lost to merging was 7.5% with M=4 and 5.9% with M=2. Similarly, when stacks were merged to create loci across samples, ~2-5% additional stacks per sample were merged at n=5 compared to n=2.

# Section S5. SNP filtering methods

We strictly filtered SNPs following recommendations in O’Leary et al. (2018) using a combination of vcftools v0.1.16 (Danecek et al. 2011), plink v1.9 (Purcell et al. 2007),

bcftools 1.21 (Danecek et al. 2021), seqkit2 (Shen et al. 2024), and custom bash scripts. Briefly, SNPs were filtered for sequencing error (depth≥5, minor allele count ≥2, mean depth ≥8, quality score >20), iteratively for per-locus and per-sample missingness (final missingness <30% per locus and <50% per sample), allele dropout (standard deviation of depth <99^th^ percentile), allelic balance (0.2—0.8), Hardy-Weinberg equilibrium (Benjamini-Hochberg corrected Hardy-Weinberg p-values >0.05 in most populations; Pearman et al. 2022), paralogs (mean depth < mean(mean depths) + 2 * std dev(mean depths) = 36; McKinney et al. 2017), and allele dropout (heterozygosity <0.60). This initial filtered dataset comprised the ‘high missingness dataset’ and was further filtered to create 1) an ‘LD-pruned dataset’ and 2) a ‘low missingness dataset’. For the low missingness dataset, SNPs with >5% missingness and samples with >10% missingness were removed. Linkage disequilibrium pruning used a sliding window approach with a window size of 50 SNPs and step size of 5 SNPs where one SNP from each pair of SNPs with high Pearson’s correlation (r>0.60) in the window is removed (plink --indep-pairwise 50 5 0.60).

Functions used, thresholds, and remaining SNPs and samples at each filtering step are found in Table S3. The effects of major filtering steps on structure caused by batch effects, missingness, and geographic regions are shown respectively in Figures S7−S9.

# Section S6. Sex primer development and testing

We tested 3 previously published primer pairs and designed 3 new primer pairs with 2 targeting the sex-determining region (SRY) in *Martes americana* [GenBank Accession: GU380297.1​​; Campbell et al. 2010] and 1 targeting the zinc finger X/Y protein (ZFX/Y) in *Martes martes* [GenBank Accession: FN421125.1; Mullins et al. 2009]). We designed primers using Primer3 software (Untergasser et al. 2012) with the same specifications as used for multiplex primer design (e.g., 60℃ annealing temperature, 70-130 bp), then used PRIMER-BLAST (Ye et al. 2012) to check for specificity against all mammals (Class: Mammalia) and all birds (Class: Aves). See Table S4 for primer sequences.

We initially tested the MMY/MMX primers tested on 2 male tissues and 3 male scats. After these failed to amplify, we tested ZFXY w542/3, SRY MAAM-1, SRY MAAM-4, and ZFY MAMA-1 on DNA extracts from 1 male tissue, 2 female tissues, 2 male scats, and 1 female scat (with sex of scats known by using trapping-collected scats). All tissue DNA extracts were diluted 1:10 for tests to conserve DNA.

For each test, we ran singleplex PCR with 10 ul AmpliTag Gold, 2.5 ul of 1μM FWD primer, 2.5 ul of 1 μM REV primer, 2 μl DNA for scats or 1 μl DNA for tissue, and 3 μl H2O for scats or 4 μl for tissue to bring the final volume to 20 μl. PCR cycling conditions followed the multiplex PCR conditions described in the next section. We then visualized PCR products on a 1% agarose gel using a lambda HindIII DNA ladder (ThermoFisher). Primer pairs that showed successful amplification (i.e., SRY MAAM-1 and SRY MAAM-4) in initial tests were then added to the optimized multiplex primer mix at 0.2 μM concentration and included during initial sample identification tests which included 17 male and 8 female marten scats and 5 diluted male marten tissue samples. Identification tests followed library prep details below and were sequenced on an Illumina NextSeq 2000 at OSU’s Center for Quantitative Life Sciences. Sex primer concentrations within the primer mix were adjusted based on read counts from this initial test.

# Section S7. GBAS panel protocols

We ordered adapter-ligated (FWD: 5’-TCGTCGGCAGCGTCAGATGTGTATAAGAGACAG-3’, REV: 5’-GTCTCGTGGGCTCGGAGATGTGTATAAGAGACAG-3’) primers at 100 or 200 μM concentration with standard desalting from Integrated DNA Technologies. We then created separate FWD and REV primer mixes at 5X concentration (see Table S5 for primer sequences and volumes included in primer mix). We first amplified DNA in an initial multiplex PCR reaction with 4 μl 5X FWD primer mix, 4 μl 5X REV primer mix, 10 μl AmpliTaq Gold (ThermoFisher), and 2 μl DNA template. The temperature schedule for multiplex PCR was an initial denaturation for 12 minutes at 95℃, 45 cycles with a 30 sec denaturation phase at 95℃ – 30 sec annealing phase at 60℃ – 30 sec extension phase at 72℃, and a final 7 minute extension phase at 72℃.

Following multiplex PCR, products were purified via a full-plate 1.8x MagBead cleanup (Omega) and then used as the template in a second PCR where Illumina Unique Dual Indexes (UDI) were added. The indexing PCR setup included 12.5 μl of Q5 high-fidelity polymerase (New England Biosciences), 2 μl of 10 μM UDI plate (with FWD and REV pooled, equivalent to 1 μl each of FWD and REV), and 10.5 μl of the multiplex PCR product. The indexing PCR temperature schedule was 30 sec at 98℃, 8 cycles of 10 sec at 98℃ – 30 sec at 55℃ – 30 sec at 65℃, and 5 min at 65℃. Lastly, products from the indexing PCR were fluorescently quantified using PicoGreen dsDNA quantitation kit (ThermoFisher), normalized, and pooled. Each pooled library went through another 1.8x MagBead purification before quantification and normalization with other libraries prior to submission for 150 bp paired end sequencing on an Illumina NextSeq 2000 at Oregon State University’s Center for Quantitate Life Sciences.

**Figure S1.** Historical range development for coastal martens. EPA Level IV ecoregions are shown at left with the EPA level III ecoregions for Klamath Mountains and Coast Range outlined in black. The updated historical range map (center) sets the boundary between coastal martens and montane martens as the Klamath River and shows potential coastal marten hybrid zones, pre-1980 coastal marten detections, and major rivers that may serve as barriers to martens. The Willamette Valley Foothills immediately south of Eugene likely also served as a historical hybrid zone between coastal and Cascades martens. Single-hashed hybrid zones represent areas that likely served as historical filters to marten movement. The current known distribution (right) shows recent marten detections (white for post-2010 and gray for pre-2010) overlaid on current broad landcover types and major road networks (orange).
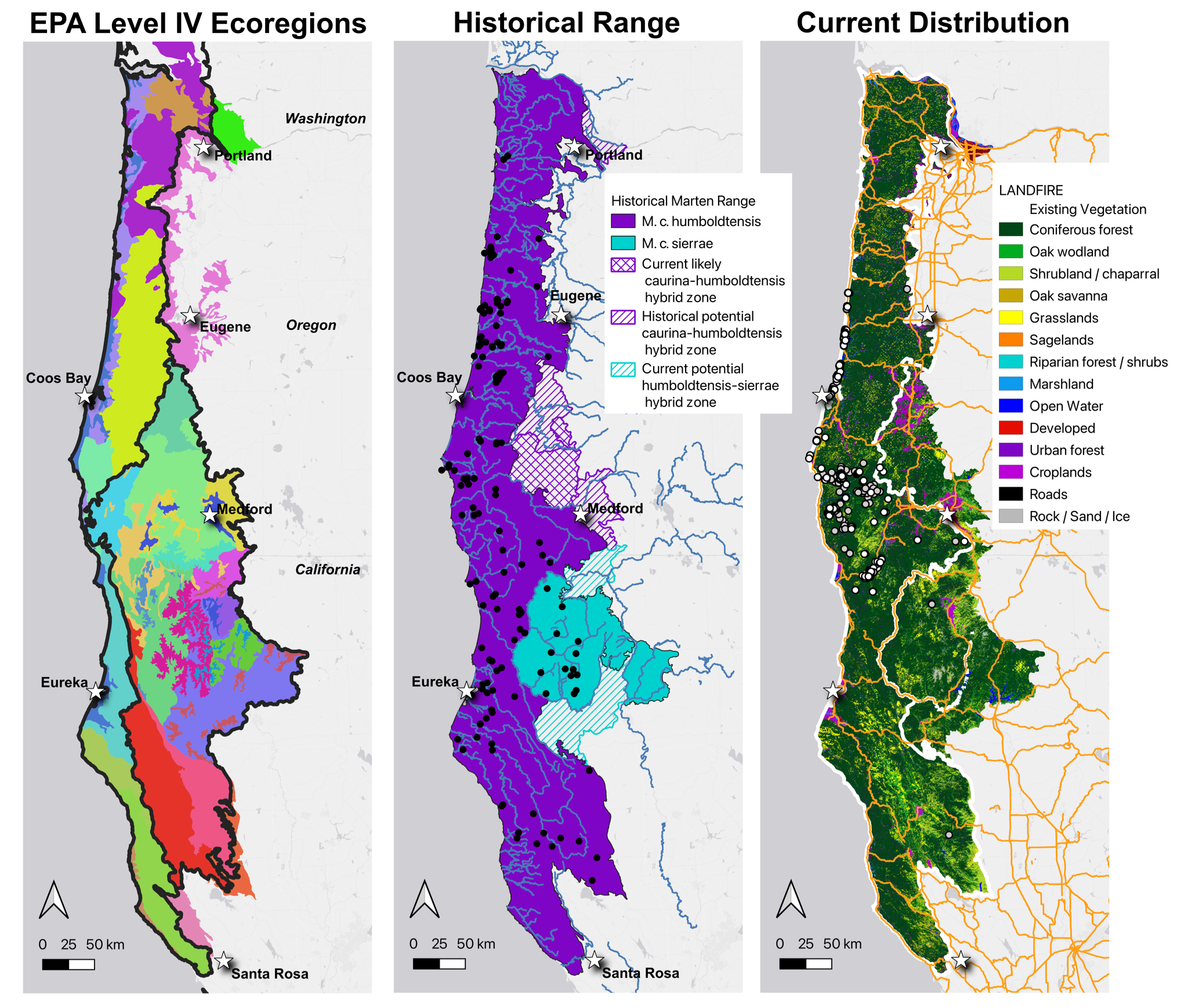


###### **Figure S2.** Total SNPs discovered at various Stacks parameter values with colors representing different populations. Note that M=4 (# mismatches allowed within a sample) and n=5 (# mismatches allowed between individuals) maximized the number of SNPs discovered.


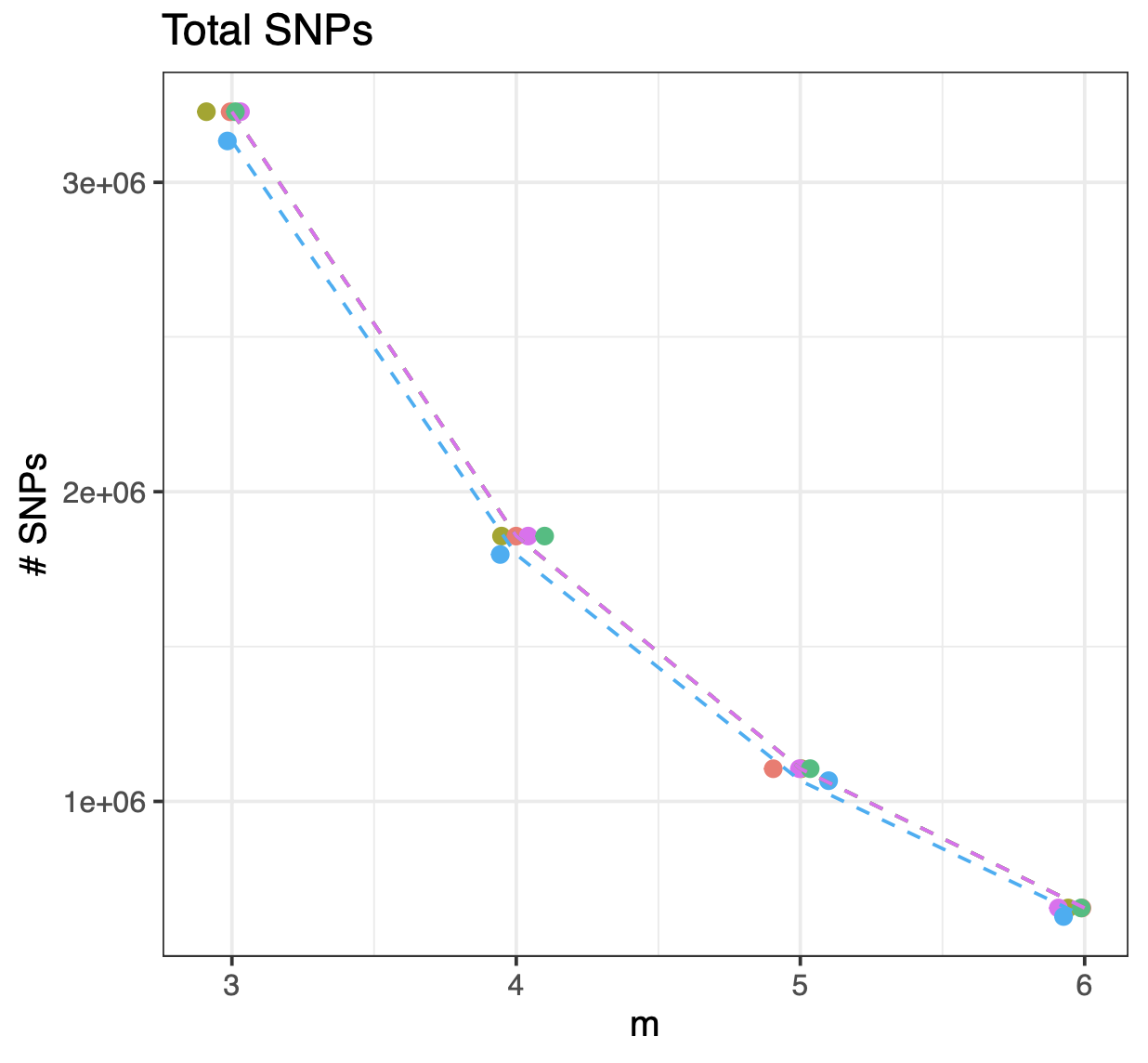

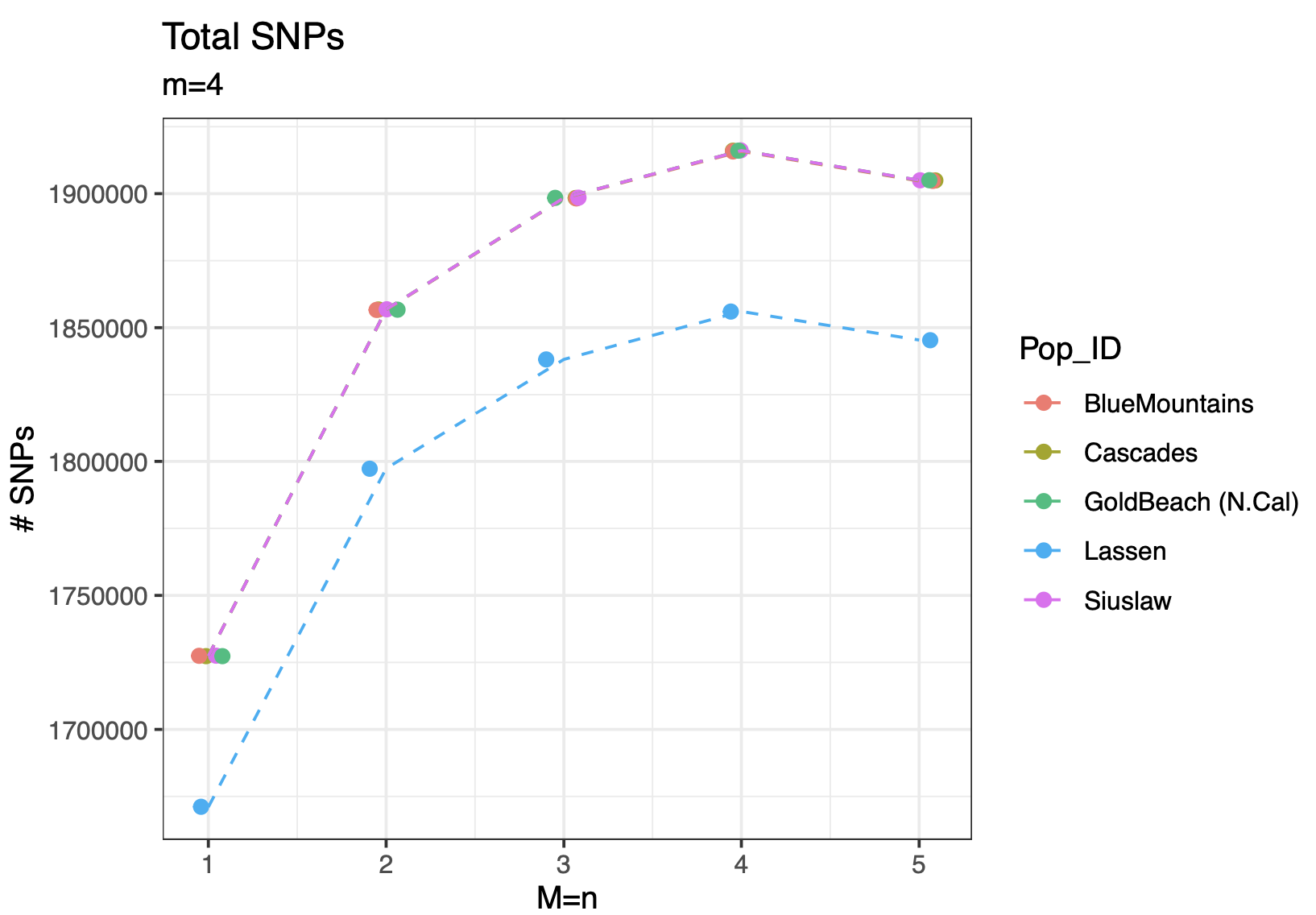

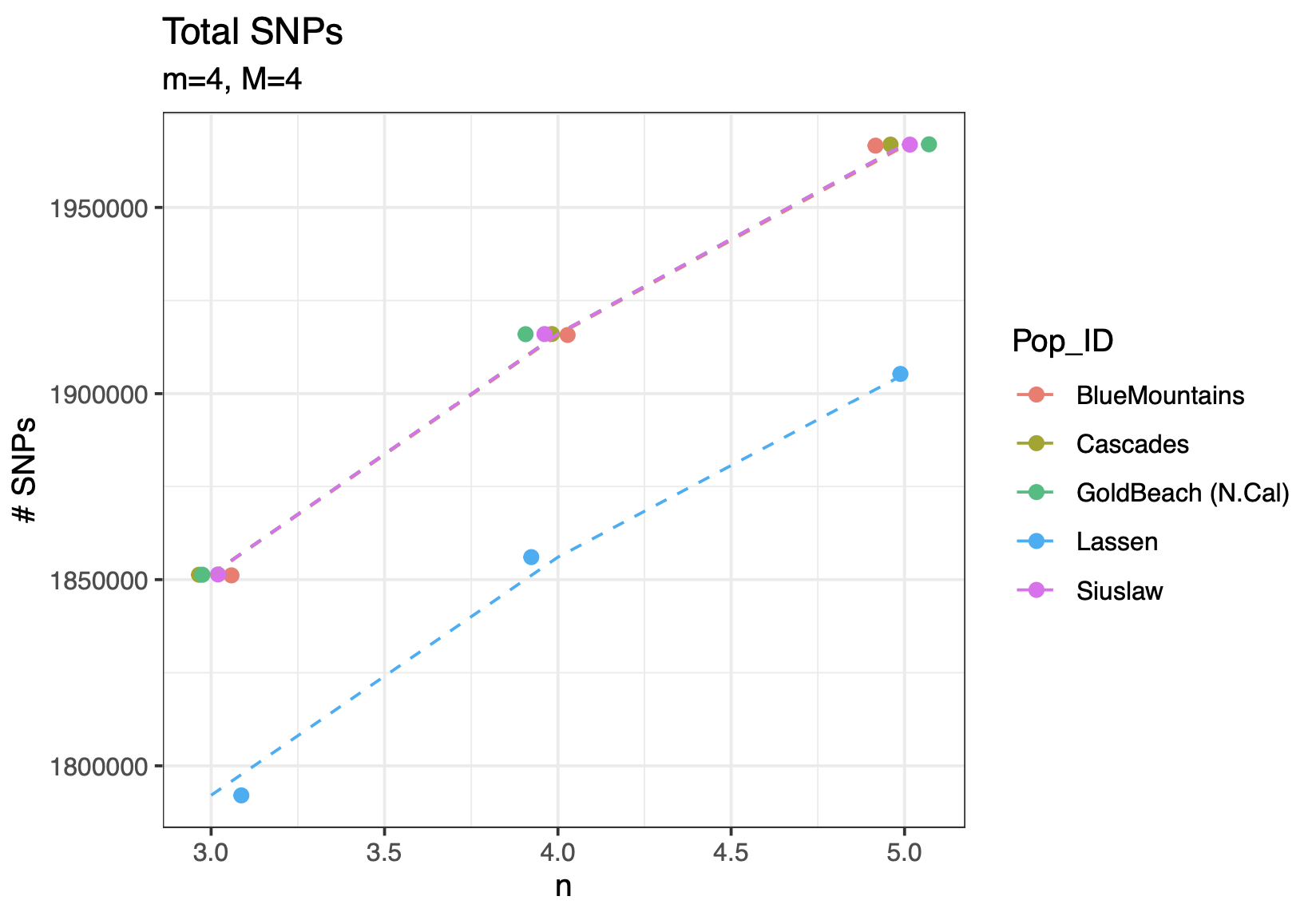


###### **Figure S3.** Number of loci discovered across ≥80% of individuals at various Stacks parameter values with colors representing different populations. Note that higher M and n values maximized the number of R80 loci.


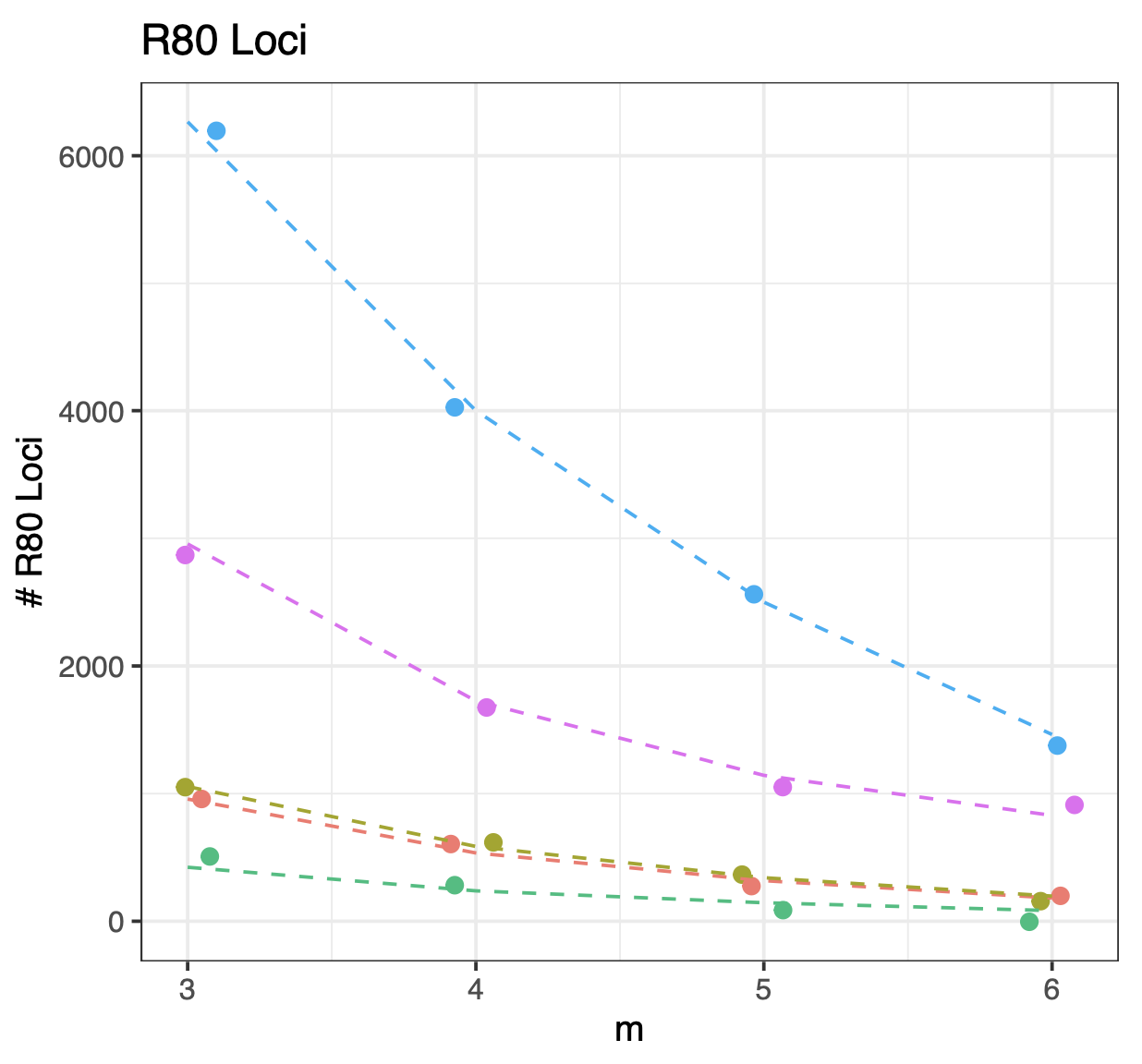

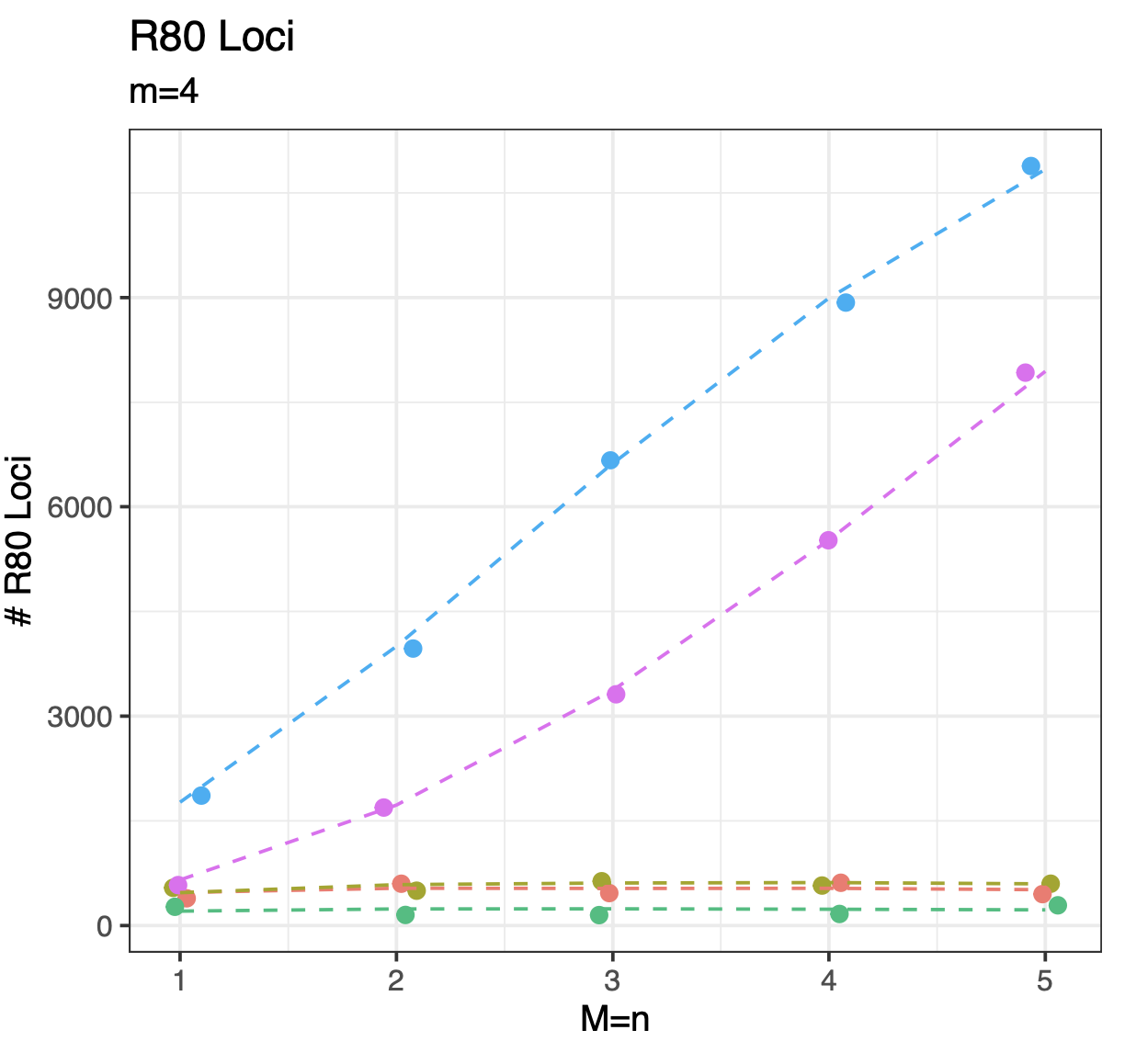


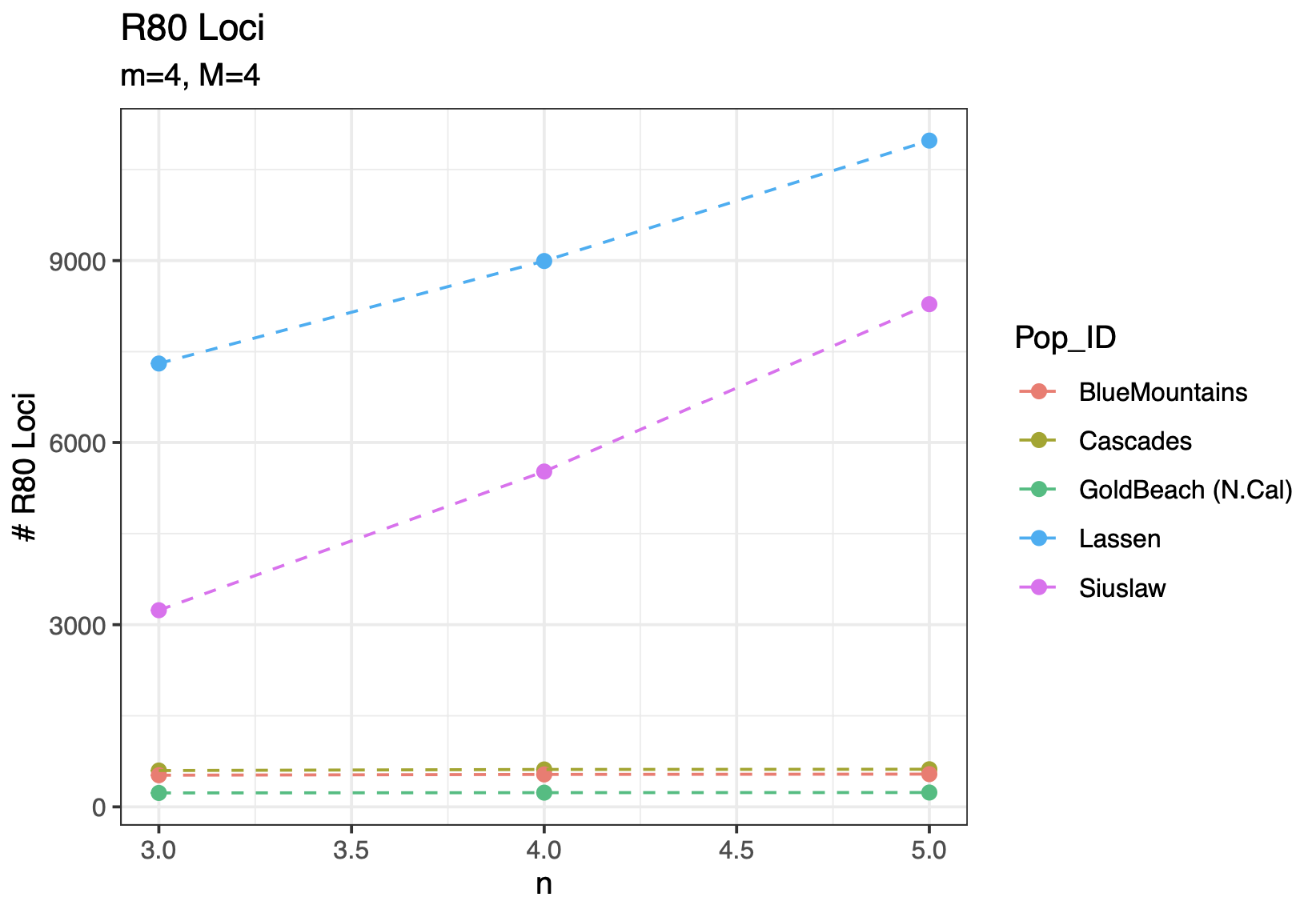


###### **Figure S4.** Percent of cumulative variance occurring in the first 5 principal components, with colors representing minimum per sample depth (m), the x axis representing maximum number of differences within a sample (M), and shapes representing the maximum number of mismatches between samples (n). Note that m=4, M=4, n=5 maximized information content.


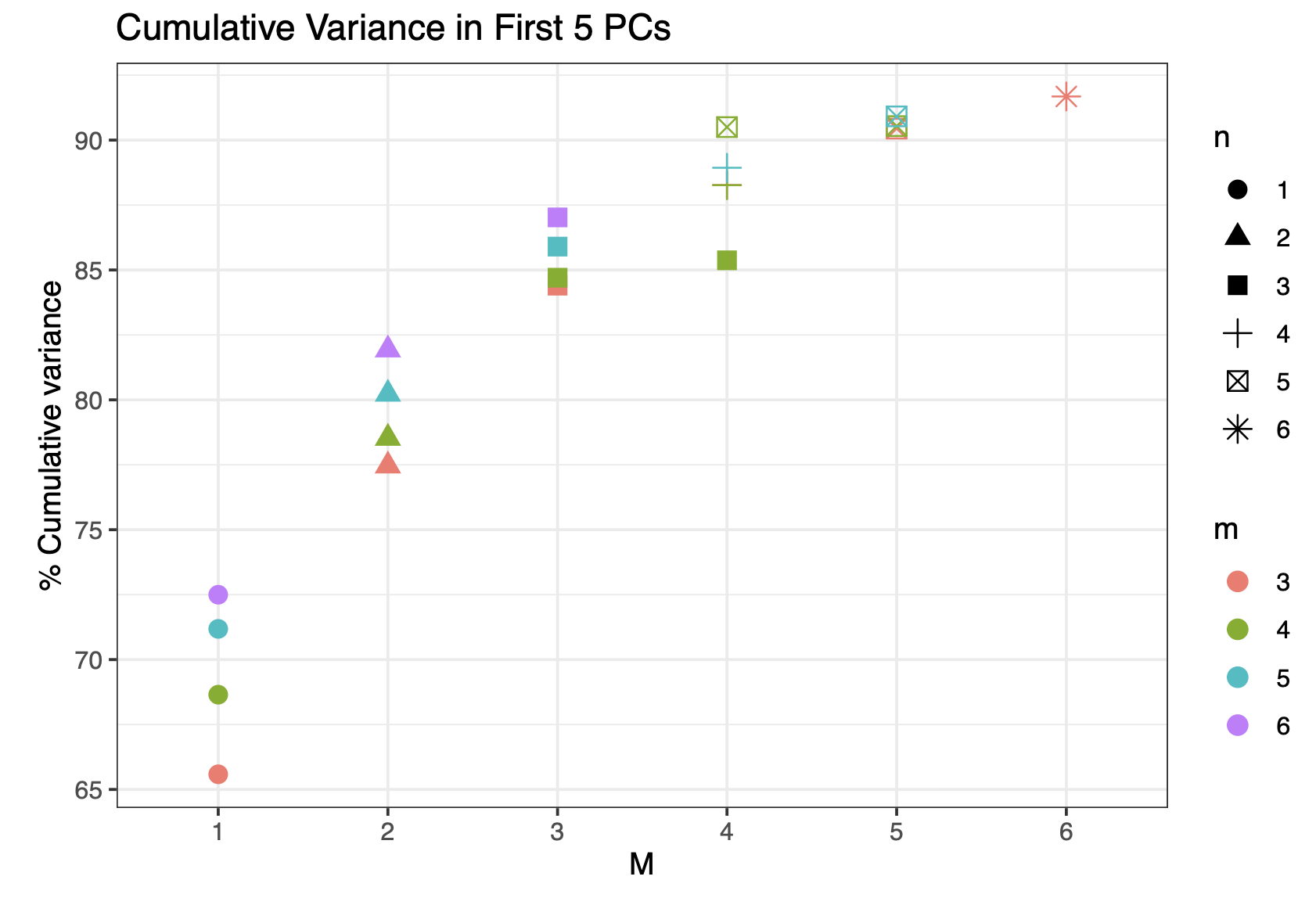


##

**Figure S5.** A) Distributions of raw sequencing reads per sample for each ddRADseq plate. B) Mean effective coverage depths per sample for each ddRADseq library/plate (unfiltered output). C) Raw sequencing read counts (x-axis) vs. proportion of SNPs with missing data (y-axis) in the high missingness dataset, colored by library. Labeled samples were removed for the low missingness dataset at a threshold of 0.1, most of these derived from plate 1. ‘Trinidad’ prefix is equivalent to ‘MapleCrk’.

**A B**


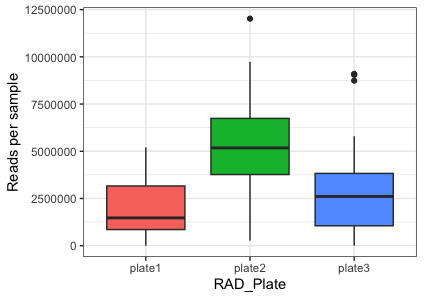

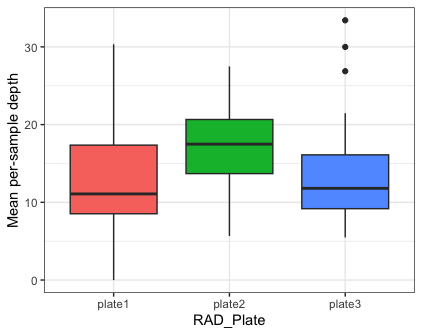


**C**


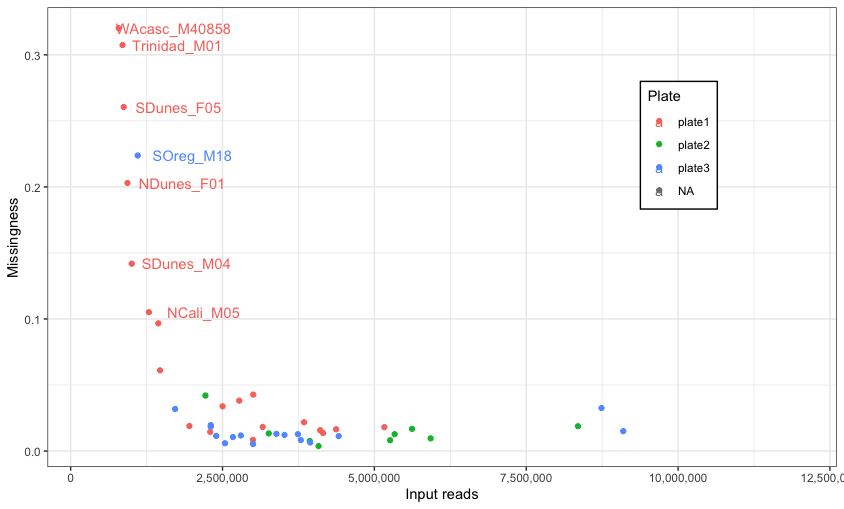


**Figure S6.A** Per-sample allele balance plots for heterozygous SNPs from raw Stacks output with read counts for A1 on the x-axis and for A2 on the y-axis. Darker colors representing higher SNP density at a given A1/A2 value, with different color palettes used for each library (purple/orange=plate 1,green/yellow=plate 2, blue/green=plate 3). Lines denote allele balances of 1:1 (solid), 0.8:1 (dashed), and 0.6:1 (dotted). Allele balances forming multiple clusters indicate potential sample contamination. Plots limited to 50 reads per axis for clarity. . ‘Trinidad’ prefix is equivalent to ‘MapleCrk’.


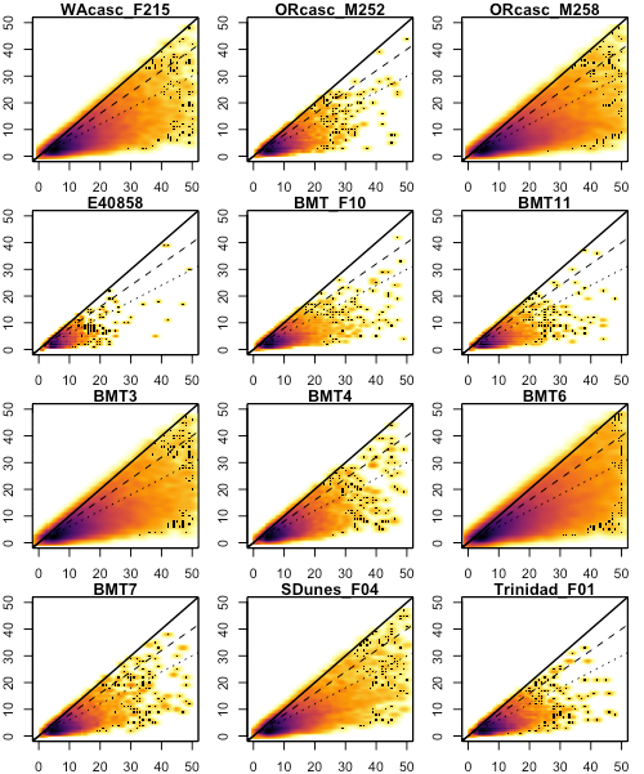


###### **Figure S6.B** Per-sample allele balance plots for heterozygous SNPs from raw Stacks output with read counts for A1 on the x-axis and for A2 on the y-axis. Darker colors representing higher SNP density at a given A1/A2 value, with different color palettes used for each library (purple/orange=plate 1, blue/green=plate 2, green/yellow=plate 3). Lines denote allele balances of 1:1 (solid), 0.8:1 (dashed), and 0.6:1 (dotted). Allele balances forming multiple clusters indicate potential sample contamination. Plots limited to 50 reads per axis for clarity.


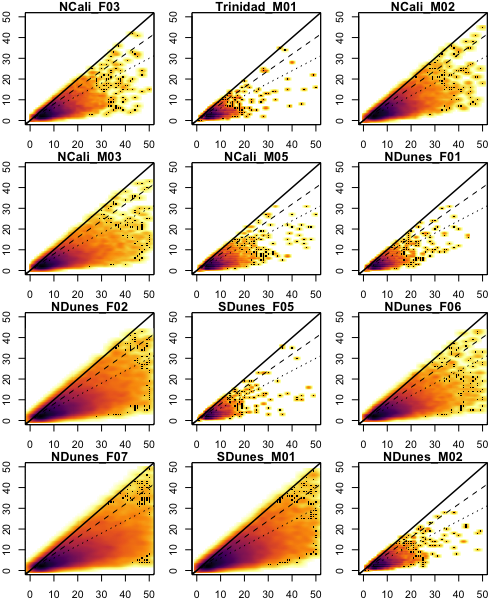


###### **Figure S6.C** Per-sample allele balance plots for heterozygous SNPs from raw Stacks output with read counts for A1 on the x-axis and for A2 on the y-axis. Darker colors representing higher SNP density at a given A1/A2 value, with different color palettes used for each library (purple/orange=plate 1, blue/green=plate 2, green/yellow=plate 3). Lines denote allele balances of 1:1 (solid), 0.8:1 (dashed), and 0.6:1 (dotted). Allele balances forming multiple clusters indicate potential sample contamination. Plots limited to 50 reads per axis for clarity.


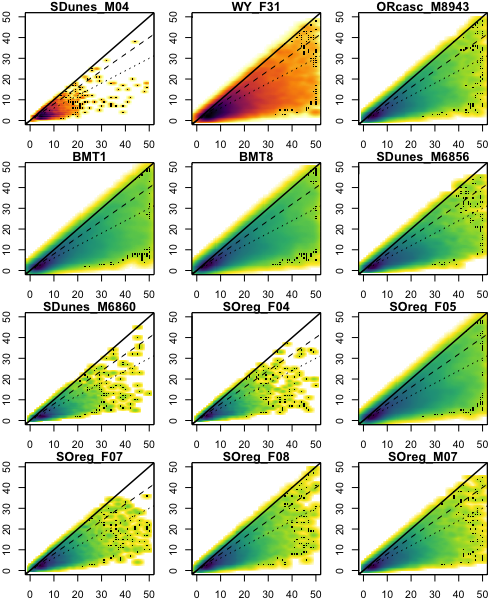


###### **Figure S6.D** Per-sample allele balance plots for heterozygous SNPs from raw Stacks output with read counts for A1 on the x-axis and for A2 on the y-axis. Darker colors representing higher SNP density at a given A1/A2 value, with different color palettes used for each library (purple/orange=plate 1, blue/green=plate 2, green/yellow=plate 3). Lines denote allele balances of 1:1 (solid), 0.8:1 (dashed), and 0.6:1 (dotted). Allele balances forming multiple clusters indicate potential sample contamination. Plots limited to 50 reads per axis for clarity. ‘Trinidad’ prefix is equivalent to ‘MapleCrk’.


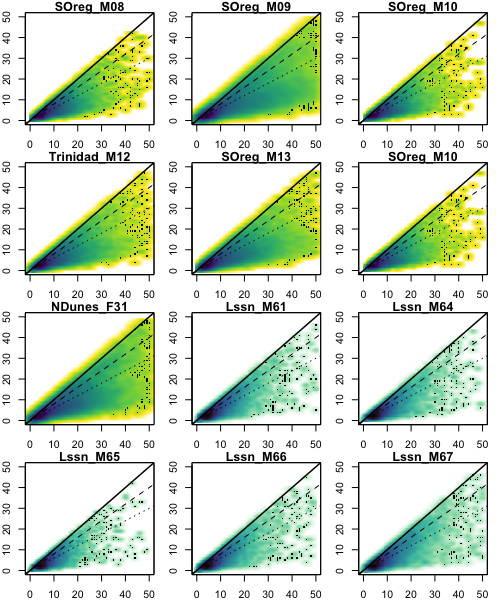


###### **Figure S6.E** Per-sample allele balance plots for heterozygous SNPs from raw Stacks output with read counts for A1 on the x-axis and for A2 on the y-axis. Darker colors representing higher SNP density at a given A1/A2 value, with different color palettes used for each library (purple/orange=plate 1, blue/green=plate 2, green/yellow=plate 3). Lines denote allele balances of 1:1 (solid), 0.8:1 (dashed), and 0.6:1 (dotted). Allele balances forming multiple clusters indicate potential sample contamination. Plots limited to 50 reads per axis for clarity. . ‘Trinidad’ prefix is equivalent to ‘MapleCrk’.


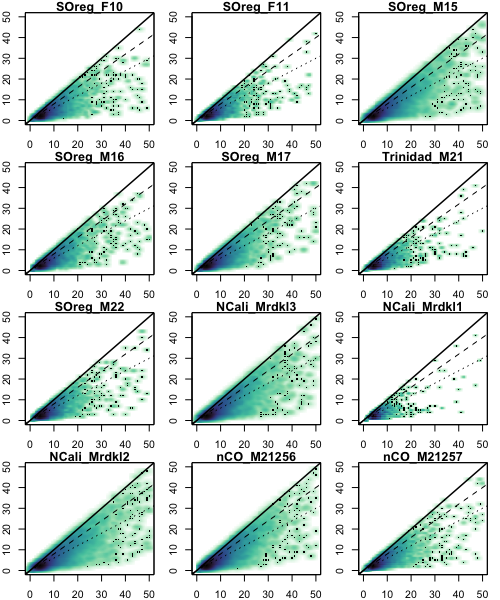


###### **Figure S6.F** Per-sample allele balance plots for heterozygous SNPs from raw Stacks output with read counts for A1 on the x-axis and for A2 on the y-axis. Darker colors representing higher SNP density at a given A1/A2 value, with different color palettes used for each library (purple/orange=plate 1, blue/green=plate 2, green/yellow=plate 3). Lines denote allele balances of 1:1 (solid), 0.8:1 (dashed), and 0.6:1 (dotted). Allele balances forming multiple clusters indicate potential sample contamination. Plots limited to 50 reads per axis for clarity.


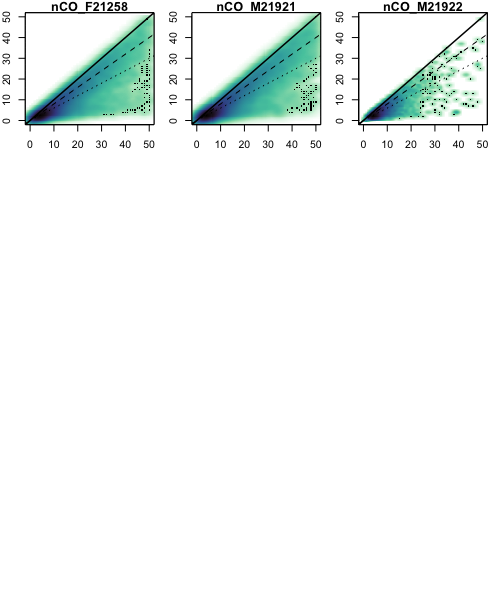


###### **Figure S7.** Results from principal variance components analysis on minimally filtered (left) and high-missingness (right) datasets. The first 10 PC axes explained 93% of variation in the minimally filtered data, of which batch effects explained 14.8%; 87% of variation was explained in the high-missingness dataset of which 13.3% was explained by batch effects. The plots show the cumulative weighted proportion of variation explained by batch effects and residual model variation within the PCA of each dataset, then the proportion of variance explained in each PC axis by batch and residual effects based on random effects models.


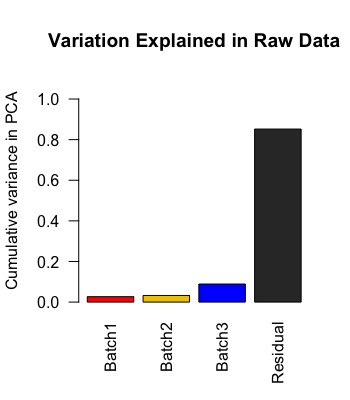

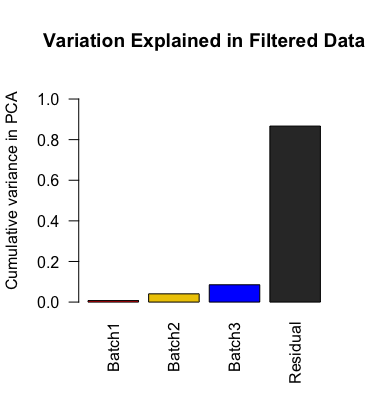


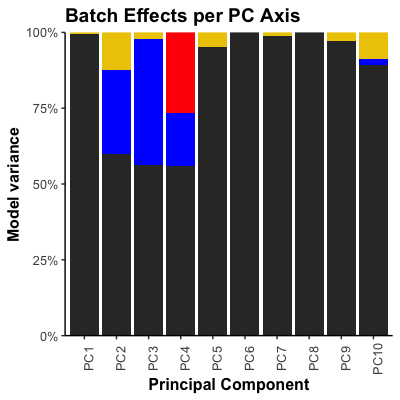

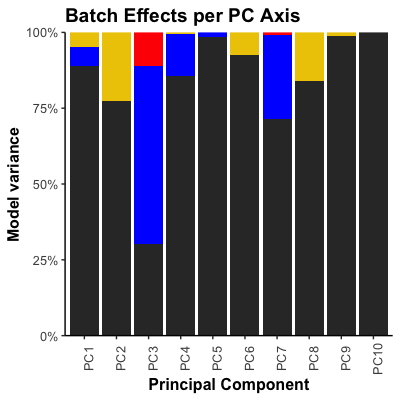


###### **Figure S8.** PCoA of ddRADseq at major filtering steps including A) raw Stacks output, B) minimum quality filtering, C) outlier removal with outliers labeled in A and B, D) SNP missingness <30%, E) sample missingness <50%, F) biased SNPs removed forming the high missingness dataset used in analyses, G) low missingness dataset used in analyses, H) LD-pruned dataset used in analyses, and I) low missingness dataset with LD-pruning. Sample points are colored by ddRADseq library to visualize batch effects.Plots F-I suggest that observed population structure is not driven substantially by batch effects (note that populations were not randomized across libraries, see Figure S9). Note that technical replicates have not been removed from these visualizations.
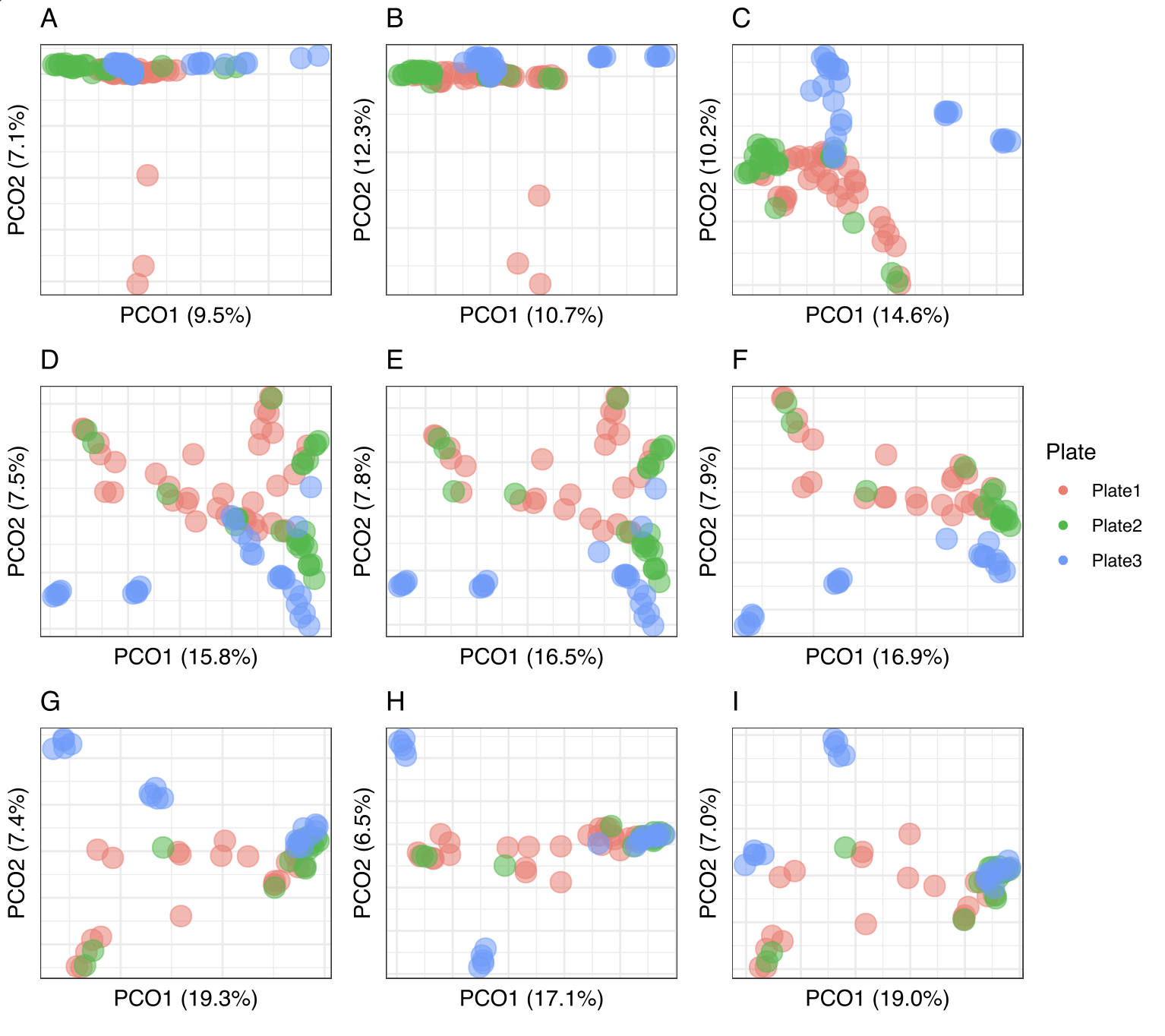


###### **Figure S9.** PCoA of ddRADseq at major filtering steps including A) raw Stacks output, B) minimum quality filtering, C) outlier removal with outliers labeled in A and B, D) SNP missingness <30%, E) sample missingness <50%, F) biased SNPs removed forming the high missingness dataset used in analyses, G) low missingness dataset used in analyses, H) LD-pruned dataset used in analyses, and I) low missingness dataset with LD-pruning. Samples are colored by missingness where darker blues represent lower proportions of uncalled SNPs. Plots F-I suggest that observed population structure is not driven by missing data. Note that technical replicates have not been removed from these visualizations.

**
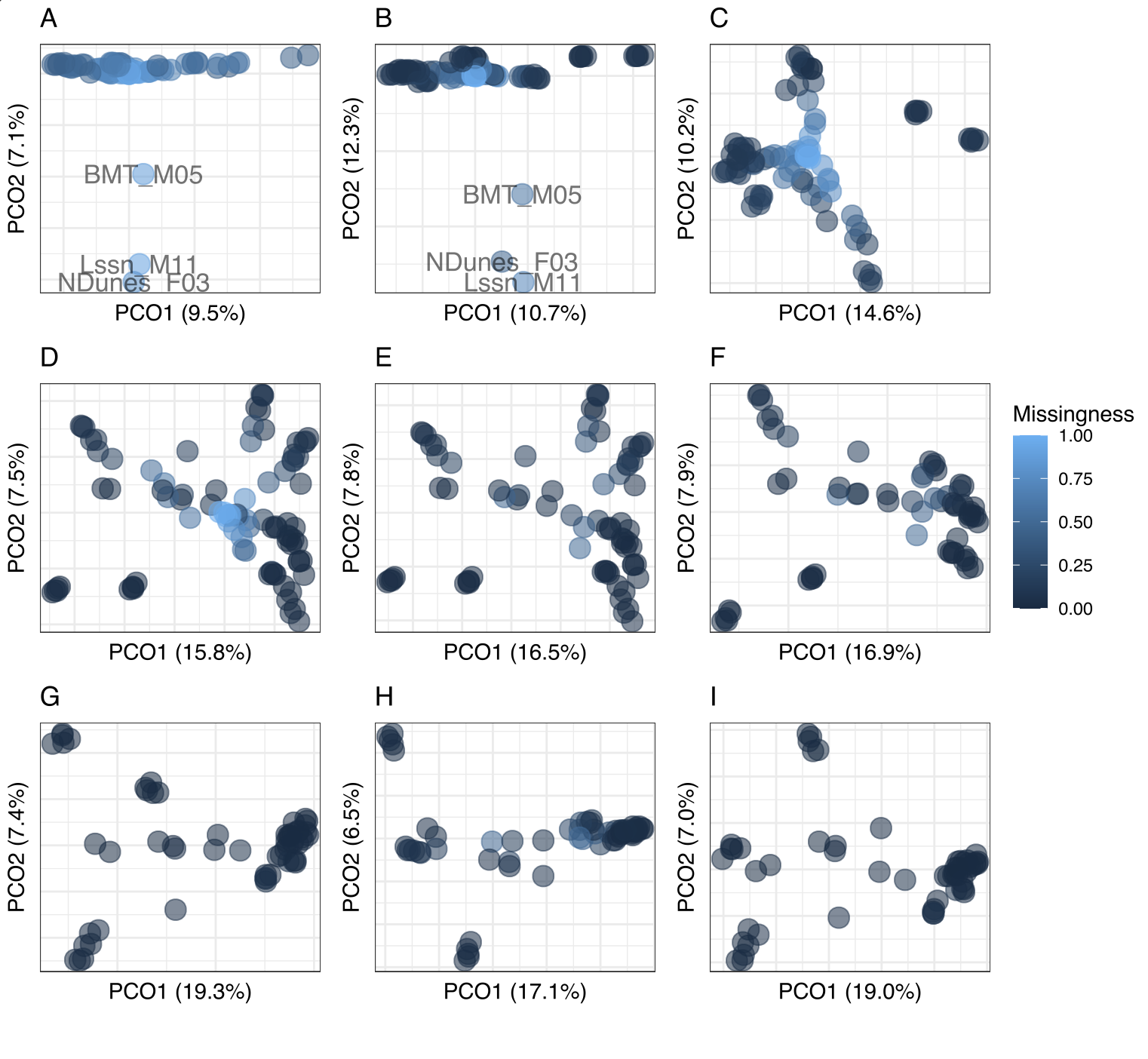
**

###### **Figure S10.** PCoA of ddRADseq at major filtering steps including A) raw Stacks output, B) minimum quality filtering, C) outlier removal, D) SNP missingness <30%, E) sample missingness <50%, F) biased SNPs removed forming the high missingness dataset used in analyses, G) low missingness dataset used in analyses, H) LD-pruned dataset used in analyses, and I) low missingness dataset with LD-pruning. Samples are colored by geographically defined populations. High missingness (F), low missingness (G), and LD-pruned datasets (H) show similar population structure. Note that technical replicates have not been removed from these visualizations.

######
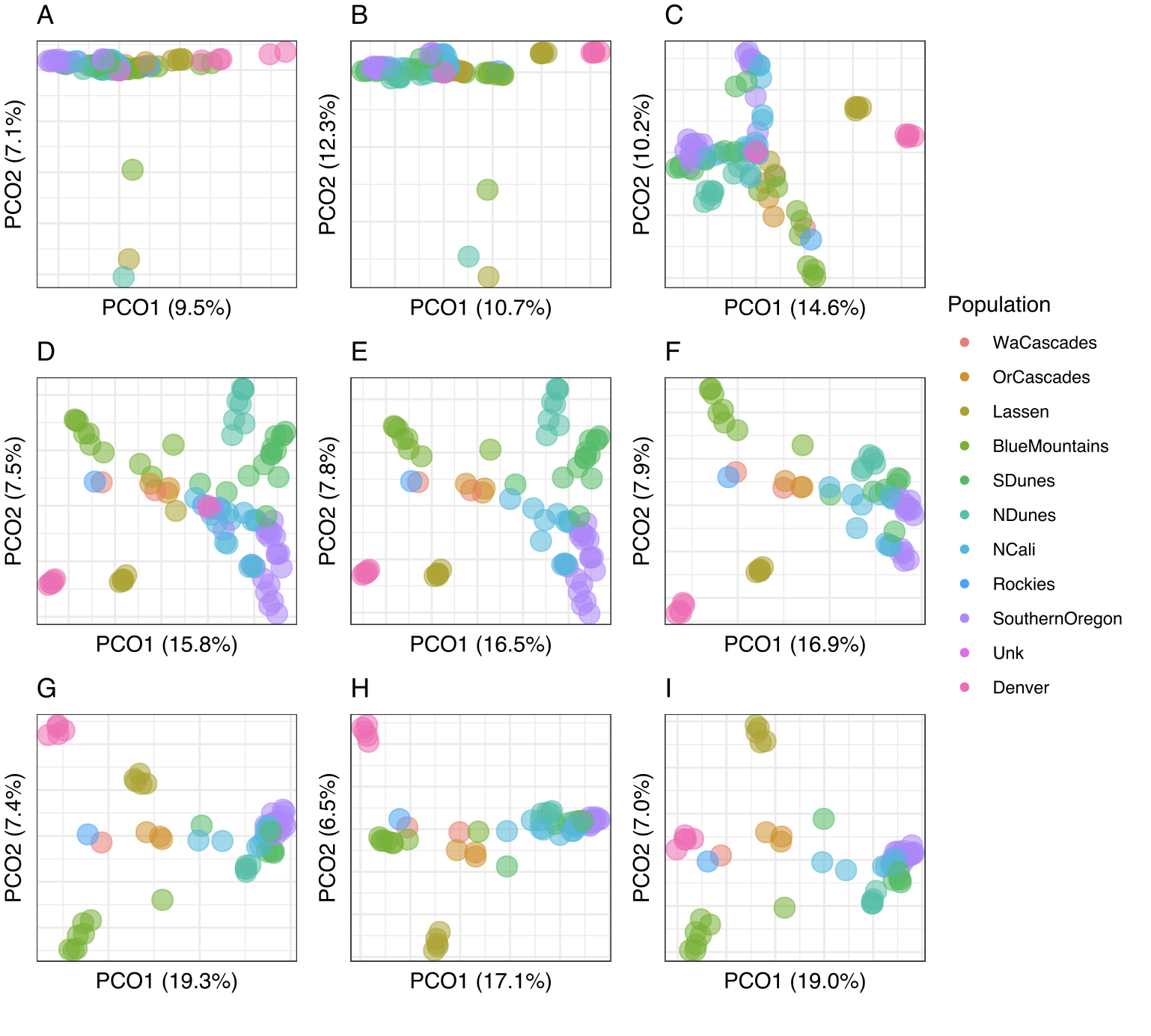


###### **Figure S11.** PCoA results on the high missingness ddRADseq dataset. Samples are colored by proportion of missing SNPs (A) and sequencing depth (B) showing that sample quality is not driving observed genetic structure. C) Plot of additional PCoA axes that explain >5% of variation in the ddRADseq dataset, showing further differentiation between Lassen and Colorado martens is captured by axis 3 and additional variation in Humboldt martens, particularly the North and South Dunes, captured in axis 4.


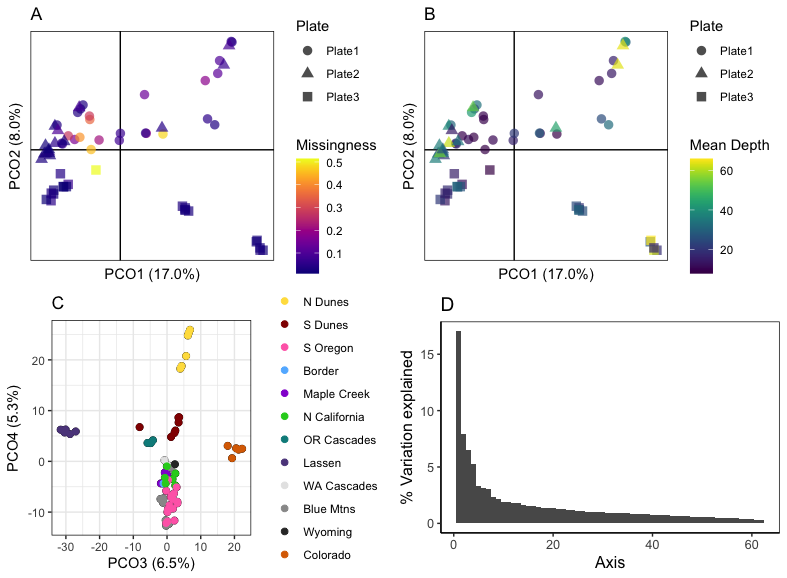


# **Figure S12.** ADMIXTURE ancestry estimates with K=2-9 clusters based on the LD-pruned ddRADseq dataset (12,389 SNPs) for A) all Pacific martens (K=2 used for Fig. 3 in main text) and B) Pacific martens with one in each pair of first-order relatives removed. C) Ancestry estimates for K=2-5 clusters using one sample per area. D) Cross-validation errors and delta log-likelihoods used to evaluate model fit, where lowest CV error and maximum change in log-likelihood reflect best fit. Each bar represents an individual marten, colors represent the fraction of ancestry assigned to each ancestral cluster, white lines separate populations and black lines separate Humboldt and non-Humboldt martens. K=2 clusters, which split Humboldt martens and eastern martens consistently had the best model fit but correlation in intra-population residuals indicated additional unmodeled substructure until K=9 (Figure S13). ‘Trin’ is Maple Creek.


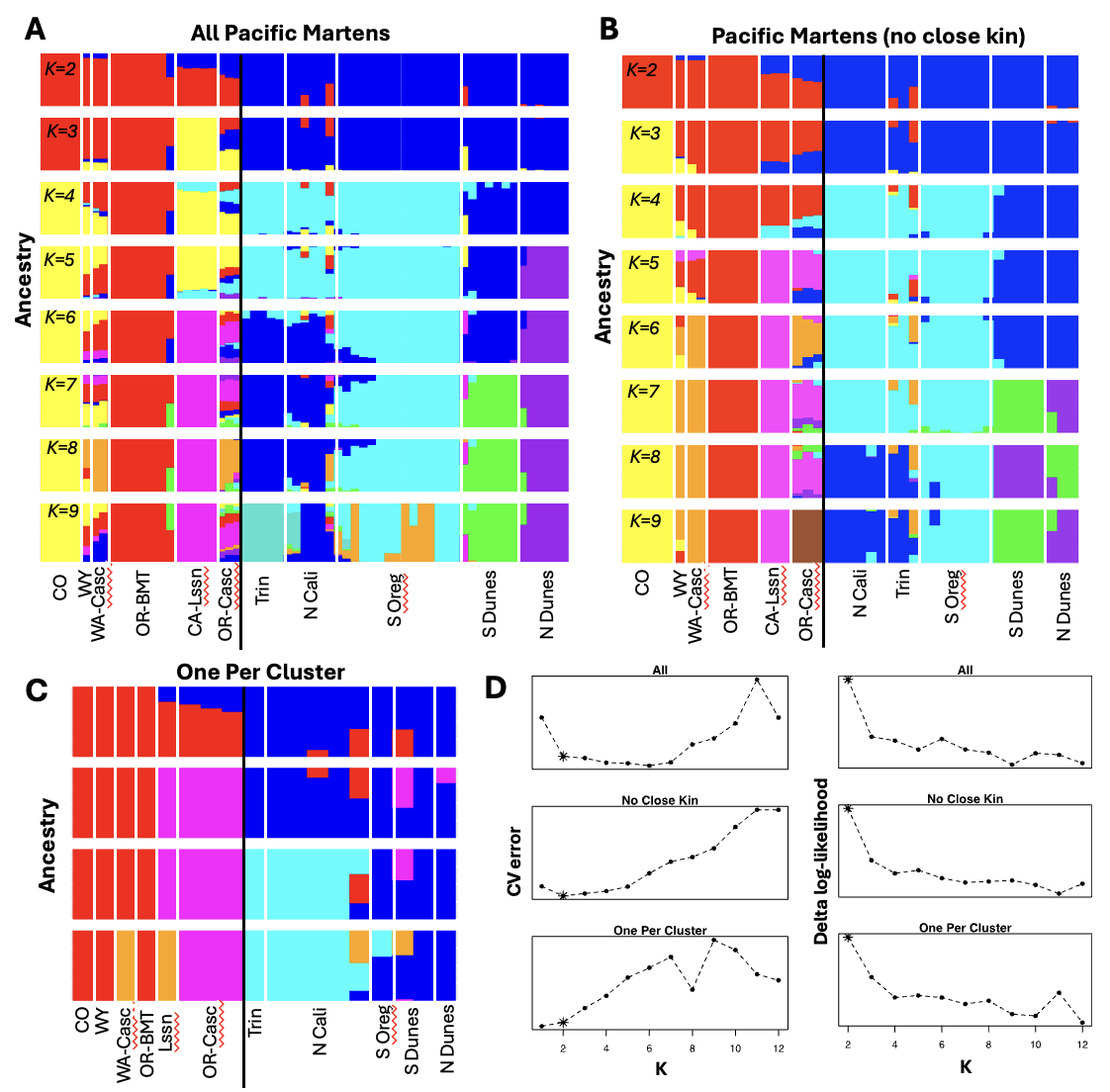


###### **Figure S13.A** evalAdmix results for ADMIXTURE models with K=2-5 clusters including all Pacific martens, all Pacific martens with close kin removed, and one individual per cluster. Pairwise correlations between individuals is shown on the upper triangle while pairwise correlations between populations is shown on the lower triangle, with lines separating populations. Higher positive correlations within a population with higher negative correlations with other populations suggests additional genetic structure that is uncaptured by the model, while high positive correlations within a population with low correlations with other populations suggests high levels of relatedness and/or genetic drift. K=7 best minimized correlations in residuals between populations for the full dataset, however high residual correlations between individuals suggests additional forces driving genetic structure within these clusters.
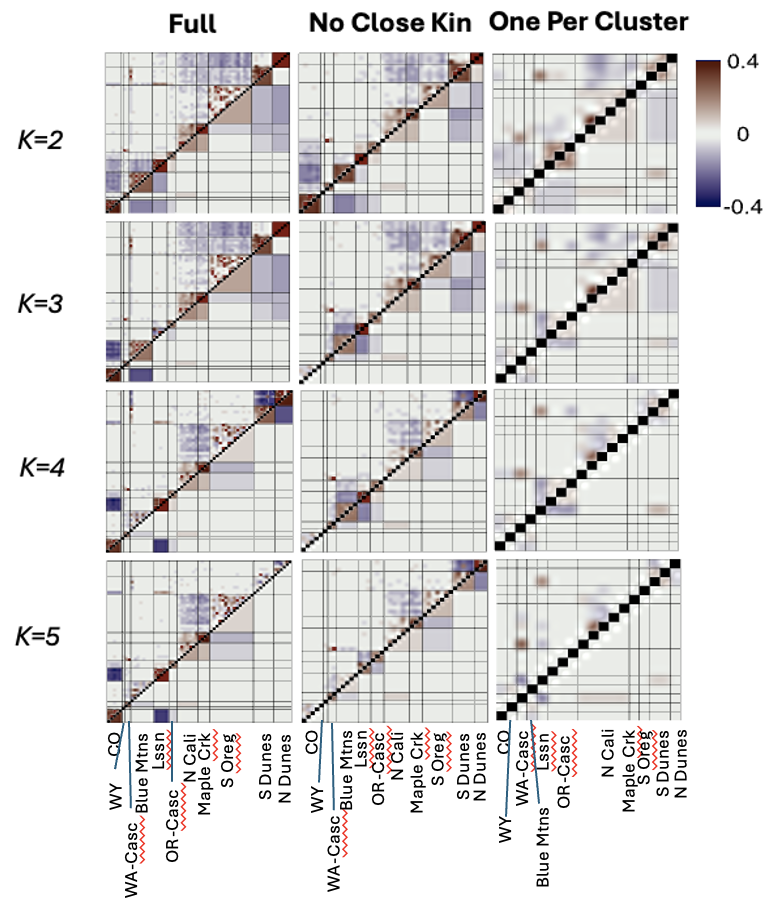


###### **Figure S13.B (continued)** evalAdmix results for ADMIXTURE models with K=6-9 clusters including all Pacific martens, all Pacific martens with close kin removed, and one individual per cluster. Pairwise correlations between individuals is shown on the upper triangle while pairwise correlations between populations is shown on the lower triangle, with lines separating populations. Higher positive correlations within a population with higher negative correlations with other populations suggests additional genetic structure that is uncaptured by the model, while high positive correlations within a population with low correlations with other populations suggests high levels of relatedness and/or genetic drift. K=7 best minimized correlations in residuals between populations for the full dataset, however high residual correlations between individuals suggests additional forces driving genetic structure within these clusters. *
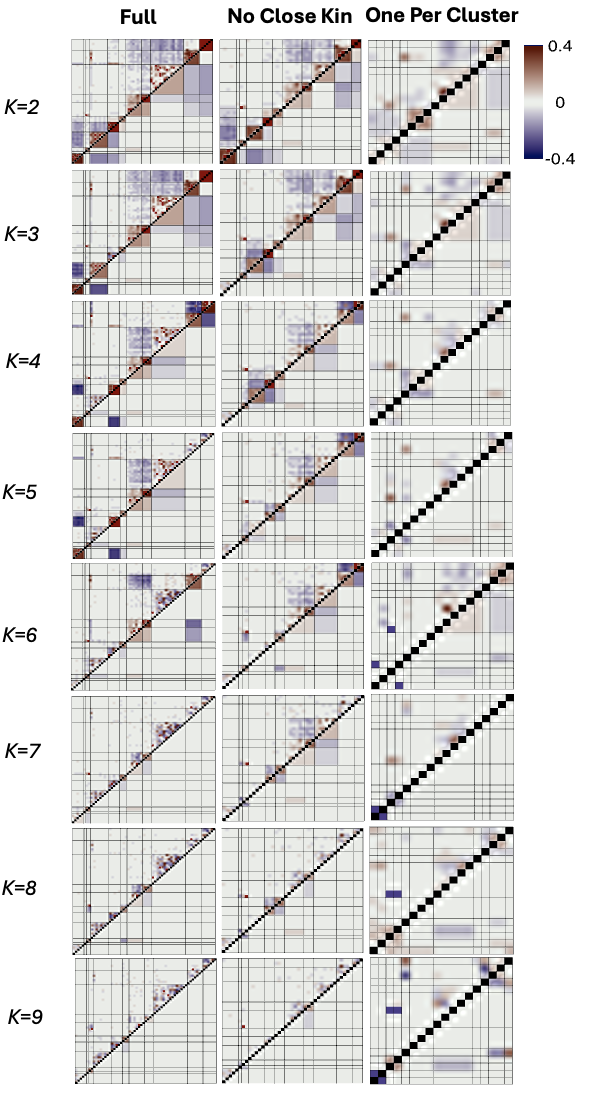
***
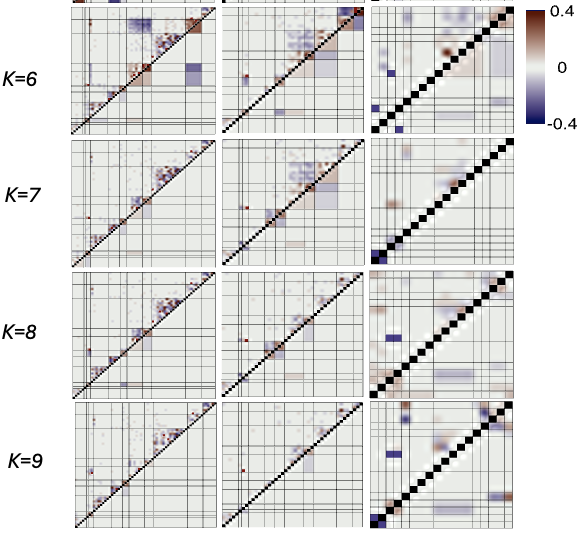
**
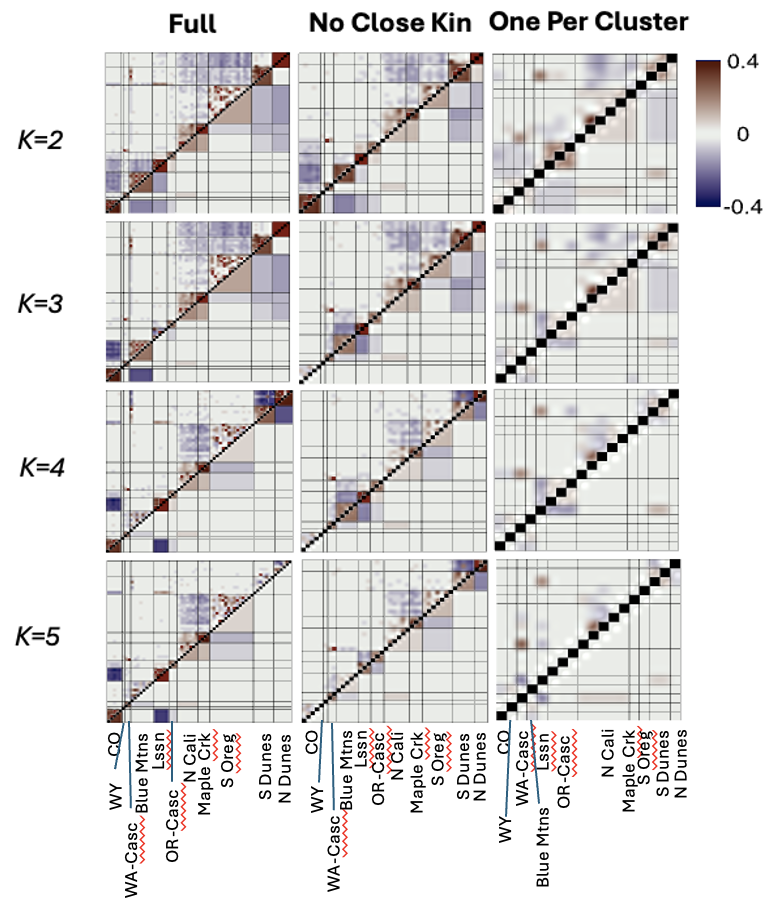


#

###### **Figure S14.** Humboldt marten ADMIXTURE results for K=2-6 ancestral clusters in Humboldt martens based on the full LD-pruned dataset (9,239 SNPs). K=2-5 included in Fig. 4 in main text. ‘Trinidad’ is equivalent to ‘MapleCrk’.**
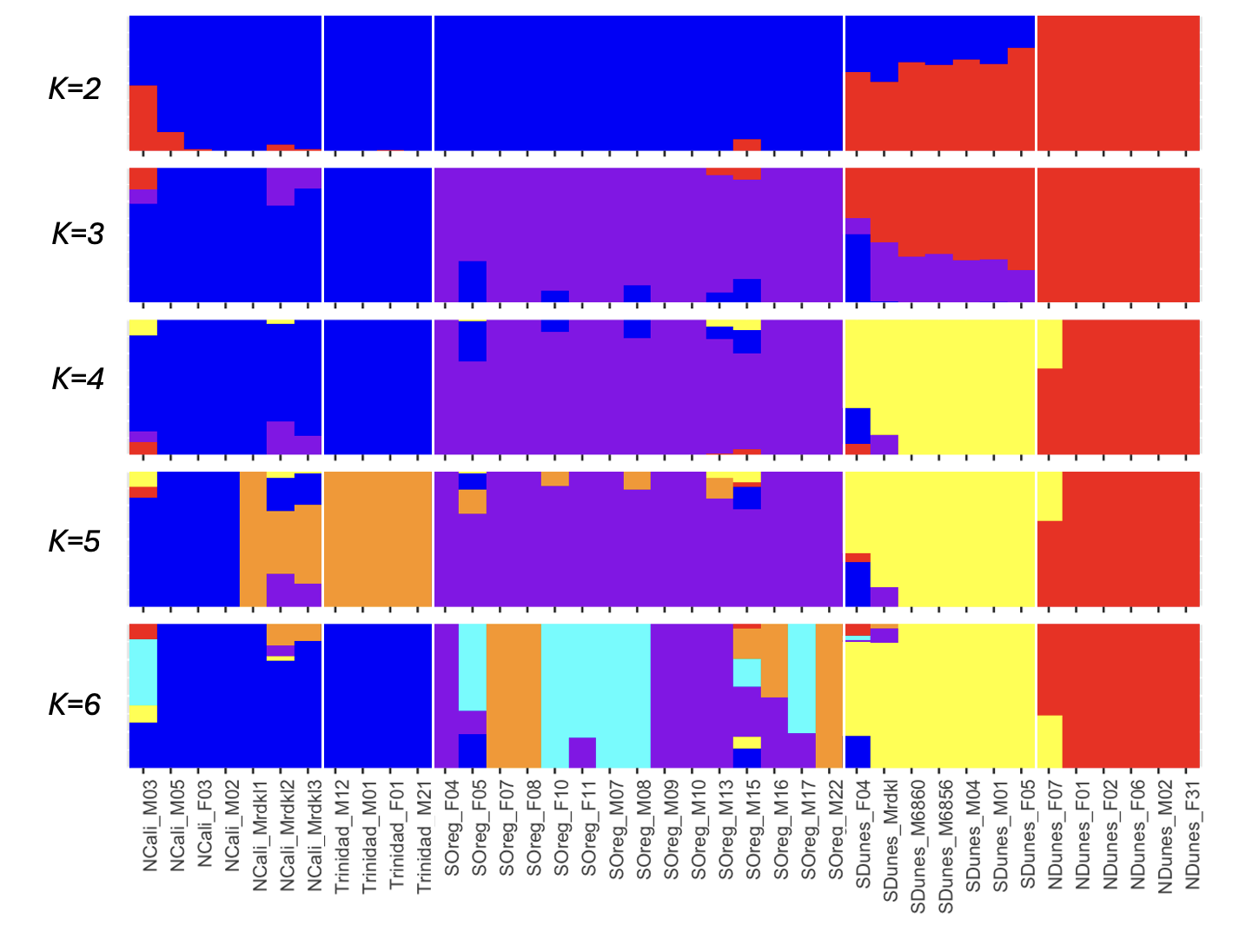
**

**
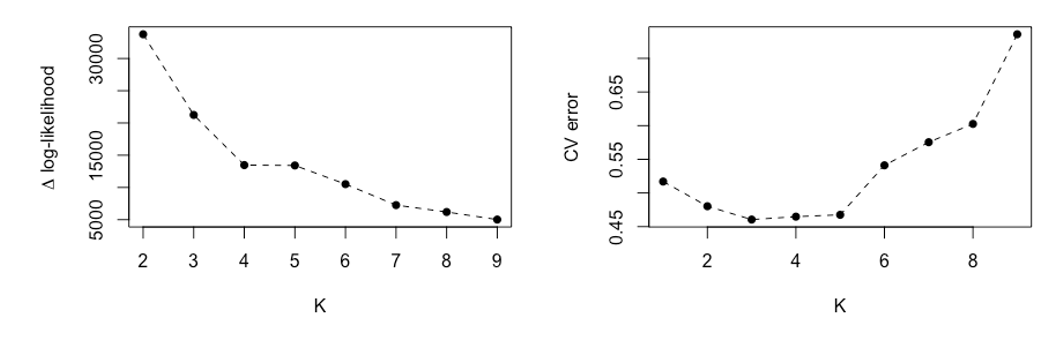
**

###### **Figure S15.** Pairwise correlations between ADMIXTURE model residuals for K=2-6 on the Humboldt marten dataset. Pairwise correlations between individuals is shown on the upper triangle while pairwise correlations between populations is shown on the lower triangle. Higher positive correlations within a population with higher negative correlations with other populations suggest additional substructuring within a cluster, while high positive correlations within a population without negative correlations with other populations suggests high levels of relatedness and/or genetic drift. K=5 best minimized correlations in residuals between populations, however high residual correlations between individuals suggests additional forces driving genetic structure within these clusters. ‘Trinidad’ is equivalent to the ‘Maple Creek’ population in the main text.

**
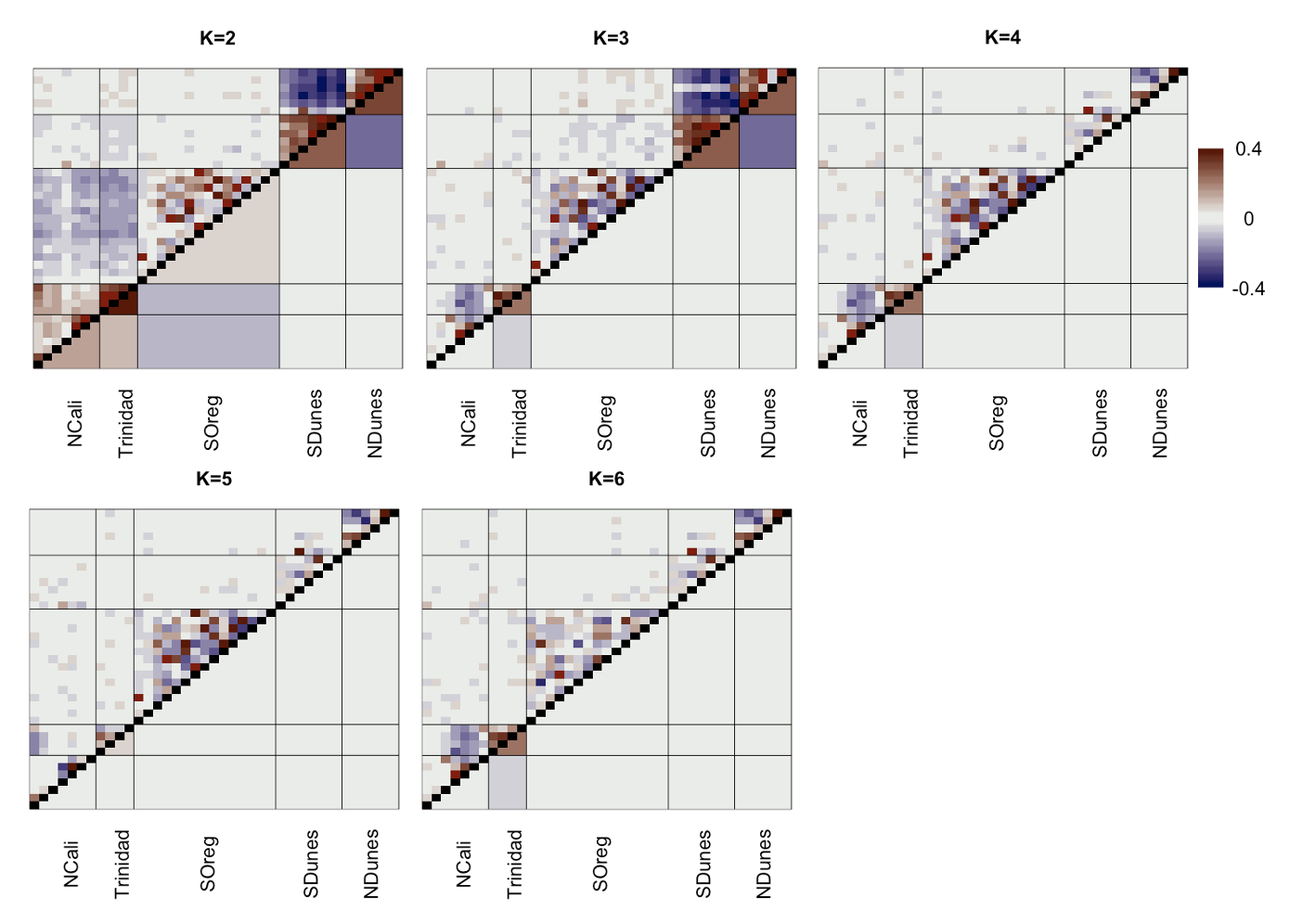
**

###### **Figure S16.** Individual SNP heterozygosity vs. sequencing depth of coverage shows no effect of sample depth on heterozygosity estimates but high heterozygosity in 1 Blue Mountain and 2 Humboldt martens which align with high interclass heterozygosity in triangle plots.

#
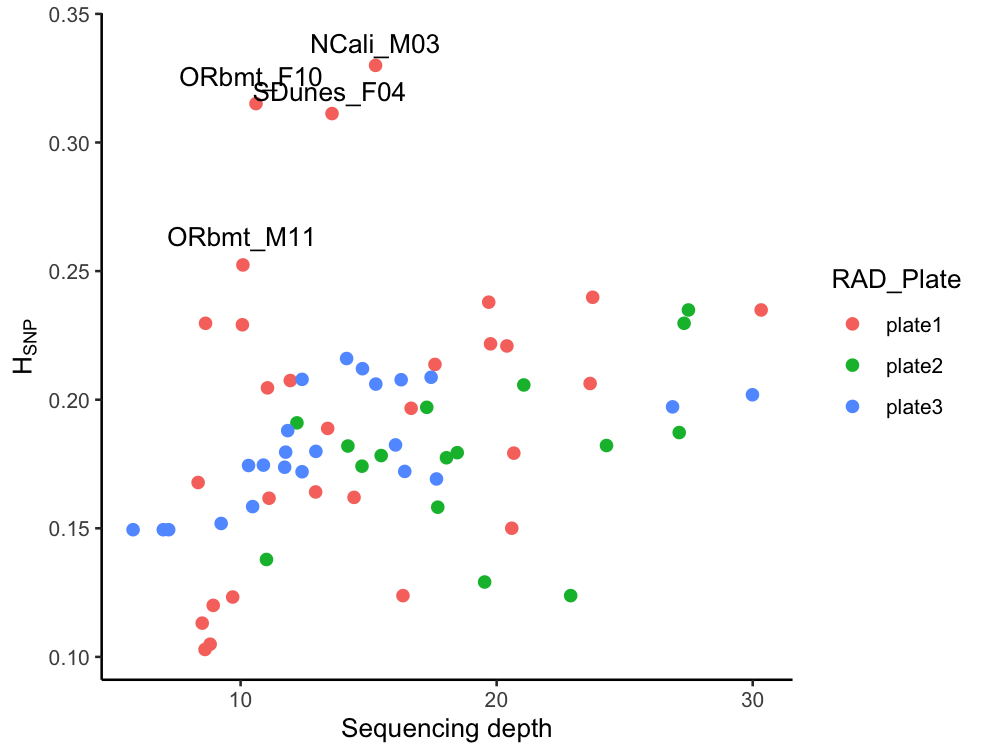


###### **Figure S17.** Comparison of principal coordinates analysis (PCoA) between ddRADseq (12,389 SNPs) and GBAS (98 SNPs) based on 59 individuals genotyped using both methods. A) The first 2 PCoA axes show that both datasets capture the same general patterns with the first axis following a west-east division in genetic variation between Humboldt and eastern montane martens, with western montane martens (Oregon Cascades and Lassen) in between, and the y-axis splitting North Dunes martens from other Humboldt martens. The GBAS panel shows less less clear clustering of populations indicating lower resolution for detecting fine-scale genetic variation. B) mapping of PCO1 showing effective splitting of Humboldt from montane martens in both datasets.

**A**


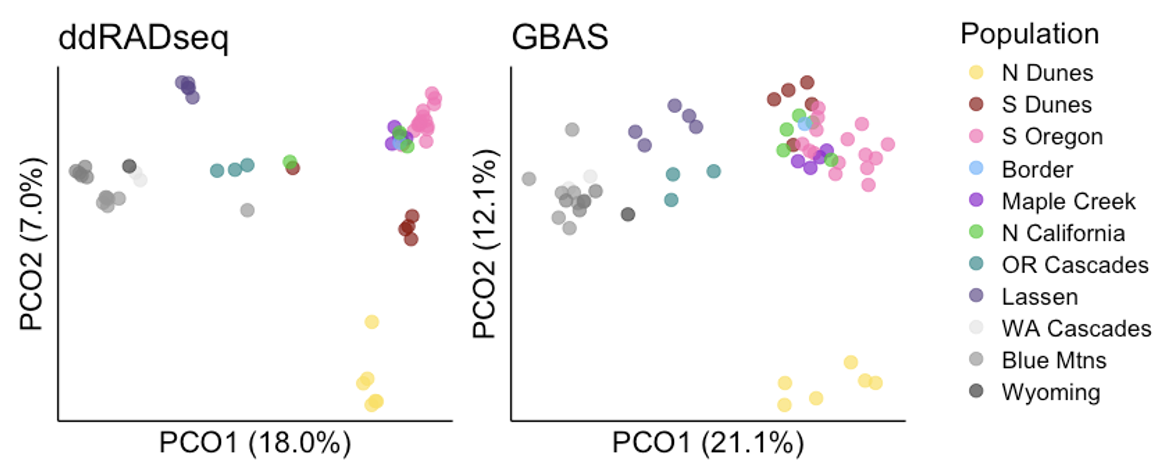


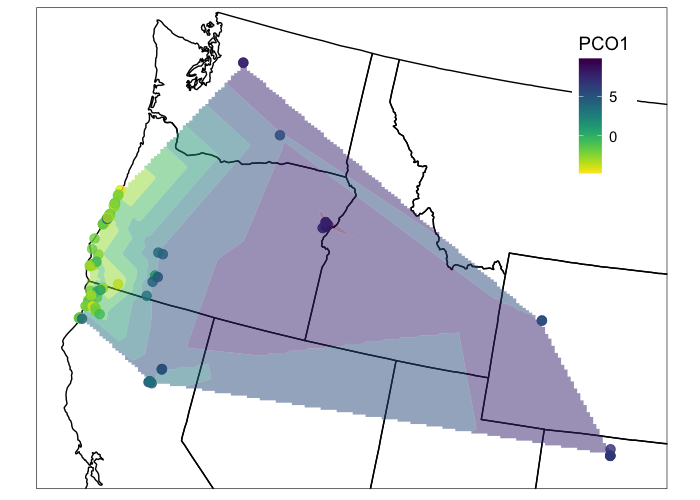

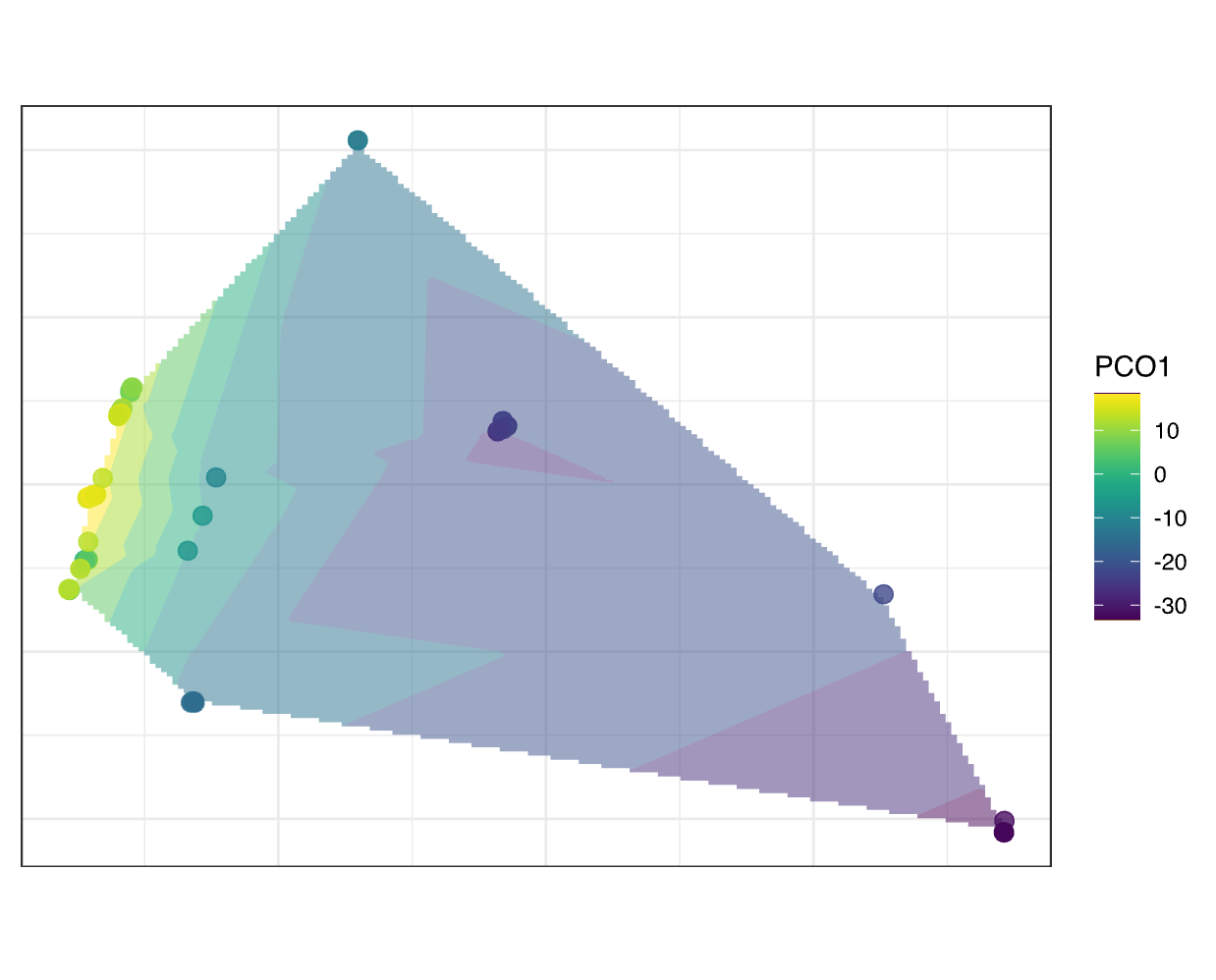

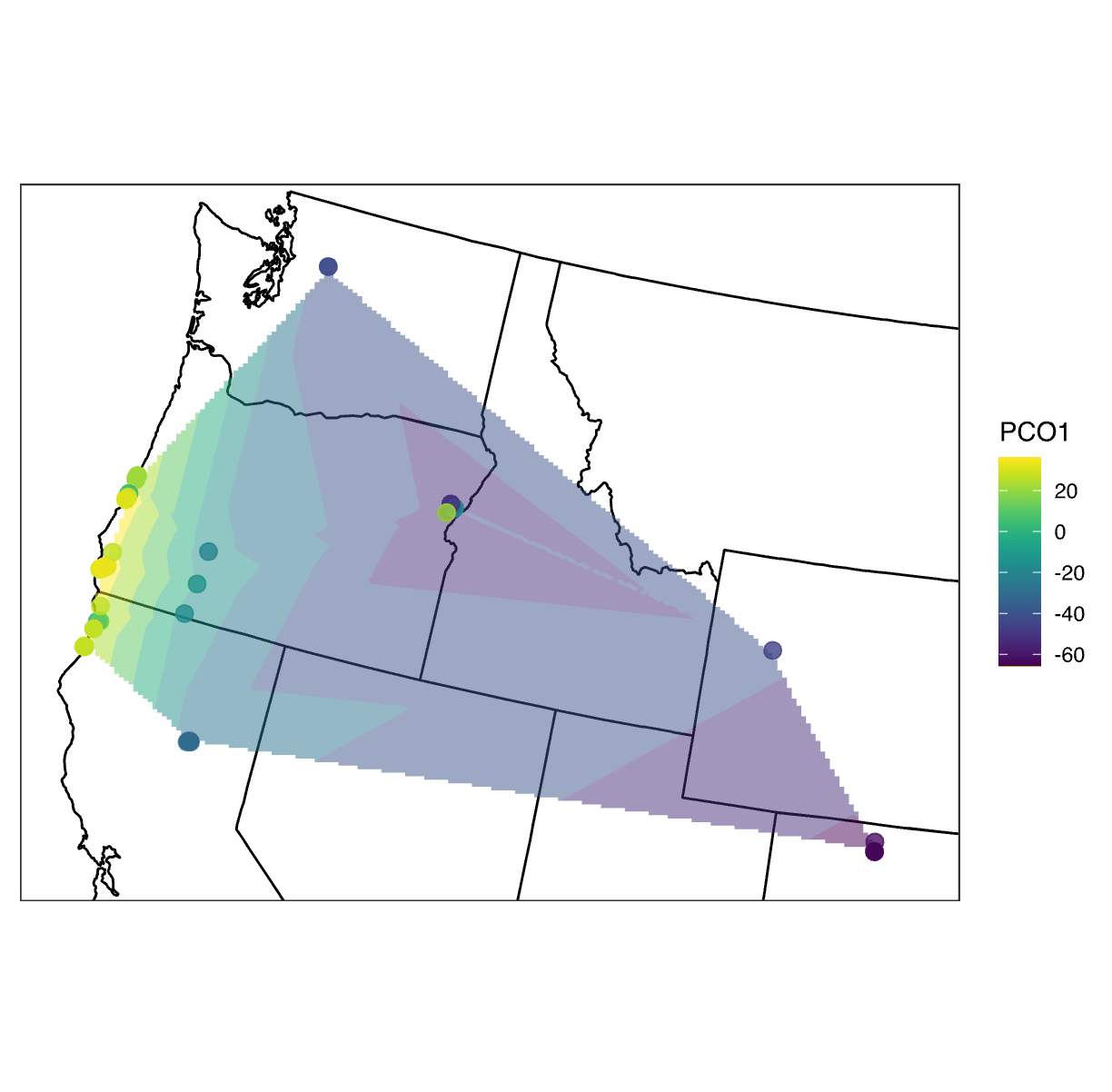
**B**

###### **Figure S18.** Comparison of individual relatedness estimates (β_jj_; Weir and Goudet 2017) between LD-pruned ddRADseq (12,389 SNPs; x-axis) and GBAS (98 SNPs; y-axis) for 59 martens. Each point represents a pairwise comparison between individuals, plots are grouped by population of individual 1, colors represent the population of individual 2, and diagonal line shows a perfect correlation. GBAS relatedness had good concordance with ddRADseq within the N. Dunes, N. California, and Maple Creek Humboldt marten populations but had higher variation within the S. Oregon and S. Dunes populations. GBAS relatedness was overestimated in both western and eastern montane marten populations although was positively correlated with ddRADseq estimates. Inter-population GBAS inter-population estimates were consistently smaller than intra-population estimates except when comparing individuals from closely related populations (e.g., S Oregon vs. N. California vs. Maple Creek). Note that the S. Dunes and N. California populations contained hybrids.

*
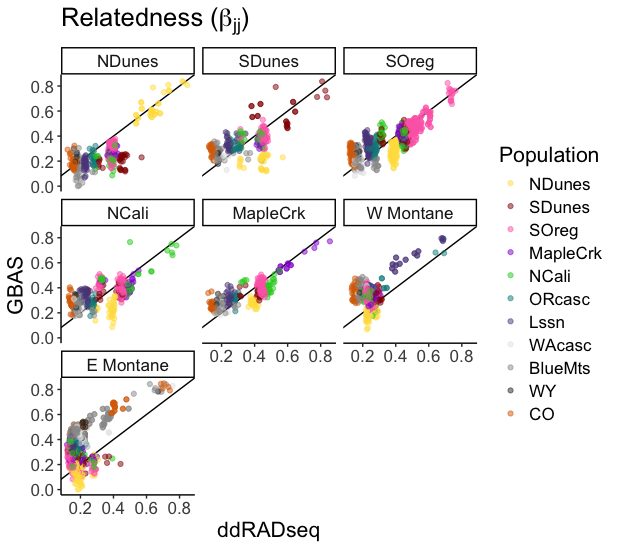
*

###### **Figure S19.** Comparison of genetic diversity (individual heterozygosity H_SNP_) and inbreeding (individual inbreeding coefficient F_β_; Weir and Goudet 2017) estimates for 59 martens genotyped by both ddRADseq (12,389 SNPs) and GBAS (98 SNPs) datasets with lines showing a 1:1 relationship (i.e., perfect concordance). Poor correlation was found between ddRADseq and GBAS estimates, indicating that GBAS is ineffective at estimating genetic diversity and inbreeding.


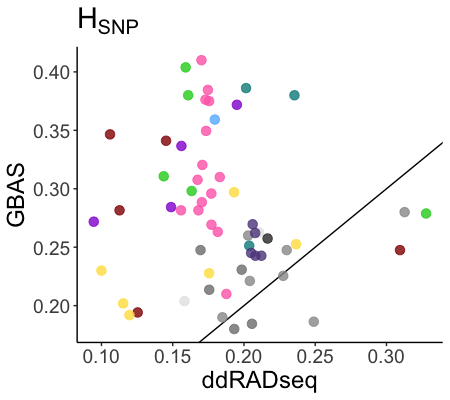

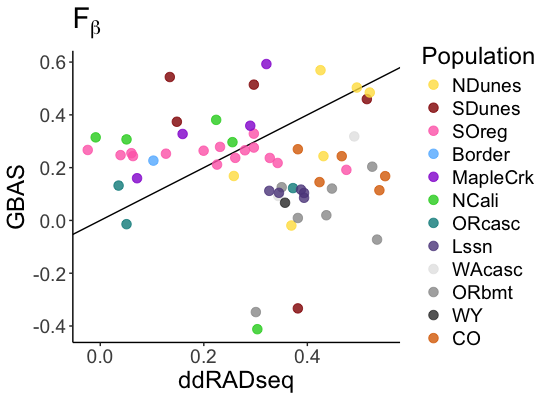


###### **Figure S20.** Principal coordinates analysis (PCoA) for individual Humboldt martens (N=109) based on 98-SNP GBAS panel showing 4 genetic clusters representing the North Dunes OR, North Coast OR, and South Dunes OR, and Southern Oregon through Northern California which shows less resolution than the ddRADseq data (Figure S11). Most variation is captured by splitting the North Dunes from southern populations along axis 1, with North Coast martens intermediate to these groups. Axis 2 captures roughly captures a north-to-south gradient of genetic variation (see Figure 7B in main text).


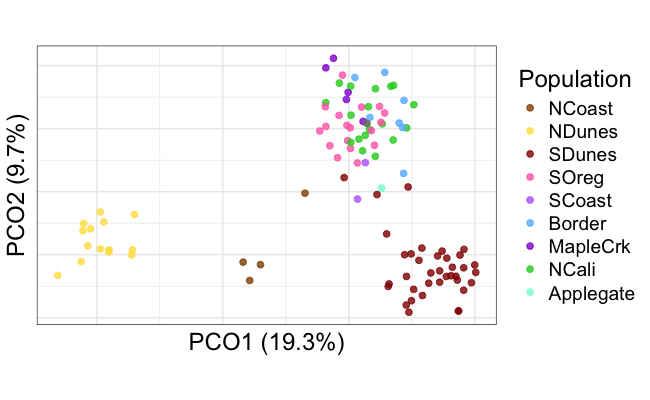


###### **Figure S21.** Maximum-likelihood tree based on GBAS (98 SNPs) showing similar relationships to phylogenomic results (Fig. 2A) but different branching patterns and lower resolution to distinguish between closely related clades (e.g., N. California, S. Oregon, Maple Creek).

###### **Table S1.** Pacific marten sampling with number of individuals (N), geographic regions, year(s) with N in parentheses, sample types with number of samples in parentheses, sources, and datasets included within the paper. Details for samples included in genomic analyses are found in Table S2. Humboldt marten regions in bold.

| **Region** | **N** | **Year** | **Sample Type** | **Source** | **Datasets** |
| --- | --- | --- | --- | --- | --- |
| **North Coast, OR** | 4 | 2017 | Scat (8) | OSU/NCASI | GBAS (4) |
| **North Dunes, OR** | 15 | 2015-2016 (5), 2017 (10) | Live-trapping (5), Roadkill (1), Scat (38) | RMRS, OSU | ddRADseq (6), GBAS (14) |
| **South Dunes, OR** | 39 | 2015-2016 (5), 2017 (1), 2020 (1) | Live-trapping (4), Roadkill (3), Scat (113) | RMRS,  OSU, ODFW | ddRADseq (7), GBAS (37) |
| **South Coast, OR** | 2 | 2017 | Scat (9) | OSU/NCASI | GBAS (2) |
| **Applegate, OR** | 1 | 2019 | Scat (2) | OSU/NCASI | GBAS (1) |
| **Southern Oregon** | 19 | 2017 (3), 2022-2024 (15) | Live-trapping (15), Scat (10) | NCASI, OSU/NCASI | ddRADseq (15), GBAS (19) |
| **OR / CA Border** | 8 | 2017(1), 2020 (6), 2023 (1) | Roadkill (1), Scat (13) | USFWS, /NCASI | ddRADseq (1), GBAS (7) |
| **Northern California** | 19 | 2020 (9), 2022-2024 (9) | Live-trapping (6), Roadkill (2), Hair (2), Scat (24) | NCASI, USFWS, Karuk Tribe, OSU/NCASI | ddRADseq (6), GBAS (19) |
| **Maple Creek, CA** | 5 | Unknown (1), 2022-2024 | Live-trapping (4), Scat (1) | NCASI | ddRADseq (4), GBAS (5) |
| OR Cascades | 9 | Unknown (2), 2023 (1) | Tissue (7), Roadkill (1), Scat (1) | RMRS, ODFW, OSU/NCASI | ddRADseq (3), GBAS (9) |
| Lassen | 11 | 2024 | Live-trapping (9), Scat (3) | RMRS, NCASI | ddRADseq (5), GBAS (11) |
| OR Blue Mountains | 11 | Unknown | Tissue | RMRS | ddRADseq (8), GBAS (10) |
| WA Cascades | 2 | Unknown | Tissue | RMRS | ddRADseq (2), GBAS (2) |
| Colorado | 5 | 2020-2021 | Roadkill | DMNS | ddRADseq (5), GBAS (5) |
| Wyoming | 2 | Unknown | Tissue | RMRS | ddRADseq (1), GBAS (2) |
| *M. americana* (WA) | 1 | Unknown | Tissue | RMRS | GBAS |
| *M. americana* (AK) | 1 | 2024 | Scat | Levi Lab | GBAS |

RMRS = National Wildlife Genomics Institute at the Rocky Mountain Research Station

NCASI = National Council for Air and Stream Improvement, Inc.

ODFW = Oregon Department of Fish and Wildlife

USFWS = U.S. Fish and Wildlife Service

OSU = The Levi Lab at Oregon State University

DMNS = Denver Museum of Nature & Science

###### **Table S2.** Genomic sample data including samples per geographic region, sample sources, year(s) collected, etc.

| **Region** | **N** | **Year(s)** | **Sample Type** | **Source** |
| --- | --- | --- | --- | --- |
| WA Cascades | 2 | Unknown | Tissue | RMRS |
| OR Cascades | 3 | Unknown (2), 2023 (1) | Tissue (2), Roadkill (1) | RMRS (2), ODFW (1) |
| OR Blue Mountains | 8 | Unknown | Tissue | RMRS |
| South OR Dunes | 7 | 2015-2016 (5), 2017 (1), 2020 (1) | Live-trapping (4), Roadkill (3) | RMRS (3),  OSU (1), ODFW (3) |
| Southern Oregon | 15 | 2022-2024 | Live-trapping | NCASI |
| Northern California | 7 | 2022-2024 | Live-trapping (4), roadkill (3) | NCASI (4), USFWS (3) |
| North OR Dunes | 6 | 2015-2016 (5), 2017 (1) | Live-trapping (5), roadkill (1) | RMRS (5), OSU (1) |
| Maple Creek | 4 | 2022-2024 | Live-trapping | NCASI |
| Lassen | 5 | 2024 | Live-trapping | NCASI |
| Colorado | 5 | 2020-2021 | Roadkill | DMNS |
| Wyoming | 1 | Unknown | Tissue | RMRS |

RMRS = National Wildlife Genomics Institute at the Rocky Mountain Research Station

NCASI = National Council for Air and Stream Improvement, Inc.

ODFW = Oregon Department of Fish and Wildlife

USFWS = U.S. Fish and Wildlife Service

OSU = The Levi Lab at Oregon State University

DMNS = Denver Museum of Nature & Science

###### **Table S3.** Details from ddRADseq filtering steps, including commands used and number of SNPs and samples (including technical replicates) remaining after each step. Mean sample depth (DP) and mean sample genotyping rate (% GT) are also presented for each step.

| **Purpose** | **Command** | **# SNPs** | **N** | **DP / % GT** | **Applications** |
| --- | --- | --- | --- | --- | --- |
| Raw Stacks output | Populations | 516,545 | 97 | 14.1 / 29% | - |
| Quality filtering | vcftools --minDP 5 | 516,545 | 97 | 14.1 / 26% | - |
|  | vcftools --mac 2 | 238,002 | 97 | 15.3 / 35% | - |
|  | vcftools --min-meanDP 8 | 75,749 | 97 | 20.1 / 66% | - |
|  | vcftools --minQ 20 | 75,749 | 97 | 20.1 / 66% | - |
| Remove outlier samples | plink --remove <remove.ids> --mac 1 | 46,433 | 94 | 20.5 / 64% | - |
| Remove missingness | plink --geno 0.30 | 15,762 | 94 | 24.3 / 77% | - |
|  | plink --mind 0.50 --mac 1 | 15,299 | 75 | 27.4 / 90% | - |
| Hardy Weinberg equilibrium | plink --hardy  vcftools --exclude <HWD.snps> | 15,252 | 75 | 27.3 / 90% | - |
| Remove paralogs | vcftools --max-meanDP 45 | 14,658 | 75 | 26.0 / 90% | - |
| Remove paralogs | plink --hardy  plink --exclude <Het60.snps> | 14,652 | 75 | 26.0 / 90% | - |
| Remove biased SNPs | bcftools query -f ‘%POS[%AD\t]\n’  vcftools --exclude <AB20AB80.snps> | 14,652 | 75 | 26.0 / 90% | Genotyping error, PVCA |
| Remove technical replicates | plink --keep <keep.ids> | 14,652 | 63 | 26.4 / 92% | PCoA |
| A) Reduce missingness | plink --geno 0.05  plink --mind 0.10 --mac 1 | 3,586 | 58 | 32.6 / 99% | fineRADstructure, IQ-tree |
| B) Remove linked SNPs | plink --indep-pairwise 50 5 0.6 | 12,389 | 63 | 26.2 / 92% | ADMIXTURE, Fst, inbreeding, genetic diversity |
| Low missingness + remove linked SNPs | A+B above | 3,055 | 58 | 30.0 / 99% | Ne estimates |

###### **Table S4.** Bootstrap confidence intervals calculated by taking 999 bootstraps of LD-pruned ddRADseq dataset across SNPs. Humboldt marten populations in bold.

| **ddRADseq (12,389 SNPs)** | | |  |  |  |  |  |  |  |  |  |  |
| --- | --- | --- | --- | --- | --- | --- | --- | --- | --- | --- | --- | --- |
|  | N | **N. Dunes** | **S. Dunes** | **S. Oregon** | **N. California** | **Maple Crk** | OR Cascades | Lassen CA | WA Cascade | Blue Mtns | Wyoming | Colorado |
| **N. Dunes** | 6 | - | 0.242−0.258 | 0.248−0.263 | 0.231−0.247 | 0.320−0.340 | 0.300−0.319 | 0.380−0.397 | 0.452−0.475 | 0.330−0.347 | 0.471−0.494 | 0.414−0.431 |
| **S. Dunes** | 7 | 0.242−0.258 | - | 0.150−0.163 | 0.141−0.154 | 0.219−0.236 | 0.236−0.254 | 0.317−0.334 | 0.372−0.395 | 0.297−0.311 | 0.371−0.394 | 0.379−0.398 |
| **S. Oregon** | 15 | 0.248−0.263 | 0.150−0.163 | - | 0.090-0.100 | 0.151−0.165 | 0.233−0.250 | 0.313−0.329 | 0.365−0.387 | 0.311−0.326 | 0.361−0.384 | 0.378−0.395 |
| **N. California** | 7 | 0.231−0.247 | 0.141−0.154 | 0.090-0.100 | - | 0.068−0.080 | 0.179−0.195 | 0.266−0.282 | 0.282−0.303 | 0.250−0.264 | 0.288−0.311 | 0.321−0.336 |
| **Maple Creek** | 4 | 0.320−0.340 | 0.219−0.236 | 0.151−0.165 | 0.068−0.080 | - | 0.235−0.255 | 0.324−0.343 | 0.392−0.417 | 0.295−0.311 | 0.405−0.430 | 0.382−0.401 |
| OR Cascades | 3 | 0.300−0.319 | 0.236−0.254 | 0.233−0.250 | 0.179−0.195 | 0.235−0.255 | - | 0.194−0.211 | 0.169−0.195 | 0.181−0.197 | 0.142−0.170 | 0.249−0.267 |
| Lassen CA | 5 | 0.380−0.397 | 0.317−0.334 | 0.313−0.329 | 0.266−0.282 | 0.324−0.343 | 0.194−0.211 | - | 0.300−0.317 | 0.254−0.268 | 0.289−0.312 | 0.317−0.333 |
| WA Cascades | 2 | 0.452−0.475 | 0.372−0.395 | 0.365−0.387 | 0.282−0.303 | 0.392−0.417 | 0.169−0.195 | 0.300−0.317 | - | 0.194−0.215 | 0.056−0.084 | 0.249−0.271 |
| Blue Mtns OR | 8 | 0.330−0.347 | 0.297−0.311 | 0.311−0.326 | 0.250−0.264 | 0.295−0.311 | 0.181−0.197 | 0.254−0.268 | 0.194−0.215 | - | 0.169−0.191 | 0.248−0.262 |
| Wyoming | 1 | 0.471−0.494 | 0.371−0.394 | 0.361−0.384 | 0.288−0.311 | 0.405−0.430 | 0.142−0.170 | 0.289−0.312 | 0.056−0.084 | 0.169−0.191 | - | 0.203−0.228 |
| Colorado | 5 | 0.414−0.431 | 0.379−0.398 | 0.378−0.395 | 0.321−0.336 | 0.382−0.401 | 0.249−0.267 | 0.317−0.333 | 0.249−0.271 | 0.248−0.262 | 0.203−0.228 | - |

###### **Table S5.** Sex primer sequences, region targeted, species targeted/developed for, and sources.

| **ID** | **FWD (5’-3’)** | **REV (5’-3’)** | **Region** | **Target** | **Source** |
| --- | --- | --- | --- | --- | --- |
| MMX | GGCAGAGCAACCCTGTCATAA | GGGCCTGAGGTTGGTACCACCA | ZFX | *Martes martes* | Mullins et al. 2009 |
| MMY | GCATTGGGCTCCCTGCT | AGATATCCAAATACATGTGGCTTTAAATG | ZFY | *Martes martes* | Mullins et al. 2009 |
| ZFXY w542/3 | TTCCAGGCAGTACCAAACAG | AGGAAATCATTCATGAATATCACT | ZFX/Y | *Martes americana* | Lucid et al. 2020 |
| *SRY MAAM-1 | GTGATCAAAGGCGCAAGGTG | CCTGTATTCTCTGCGCCTCC | SRY | *Martes americana* | this paper |
| *SRY MAAM-4 | TCAGAGATCAGCAAGCAGCT | TGCGCCTCCTCGAAGAATG | SRY | *Martes americana* | this paper |
| ZFY MAMA-1 | CCATCATGATCCCAGGGTCC | AATCACATGCAGGGGGAGTG | ZFXY | *Martes martes* | this paper |

* Primer pairs included for sex identification in our final Pacific marten panel.

**Table S6.** GBAS primer sequences (without adapters), concentration of primer stocks (Stock), volumes of each primer added to make 5X FWD and REV primer mixes (Vol), concentrations of primers in 5X mix (5X conc.) and concentration in multiplex PCR (1X conc.), and target sequence in Pacific martens with FWD primers in blue, REV primers in orange, and SNP locations in red with possible alleles in brackets.

| **PrimerID** | **FWD (5' --> 3')** | **REV (5'-->3')** | | **Stock (µM)** | | **Vol (µl)** | **5X conc.** | | **1X conc.** | | **Sequence** | | **Notes** |  |  |  |
| --- | --- | --- | --- | --- | --- | --- | --- | --- | --- | --- | --- | --- | --- | --- | --- | --- |
| **MC_2** | CAGGAGCAGACGGTTCAG | AGTTGGAACTTGGAGTGTGG | | 100 | | 6.0 | 0.75 | | 0.15 | | CAGGAGCAGACGGTTCAGGGGCGGTGTCCAGAGTGACACCCGC[AG]GACGCTGCCACACTCCAAGTTCCAACT | |  |  |  |  |
| **MC_3** | TCACCACACAAGTGCCTTC | AGGTTATCTCCTGATTGGCAG | | 100 | | 12.0 | 1.5 | | 0.3 | | TCACCACACAAGTGCCTTCCTGATGTACACGGCATGTGTCTGTCC[AC]CAGTGTGCGCCTAGCAGGAGAATCATGGGGTTGTAGGGGGTGCAGGCTTGTAACTGCCAATCAGGAGATAACCT | |  |  |  |  |
| **MC_4** | GCAGGCCCCATTATGCAG | ACCTTTGATGGGCAGAATTTC | | 100 | | 14.4 | 1.8 | | 0.36 | | GCAGGCCCCATTATGCAGAATCACCTCCAGGTCCTGCTCCTTGTTTTGAAACAAGA[CT]GTACGAACAGCTGCCAGTCAGCTTGAAATTCTGCCCATCAAAGGT | |  | |  |  |
| **MC_5** | TGCAGAGACGCCACAGAG | AAGATAGAGGTGAGGTCCCC | | 100 | | 5.4 | 0.675 | | 0.135 | | TGCAGAGACGCCACAGAGGAACGGCTGTCCCCTGGCCACAAGAGGGCAGAGC[GC]GACGGGGACCTCACCTCTATCTT | |  | |  |  |
| **MC_6** | TCTCCTAAGCCAGAACTCCC | CGGTTCTAGACGTTCAGTCC | | 100 | | 16.0 | 2 | | 0.4 | | TCTCCTAAGCCAGAACTCCCAAAGTTTACTTCTCCACTCC[TC]AAAATGCAAGTCATCCCCACGGGGACTAGCCTTCACAGTGGGCAGGGAGGACTGAACGTCTAGAACCG | |  | |  |  |
| **MC_7** | TGCAGGGACAGAGCCTTC | GATCTCGGGCAGCATCCC | | 100 | | 8.0 | 1 | | 0.2 | | TGCAGGGACAGAGCCTTCTGGGTCTGGGGTCCCTGTGGACATCCCCAGACTCCCAGCCGAGACCAGCTGTAGCTCACCA[AG]CTGTTTGGGGGATGCTGCCCGAGATC | |  | |  |  |
| **MC_8** | GGATGGGCTTGTATTATTGAGC | CGGGCAGTCTGACTCCAG | | 100 | | 10.8 | 1.35 | | 0.27 | | GGATGGGCTTGTATTATTGAGCAGAACTTCAGAGTTTCAGGACCAAGAA[GA]GCAGGGAGTAAGGGGCGGTGCCCAGAGATTGAACAGGTAGGCTCTGGAGTCAGACTGCCCG | |  | |  |  |
| **MC_9** | CCCAGTTTCTCCCGCATG | AGATTCTAAACCAATCACGGTTC | | 100 | | 8.0 | 1 | | 0.2 | | CCCAGTTTCTCCCGCATGTCAGAAGGGCCCTCCTCCCGACCCCA[AT]GGACGAACCGTGATTGGTTTAGAATCT | |  | |  |  |
| **MC_10** | ACTCACAGATGAGCGTGATG | GGAACAGACGAGACTCTCAG | | 100 | | 8.0 | 1 | | 0.2 | | ACTCACAGATGAGCGTGATGGGGAGGAAGGTGTCGTCGTGACGCGGT[GA]CAGGGGGAGCGGCAGGTGTGAGAAACTGAGAGTCTCGTCTGTTCC | |  | |  |  |
| **MC_11** | GCAGTTGAAGATGAATCCGC | CGGGTAATTGCCTCACAGAC | | 100 | | 8.0 | 1 | | 0.2 | | GCAGTTGAAGATGAATCCGCACGTTAAGCCAAAACATCACATACAGTAATGATAAAGCCTCCAGACAGAGCACAGAAAGGTCCATAAGGGTCTG[CT]TGAGTCTGTGAGGCAATTACCCG | |  | |  |  |
| **MC_12** | CAATTTTGAGGCCAGTGTATG | CTGTGGCAGTCCTTCCATG | | 100 | | 6.4 | 0.8 | | 0.16 | | CAATTTTGAGGCCAGTGTATGATCCACTGTAACATCTGA[CT]CACAGAGGGGAGACCATGGAAGGACTGCCACAG | | amplifies SNP in *americana* | |  |  |
| **MC_13** | CAGGAAGCAGATCCATTTTAGC | CGGATGTCAGATGAGGATCG | | 100 | | 8.0 | 1 | | 0.2 | | CAGGAAGCAGATCCATTTTAGCAACGCTCCCGCCTCA[TC]TCCCGAATTCTCCTGGCGATCCTCATCTGACATCCG | |  | |  |  |
| **MC_14** | GGGCACACAATGAAAAGACC | ATGCCAAACAAAGGGAGTTG | | 100 | | 12.0 | 1.5 | | 0.3 | | GGGCACACAATGAAAAGACCAAGTAGTGAGCACGGGGA[AG]GGGGTGTCAACCAACTCCCTTTGTTTGGCAT | |  | |  |  |
| **MC_16** | CTCCACTTGACCTTTGGGG | CGGAAACAGTGCATCCCC | | 100 | | 6.5 | 0.812 | | 0.162 | | CCACTTGACCTTTGGGGCAGTCACCTTGTGCAGTGAACAAACTGCATGTT[TC]ACATTCTTTGCCCACATTGGGGATGCACTGTTTCCG | |  | |  |  |
| **MC_18** | GAGAGCAAGCGCAACCAG | ATTCTGCCTCCTTGCTTCAC | | 100 | | 5.4 | 0.675 | | 0.135 | | GAGAGCAAGCGCAACCAGCCA[CT]ATCCCTGCACCTGCTTGTAGGTGTGGACTGAGTGAAGCAAGGAGGCAGAAT | | amplifies SNP in *americana* | |  |  |
| **MC_19** | CACAGTTGACCACCCCAG | CAGCCAAAATCTGTCCAGTG | | 100 | | 8.0 | 1 | | 0.2 | | CACAGTTGACCACCCCAGGGTGGCTGTCCGCAGGGCCCACCCCACACCCTGACCACCCTCAGGTCACT[TC]TCAGGGTGACGTCACTGGCTTCACTGGACAGATTTTGGCTG | |  | |  |  |
| **MC_21** | TGCAGCCAAGAAGATTCCAG | TTGGGAGAGCTTCATTTCGG | | 100 | | 19.2 | 2.4 | | 0.48 | | TGCAGCCAAGAAGATTCCAGACAGCCCAGAGGCCAAGGCGGAAGGCTGACCAGTACTGGCTC[CT]CCAAGCCTGGGGGAGGACACAGCAGGGCCACTCCGAAATGAAGCTCTCCCAA | | amplifies SNP in *americana* | |  |  |
| **MC_22** | AGAGCTTGATCTTGTTTACTCC | GTCTCGCATGTGTCCCTTG | | 100 | | 14.4 | 1.8 | | 0.36 | | AGAGCTTGATCTTGTTTACTCCTGCATGTTTTTCAGAGAGTAGTTACCATAGCAACTCCTTT[CG]TCAAGGGACACATGCGAGAC | |  | |  |  |
| **MC_24B** | TGGCGAAGGGTGAGGTAC | TTGTAGCTGATGCTCCACAC | | 100 | | 8.0 | 1 | | 0.2 | | TGGCGAAGGGTGAGGTACGCCCAGGTGCCAAAGATACTTGGAAAGGCCCAGC[AG]CCCTGTCAAGCTGTGTGGAGCATCAGCTACAA | |  | |  |  |
| **MC_25** | TCAGGCCTAGCAGCAGAG | CTTCACGTCCAGTGGGAAC | | 100 | | 7.2 | 0.9 | | 0.18 | | TCAGGCCTAGCAGCAGAGGCGCGACCTGGATTACCCCACGGCCAC[GA]CTGGGCGGGGCCTGTCACCGTTCCCACTGGACGTGAAG | |  | |  |  |
| **MC_26** | TGATTCCTTGTGCCCCATC | AGTCTATGGTACATGCAGGG | | 100 | | 7.2 | 0.9 | | 0.18 | | TGATTCCTTGTGCCCCATCCTACATG[CT]GTCACCTGCCCACGCTTACAGGCCTCAGAATCGTGGCCACGGAAGCCCGAGCTCTGGCATGGATCCCTGCATGTACCATAGACT | | amplifies SNP in *americana* | |  |  |
| **MC_29** | TGCAGCCTGAGTTTCTTGG | AGAGCACTTACGTCCTGTTC | | 100 | | 17.3 | 2.16 | | 0.432 | | TGCAGCCTGAGTTTCTTGGGGGAAGTGAGGTGGGGAGG[CA]AGATTCATACT[AG]ATTGTGGAGCAGTTTCCTCTCCCAGAGGCCCAGGGAACAGGACGTAAGTGCTCT | | 4 haplotypes | |  |  |
| **MC_30** | CTCTTCGAAGGAGCCAAGC | TCAGCAGTCCCTTACTCCTG | | 100 | | 16.0 | 2 | | 0.4 | | CTCTTCGAAGGAGCCAAGCGTGGCTCCTCC[AC]TGATTGTGGTGA[TC]CAGGCCTCCGCCAGGGAGGCAGGAGTAAGGGACTGCTGA | | SNPs fully linked: AT, CC | |  |  |
| **MC_31** | CAGGTCGATTGTCGCCAG | TGGGCCAGTATTCCTTTAGC | | 100 | | 7.2 | 0.9 | | 0.18 | | CAGGTCGATTGTCGCCAGAGGAACCTGCCTCATCAGGTAGGAATGGC[AG]TGGCTAAAGGAATACTGGCCCA | |  | |  |  |
| **MC_32** | ACCAGAGTTGAAGTCAAATGC | GAGCAGAGCAGACATTTTAGG | | 100 | | 12.0 | 1.5 | | 0.3 | | ACCAGAGTTGAAGTCAAATGCAGAAGACA[GA]TGTGGAAGTGAAACTCGGTCAGTAACATCCTAAAATGTCTGCTCTGCTC | |  | |  |  |
| **MC_33** | GCAGCTAATCTGGGTGATAAG | AGATGATGGGACTTGGATCAG | | 100 | | 16.0 | 2 | | 0.4 | | GCAGCTAATCTGGGTGATAAGTATGTCTGTGTGTACATCTGTAAGCTCTTCGAGGACAGGGAACGTG[CT]CTGATCCAAGTCCCATCATCT | |  | |  |  |
| **MC_34** | GAAGACGTCGGGTGGTGG | ATCCTCCCCTTTCCCAGAG | | 100 | | 6.0 | 0.75 | | 0.15 | | GAAGACGTCGGGTGGTGGGATCAGTCCAGGGC[TC]AGAGTGTTCGGAGAGGGTATGTCCTCGTGGCAGTGACTTTCTCCCTGGCCTCTGGGAAAGGGGAGGAT | | amplifies SNP in *americana* | |  |  |
| **MC_35** | TGTGGTACAACGGTGTGAG | GTGCTCTTGTTTTCCAGACC | | 100 | | 6.6 | 0.825 | | 0.165 | | TGTGGTACAACGGTGTGAGGCCGTAGCTGTCTTTGTAATC[GA]GGGGATGCTCCCAGCTCCAAAAGGGTCTGGAAAACAAGAGCAC | |  | |  |  |
| **MC_37** | ACGTACAGCAGGGCATAAAC | GCAGGCAAGTTTAACCGAG | | 100 | | 5.9 | 0.742 | | 0.148 | | ACGTACAGCAGGGCATAAACAGGGGACA[CT]GTACCCTCTCACAACACCTCCACCTCGGTTAAACTTGCCTGC | |  | |  |  |
| **MC_38** | AGAAAGAGCGAGTCTGTTGG | GCAGGTTTGAGTTTGAGCTAG | | 100 | | 9.6 | 1.2 | | 0.24 | | AGAAAGAGCGAGTCTGTTGGAAATCAGTGCAGTGAGAGGATGATTCTCACGCTCTC[AT]TGCCAGGGTCTAGCTCAAACTCAAACCTGC | | amplifies SNP in *americana* | |  |  |
| **MC_39** | CTCCTAAGTGTAGGGTAAGAGC | ATTTCCATGTGGTGCTTTGG | | 100 | | 9.6 | 1.2 | | 0.24 | | CTCCTAAGTGTAGGGTAAGAGCTTCTGGTAC[AG]AACGAGGAGACGGAAGCTGGAGGAGAAGGTCCAATTCCAAAGCACCACATGGAAAT | |  | |  |  |
| **MC_40** | AGGTGGAAAGAAACCCAGTG | CAGGACAGGCAGTGAAGTTC | | 100 | | 9.6 | 1.2 | | 0.24 | | AGGTGGAAAGAAACCCAGTGGGAGTCCAGAAGATCAGCGTAAGGAGTGGTCTGTCAACTCTGCGCCCCGCAGC[GA]TGAGCTCTGAACTTCACTGCCTGTCCTG | |  | |  |  |
| **MC_41** | TGGTGCTGATTTCAACAACC | GTGCCAGTAGGTAGAGCAG | | 100 | | 14.0 | 1.75 | | 0.35 | | TGGTGCTGATTTCAACAACCAAAGAGTAATAGGGAGGTCATTCTGCGGGGCAC[GA]AAGAAATGAGGTTACCTCTGTGGGACCCCGCTGCTCTACCTACTGGCAC | |  | |  |  |
| **MC_42** | ACTCGAGAGCTTTGCAGAAG | GGTGGCAGCCTTGACCTC | | 100 | | 9.6 | 1.2 | | 0.24 | | ACTCGAGAGCTTTGCAGAAGCGCATGCAGCCGCTTGGTGCGAAAACACCAGCCAC[CT]CTCAGCAATT[CT]CGAGGTCAAGGCTGCCACC | | 4 haplotypes | |  |  |
| **MC_43** | CTTCTCCACCATACGCGATG | GGCAAACATAGCCCAGAATTC | | 100 | | 14.4 | 1.8 | | 0.36 | | CTTCTCCACCATACGCGATGCTGGGGTCGGCGTCTGTGTCCCGTCTGTTTGGTTGTGTTCTTCTGGTGGCTGGCTGCTCCGAC[AG]ATGGACTGACAGAATTCTGGGCTATGTTTGCC | |  | |  |  |
| **MC_44** | TGTCAGTTAGAGGAAGAGACC | GGATCCAAGAAAATGTGTAGGG | | 100 | | 16.8 | 2.1 | | 0.42 | | TGTCAGTTAGAGGAAGAGACCTACACCTTCTATACCGCA[AG]GCTAGGAGATTCAGAGTCTTAGTGATGCTGCTGTGTCTCCCCAACATC[GA]TCAGACCCTACACATTTTCTTGGATCC | | SNPs fully linked: AG, GA | |  |  |
| **MC_46** | AACTTCCCGCTTGTCCGAG | ACACTTGGAAGCTGTTGGTG | | 100 | | 7.2 | 0.9 | | 0.18 | | CTTTGTTCCCTGGTCCATCCCTGAGCAGGATCTGAGAGGTGATGGGCCAAGGTGACAAAGGGAG[CA]CTTTCGTGTGTGTCGTGC | |  | |  |  |
| **MC_56** | GTTTAGCTGACCCAGTAGGC | GAATCTGGGAGAGTGGTTGG | | 200 | | 4.0 | 1 | | 0.2 | | GTTTAGCTGACCCAGTAGGCTTC[CT]TCCCTTAGTTGACAAGAGCTCAGGACACTTCCTGGTGACTGCTGCTTTGGGAATTAACTTCCCCAACCACTCTCCCAGATTC | |  | |  |  |
| **MC_57** | GCAGGTTTCATTACTGATGGC | TGGCTTCCTCAGTGTACAAC | | 200 | | 4.0 | 1 | | 0.2 | | GCAGGTTTCATTACTGATGGCCAC[GA]CTCGAGGCCACGGCGGCCTGGGGATGGTGGTTAGTGTTGTACACTGAGGAAGCCA | |  | |  |  |
| **MC_58** | TGGAGCATGTTCCCCATTG | CCGGATTCTTCCCCAGCC | | 200 | | 2.9 | 0.72 | | 0.144 | | TGGAGCATGTTCCCCATTGCACG[GA]CAGACACACAGTGGC[GA]CAGGAGGGCACTGGCTGGGGAAGAATCCGG | | 4 haplotypes | |  |  |
| **MC_62** | GAGACCCCGATCCTTGGG | CCTTGCGATCCAGGATGAAG | | 200 | | 3.6 | 0.9 | | 0.18 | | GAGACCCCGATCCTTGGGCACGTGTG[TC]GTTCCTAGCATTTCCCCCGAGTCCTTCATCCTGGATCGCAAGG | |  | |  |  |
| **MC_63** | CAGTCTTGACACCATTGTGC | GTGGTACATGGACAGCTCAG | | 200 | | 6.0 | 1.5 | | 0.3 | | CAGTCTTGACACCATTGTGCCCATGAATTTCAGGACCTTGCCTGTATTGGGAATACTTACGAAGC[GA]CCCAGTCAATTCATAAATCCTGAGCTGTCCATGTACCAC | |  | |  |  |
| **MC_64** | CCAGTTTCCAAGGACGAGG | CGGAAGATGCCAGATCAGAC | | 200 | | 6.0 | 1.5 | | 0.3 | | CCAGTTTCCAAGGACGAGGATCAGGATGGCGGGAGCCCCCAGCGAAAGGCTCTGGGCCTGGGCC[AG]TGTCCCTGAATACCCAAACGTGTCTGATCTGGCATCTTCCG | |  | |  |  |
| **MC_65** | CAGCGTCCTGGAAGCCAG | TGCCGTGGTTTACCATCAG | | 200 | | 6.0 | 1.5 | | 0.3 | | CAGCGTCCTGGAAGCCAGCACTGCCGCCCGCATGGTGCCCC[AG]CCGACC[TC]GCTCCCGATGGTCCCACCTCACCTGGCCTGGCACCGGCCGAGGGACGTGTGCTGATGGTAAACCACGGCA | | SNPs fully linked: AT, GC; amplifies SNP in americana | |  |  |
| **MC_66** | GGTGCTAAGGTTCAGCTTTTC | AGTCACAATCCCCAGGGTAG | | 200 | | 6.0 | 1.5 | | 0.3 | | GGTGCTAAGGTTCAGCTTTTCAGAGC[GT]CGAAGAACAGCTGATTTCCATCCCTCCACAAAAACCCTTTACTACCCTGGGGATTGTGACT | |  | |  |  |
| **MC_68** | CTCACACTAGGACCAGAAGC | GCCTGTTACTTCCAATGACC | | 200 | | 4.0 | 1 | | 0.2 | | CTCACACTAGGACCAGAAGCCAG[CT]GCTCTTAAGACCAGTCTGAGGAAGAGGGTCATTGGAAGTAACAGGC | |  | |  |  |
| **MC_69** | TGACAGAAGCCATCTGCAC | GAAGCCTCCTCCCTCACC | | 200 | | 3.2 | 0.8 | | 0.16 | | TGACAGAAGCCATCTGCACAGAACCCGAGATCTCATGGAAACCGTACAGCAGCCA[TC]GGGGGTGACTGGTGAGGGAGGAGGCTTC | |  | |  |  |
| **MC_70** | GGCACTTAGAGCAAATCGTG | AAGTCTGTTGTGTACCTGCG | | 200 | | 4.0 | 1 | | 0.2 | | GGCACTTAGAGCAAATCGTGTGATGACTTGTCCATTTTC[GA]TCCGCATCTGTG[AG]TCTCAGGATCTGGGGGGGGTGCTTGCCCCACGCAGGTACACAACAGACTT | | 4 haplotypes; amplifies SNP in americana | |  |  |
| **MC_71** | CAGAGGTGGGGAGCAACC | TGTGGTAGATTCACAGACAGG | | 200 | | 4.8 | 1.2 | | 0.24 | | CAGAGGTGGGGAGCAACCCCTCAGGCCATGTCCCTCTCC[CT]GTCC[GA]CTGTGTCCCGTGCCCTGTGCAGGCCTGGCGCCTGGGAGGGGCTGGTGGGATCCTGTCTGTGAATCTACCACA | | SNPs fully linked | |  |  |
| **MC_73** | GGAAGTTGCTGGACAGAATG | GTACCTGCACAACAACAAGC | | 200 | | 6.0 | 1.5 | | 0.3 | | GGAAGTTGCTGGACAGAATGAG[GT]ATCTCCACGTTGCTGGAGCCGTTGAACATGTTGTCTGGCAGCCCGGCATCCGCCAGCTTGTTGTTGTGCAGGTAC | |  | |  |  |
| **MC_74** | CAGCAACAGAGGGTAGAGAG | GTCCTACAGAGCGGATTCC | | 200 | | 6.0 | 1.5 | | 0.3 | | CAGCAACAGAGGGTAGAGAGGACTTATTCTAACCCCACAAGCACCTGGGCATCAGCAGGAGCCCCTGTCCCCCCAAGAACTCTT[GC]CTGCGGAATCCGCTCTGTAGGAC | |  | |  |  |
| **MC_76** | TGTGTTTTCCACGCCCAC | TCCATTTTATAGACGGGAAACG | | 200 | | 4.0 | 1 | | 0.2 | | TGTGTTTTCCACGCCCACCACCCACACCAACGCAGAGGATAAGTGT[TC]CGTGAAGAAAGCCCTTCCCCCCACCCAGGGCCTCACGTTTCCCGTCTATAAAATGGA | |  | |  |  |
| **MC_77** | GGAGAGGTAAGTTCTGAGGG | ACCTTCCTGCATTTCTGGTG | | 200 | | 4.0 | 1 | | 0.2 | | GGAGAGGTAAGTTCTGAGGG[CT]TGAGGTAAGACAGGGTAAGACAGGACAAGTAAGACAGGGGCATAAAGCTGCTAGGAGGCACCAGAAATGCAGGAAGGT GGAGAGGTAAGTTCTGAGGGCTGAGGTAAGACAGGGTAAGACAGGGTAAGACAGGACAAGTAAGACAGGGGCATAAAGCTGCTAGGAGGCACCAGAAATGCAGGAAGGT | | 9 bp insertion in *americana* | |  |  |
| **MC_78** | TCTCCTCGATCTACTAGGTCC | AGGCTCATAGTTCCATGTGTC | | 200 | | 4.0 | 1 | | 0.2 | | TCTCCTCGATCTACTAGGTCCAGGCCAAGGCTGCCACCTGTAGGGACTGTCACTTTGTGC[GA]AAGTGACACATGGAACTATGAGCCT | |  | |  |  |
| **MC_83** | GACATTGTTACATTTGCATGGG | CACCCAGGGGTCTCAGAC | | 200 | | 8.0 | 2 | | 0.4 | | GACATTGTTACATTTGCATGGGATGCAGGTGGCCGAGTGCCAAGGGTGTCTCTCCAAAC[CT]GAACTGAGGAGTGTCCATGATGTGTCCTCA[CT]GCAGGGTCTGAGACCCCTGGGTG | | 4 haplotypes | |  |  |
| **MC_84** | CTAAATCCTGGCTCCCCATG | GATCTACATCCTTTTACATCCCC | | 200 | | 3.2 | 0.8 | | 0.16 | | CTAAATCCTGGCTCCCCATGTG[CT]TGAGGGTCTTGGAGAATGCTTGAGTAAGACATTTCCTTGTGGGGGGATGTAAAAGGATGTAGATC | |  | |  |  |
| **MC_85** | CTTTCCTTCCTGTCAGTCGG | AGGCAATTCGAAACTTTCAGAG | | 200 | | 4.0 | 1 | | 0.2 | | CTTTCCTTCCTGTCAGTCGGCTCAGC[CT]TTTCTAGGGATCTCCTGACCTTTCCTCTGAAAGTTTCGAATTGCCT | |  | |  |  |
| **MC_86** | CATGTGGGAACATGACCATG | TCCATAGAGGAACCTCCTGG | | 200 | | 4.8 | 1.2 | | 0.24 | | CATGTGGGAACATGACCATGAATGCAATAGGCTTTGA[CT]GACGAACACTGCCGTTCTGTAACCAGGAGGTTCCTCTATGGA | |  | |  |  |
| **MC_87** | CGAGGTCTACAGCACCAAG | CACGTCAAAGGACACCTCC | | 200 | | 6.0 | 1.5 | | 0.3 | | CGAGGTCTACAGCACCAAGAT[CG]AGCTGCAAGGT[GC]ACCTCCCGATTTGCTCACAACGTCGTCACCACCAGAGCTGTCAACCGTGCAGACACGGCCAAGGAGGTGTCCTTTGACGTG | | 4 haplotypes | |  |  |
| **MC_90** | CACCACCATGATACCTTGGG | CAGAGGACTGAGCTTAAGCC | | 200 | | 6.0 | 1.5 | | 0.3 | | CACCACCATGATACCTTGGGCCAGCCATGTATCTGGTGTCATCTCCTGAAAATTCC[GA]TGGCCTGGGTGTCTGGGTGTCCTCTGTTGCATGGGCTTAAGCTCAGTCCTCTG | |  | |  |  |
| **MC_92** | AAGTTGCAGCCTGGGAATG | GCCTTGTAATTTGCCCTTGG | | 200 | | 2.9 | 0.72 | | 0.144 | | AAGTTGCAGCCTGGGAATGGAGAGGG[CA]AGGGCACACTGGGCAGGCAGGAGGACTGTGACCCAAGGGCAAATTACAAGGC | |  | |  |  |
| **MC_94** | CAGAGAGTGAGTTTTGGAACC | CTTTGTAGTTCCCAAGACAGAC | | 200 | | 6.0 | 1.5 | | 0.3 | | CAGAGAGTGAGTTTTGGAACCAATTACCCATTTTT[TC]CCCACCAGGGCAGGGAGTCTGTCTTGGGAACTACAAAG | |  | |  |  |
| **MC_97** | GATTCCAGGTGTTCGGAAAAC | GAACTGACACTCAACTGGTTC | | 200 | | 4.8 | 1.2 | | 0.24 | | GATTCCAGGTGTTCGGAAAACGTCTTCTTGCCCACTTCGTAGC[GC]AGTGCCCAGAACCAGTTGAGTGTCA | |  | |  |  |
| **MC_98** | CAGTGCAGTATGGTCCTCC | AAGTTTGAACACAGGGCCAG | | 200 | | 3.6 | 0.9 | | 0.18 | | CAGTGCAGTATGGTCCTCCGTCTCCGTTACCCTGGTGC[TC]GAAGTCGCCGCGTCGCCGCCGCCAGGGAACTACGGTCCCCGCCCCTAACCTGGCCCTGTGTTCAAACTT | |  | |  |  |
| **MC_99** | TTTCTGTGCGGATAAACGGG | CTGGCATTAGCTCCCTGTG | | 200 | | 4.0 | 1 | | 0.2 | | TTTCTGTGCGGATAAACGGGGTTATGGAAG[AG]TTCTAGAAGGATACCTGCCAGCCAGGGAGAGGGGAGGGCACAGGGAGCTAATGCCAG | |  | |  |  |
| **MC_102** | CAAGGATGAGCAGCATTAGC | GGAGATGCTGAGTTTGAGGG | | 200 | | 4.0 | 1 | | 0.2 | | CAAGGATGAGCAGCATTAGCCTGCGAAGGCGTCAG[CG]CCTCCCTACCGCAAAGGCACCGCCCAGAGCCCTCAAACTCAGCATCTCC | |  | |  |  |
| **MC_104** | AGCACCTCCCCAAGTGTG | CCTACAGGAGCGAAAAGACG | | 200 | | 4.0 | 1 | | 0.2 | | AGCACCTCCCCAAGTGTGGGGCACAGCCCATATCACCCTACGGGATCCCCAACACCT[GA]CGTCTTTTCGCTCCTGTAGG | |  | |  |  |
| **MC_105** | GTCATGCTTATGACATTGCCC | GCAAGCAAAGGTCTTCCTAC | | 200 | | 4.0 | 1 | | 0.2 | | GTCATGCTTATGACATTGCCCGATGTGTG[CT]CAGCTCTTTAAATGTTACGATGTGCAGCTATACAAGGTAGGAAGACCTTTGCTTGC | |  | |  |  |
| **MC_106** | TCAGTGTTCCCAGTAGAATCC | AGTTTCTGAGGTGAGAGAAGG | | 200 | | 6.0 | 1.5 | | 0.3 | | TCAGTGTTCCCAGTAGAATCCTCA[TC]CAAACACATGGTGAAAGACAATGGCAAGATACCTTCTCTCACCTCAGAAACT | |  | |  |  |
| **MC_108** | CCCTTCGCAATCCTCTCAAG | ATAAGGCAGCTCAAACAGGG | | 200 | | 3.2 | 0.81 | | 0.162 | | CCCTTCGCAATCCTCTCAAGACGTTCCATGAGTGGAGGAGGCTGG[TG]CTCGACCCTGTTTGAGCTGCCTTAT | |  | |  |  |
| **MC_109** | TTTAGCAGGCAAGGTGACC | CATTCCCCTAAACGCTCCC | | 200 | | 4.0 | 1 | | 0.2 | | TTTAGCAGGCAAGGTGACCGAGAGAGTCATTTTTT[TA]TAAATTAGGTCTAGGACAGCGCTTACCTCCTGGGAGCGTTTAGGGGAATG | |  | |  |  |
| **MC_111** | TGCAGACCCAGTACAAGC | GAAATCGTGCGCCTCCAG | | 200 | | 4.0 | 1 | | 0.2 | | TGCAGACCCAGTACAAGCAGCTTGTGGGCGACCTGCTAGGCTGGATT[GT]CGGAGAAGCAGGCGCAGCTGGAGGCGCACGATTTC | |  | |  |  |
| **MC_113** | GGAATATCGTGGCCATGAAAG | TCTTTGTCACGTGTGTTTAGTTC | | 200 | | 6.0 | 1.5 | | 0.3 | | GGAATATCGTGGCCATGAAAGACACCAAGAAGCAGGGAGCTCGTCC[AT]GATGAACTAAACACACGTGACAAAGA | |  | |  |  |
| **MC_116** | CATAGACTTTCCTGGACGGC | TAGTGGTTCTAGCTTTGGCG | | 200 | | 4.0 | 1 | | 0.2 | | CATAGACTTTCCTGGACGGCCTGACGCCTGAGTGTCTCCCACTGGATGCACACGT[GA]TCTTGGGCTCAGAACCGCCAAAGCTAGAACCACTA | |  | |  |  |
| **MC_118** | GCAGGAGAGCAGAGAGAATC | GCTTCTCATTTTGTGACGGC | | 200 | | 4.8 | 1.2 | | 0.24 | | GCAGGAGAGCAGAGAGAATCAGGTGACTGGACTGAGAA[GA]CCTAATTTTATTGCCGTCACAAAATGAGAAGC | |  | |  |  |
| **MC_119** | GTTCATGTGCATTTAACCGC | CGTGTGACTGTTTTGTTCTTG | | 200 | | 8.0 | 2 | | 0.4 | | GTTCATGTGCATTTAACCGCAAGCTGTCCGACGCCCAGCGGGCACTTAGTCCACACCTGTCCA[CT]GTGTCTGATGTCAACCAAGAACAAAACAGTCACACG | |  | |  |  |
| **MC_120** | GGACCTGAGCAACTATCACC | GGATGACATGCACCTTGTTC | | 200 | | 6.0 | 1.5 | | 0.3 | | GGACCTGAGCAACTATCACCTGATGGACCTGGGCCACCCCCACCACTCCATTCGCTGCATGGCCGTCGTGTGTGACCGCGTGTGGTGTGGCTACA[AG]GAACAAGGTGCATGTCATCC | |  | |  |  |
| **MC_121** | CAGGAAACCCTCACCGAG | GCTAGAGCTGACCGATCAC | | 200 | | 4.0 | 1 | | 0.2 | | CAGGAAACCCTCACCGAGGCCCTGGCAGTGGCCACAGGCTTGGATCAAG[GT]AGTGTCCACAGGAATAGGAAGGAGGGAACATTTAGGCCTTGGTGATCGGTCAGCTCTAGC | |  | |  |  |
| **MC_122** | AGGGCCGAGAAGACATTATATG | AGTCGATTAGTTCACGCGTG | | 200 | | 4.0 | 1 | | 0.2 | | AGGGCCGAGAAGACATTATATGATCACTAACCAGGCACGAACCCTACCTATCAGGTAGCTGAGC[AG]CACGCGTGAACTAATCGACT | |  | |  |  |
| **MC_124** | AGAGGATCCTGGGCTTGAG | ACGGTGGTCCTCATTTCAC | | 200 | | 4.0 | 1 | | 0.2 | | AGAGGATCCTGGGCTTGAGGC[TG]GCCGATTCAGAGCGTGGGGATAGCAATGTCGACTGTGCTGAAGAGCGAGGAGGAGCGCAAAGTGAATTGTGTGAAATGAGGACCACCGT | |  | |  |  |
| **MC_125** | TAACACACGACCCCTTTTCC | GCCAAACCCAGCACTTTATC | | 200 | | 4.0 | 1 | | 0.2 | | TAACACACGACCCCTTTTCC[GA]TTAGAATTCAGCCAGAGCAGAGCCAGACGTGACCCGGTCACCCCTTCCTGGCCAGAGATAAAGTGCTGGGTTTGGC | | amplifies SNP in *americana* | |  |  |
| **MC_127** | TCCACCCGCTACAGACTC | TTTTGGACCCGAGATGTGC | | 200 | | 3.6 | 0.9 | | 0.18 | | TCCACCCGCTACAGACTCGGGCGCGAG[CG]GCGTTTAGCTCCCCACAAAACGCATCGGCTCCTCAGGCGTCCCCAAGCCTGGGCAGGGCTGCACATCTCGGGTCCAAAA | |  | |  |  |
| **MC_128** | GACTGAAACAGTGTATGTCCC | GCCCCTAATCTGTGGCAG | | 200 | | 3.6 | 0.9 | | 0.18 | | GACTGAAACAGTGTATGTCCCTCCACACACTGACACTGGGACTT[CT]TGGCTCCATACTGTAATTTGAACGGTCCTGCCACAGATTAGGGGC | |  | |  |  |
| **MC_132** | GAGCCACATTGAGAAGAACATC | CAAGGTCAGGAAGCGGTAG | | 200 | | 6.0 | 1.5 | | 0.3 | | GAGCCACATTGAGAAGAACATCAGGGATC[AG]GAGTAGGGAGGGAGCCAACCTGGAGTACCTCAAAAACATCATCTACCGCTTCCTGACCTTG | |  | |  |  |
| **MC_134** | AGATCACTTTCCGCGACTG | TGTCATTCCCAGATCCAAGG | | 200 | | 4.0 | 1 | | 0.2 | | AGATCACTTTCCGCGACTGCACACGTCTGTCTGCAATGGTTTGAAATGCTAGG[GC]GTGGGCCTTGGATCTGGGAATGACA | |  | |  |  |
| **MC_135** | CAGAGGAAGAACATTTCCGTG | GGGGCAGGCATTAGAGGG | | 200 | | 4.8 | 1.2 | | 0.24 | | CAGAGGAAGAACATTTCCGTGAAAGTCTGTTGC[GT]AAGTGCTAGCCTGGAAGGTGGGGACGGGTGCGGGGCTCCCTCTAATGCCTGCCCC | |  | |  |  |
| **MC_138** | GGTTAGAAATAATTCCCAGGCC | TCAGTGTCAACTTGTTTAGGTC | | 200 | | 6.0 | 1.5 | | 0.3 | | GGTTAGAAATAATTCCCAGGCCAGCTGTTTGGACATCACTCTTGGTTCTTGT[GA]TCCATGTATTAGCC[GA]TGTAGACCTAAACAAGTTGACACTGA | | SNPs fully linked | |  |  |
| **MC_140** | TCAAAGACCGAGAAGACACC | GTGGATTAGCTTTGTTCCCG | | 200 | | 4.0 | 1 | | 0.2 | | TCAAAGACCGAGAAGACACCTGTCCTGAGAAGTAAACGGAAAGCTCTTTGAGAAGCCAAGGCAAGAGTGTGCTGGGCAGGGGCAGAAGGTCCG[AC]GGCGGGAACAAAGCTAATCCAC | |  |  |  |  |
| **MC_146** | AAGGCCATGACATCATAGGC | CATAGCGGGTGTTGACAAAG | | 200 | | 6.0 | 1.5 | | 0.3 | | AAGGCCATGACATCATAGGCCCTGACACTGGAGATGGCAGCC[TC]CAGAAGGTATGACTGTCAAAAAGAGATGTCACAGCTTTGTCAACACCCGCTATG | |  | |  |  |
| **MC_148** | CAGGTGACCACCGTGGAC | GTGTAGGTGACATTGGCGAG | | 200 | | 3.2 | 0.8 | | 0.16 | | CAGGTGACCACCGTGGACCTGCGGGATGAGAG[CT]GTGGCCCGCGGCCGCATAGACAATGTCGATGCTTTCATGAACATCCGCCTCGCCAATGTCACCTACAC | |  | |  |  |
| **MC_149** | TTCATCCCACTGGTCATCAC | CCGACATTCCCCGTCATAC | | 200 | | 4.0 | 1 | | 0.2 | | TTCATCCCACTGGTCATCACAGACAGTCCCCCAATGACCAGCATGGTAGATTTCCACTCGGCCTTCCCGCGCGCTGCTCCCACC[AC]ACGAGGCGTATGACGGGGAATGTCGG | |  | |  |  |
| **MC_151** | GTGAATCAGGAAGCCATTTCC | ACAGAAAAGTCCTTCTCCAGG | | 200 | | 4.0 | 1 | | 0.2 | | GTGAATCAGGAAGCCATTTCCAACG[CT]GCAGAGGATCCATAAGTGGGCCATCCTGGAGAAGGACTTTTCTGT | |  | |  |  |
| **SRY_MAAM_1** | GTGATCAAAGGCGCAAGGTG | CCTGTATTCTCTGCGCCTCC | | 200 | | 6.0 | 1.5 | | 0.3 | | GTGATCAAAGGCGCAAGGTGGCTCTAGAGAATCCCCGAATGCAAAACTCAGAGATCAGCAAGCAGCTGGGGTACCAGTGGAAAATGCTTACAGAAGCCGAAAAACGGCCATTCTTCGAGGAGGCGCAGAGAATACAGG | | same as *americana* | |  |  |
| **SRY_MAAM_4** | TCAGAGATCAGCAAGCAGCT | TTGCGCCTCCTCGAAGAATG | | 200 | | 4.0 | 1 | | 0.2 | | TCAGAGATCAGCAAGCAGCTGGGGTACCAGTGGAAAATGCTTACAGAAGCCGAAAAACGGCCATTCTTCGAGGAGGCGCAA | |  | |  |  |
|  |  |  | **H2O added:** | | **158 µl** | | |  | |  | |  | | | |  |
|  |  |  | **Total volume:** | | **800 µl** | | | - | |  | |  | | | |  |

###### **Table S7.** Bootstrap confidence intervals calculated by taking 999 bootstraps of GBAS dataset across SNPs. Humboldt marten populations in bold. Note more precise estimates with larger sample sizes. **Humboldt marten regions in bold.**

|  | N | **N. Dunes** | **S. Dunes** | **S. Coast** | **S. Oregon** | **Applegate** | **Border** | **N. California** | **Maple Crk** | OR Cascades | Lassen | WA Cascades | Blue Mtns | Wyoming | Colorado |
| --- | --- | --- | --- | --- | --- | --- | --- | --- | --- | --- | --- | --- | --- | --- | --- |
| **N.Coast** | 4 | 0.23−0.38 | 0.20−0.33 | 0.05−0.26 | 0.15−0.27 | 0.18−0.41 | 0.19−0.34 | 0.14−0.27 | 0.23−0.39 | 0.25−0.40 | 0.29−0.45 | 0.33−0.53 | 0.37−0.54 | 0.32−0.49 | 0.42−0.59 |
| **N.Dunes** | 14 | - | 0.36−0.48 | 0.26−0.48 | 0.27−0.40 | 0.30−0.51 | 0.35−0.48 | 0.29−0.42 | 0.33−0.47 | 0.30−0.46 | 0.39−0.55 | 0.43−0.61 | 0.45−0.60 | 0.40−0.56 | 0.48−0.62 |
| **S.Dunes** | 37 | 0.36−0.48 | - | 0.08−0.21 | 0.11−0.20 | 0.04−0.24 | 0.11−0.23 | 0.10−0.18 | 0.17−0.29 | 0.18−0.30 | 0.22−0.36 | 0.31−0.46 | 0.32−0.46 | 0.26−0.43 | 0.31−0.47 |
| **S.Coast** | 2 | 0.26−0.48 | 0.08−0.21 | - | 0.00−0.10 | -0.36−-0.01 | 0.01−0.12 | 0.00−0.10 | 0.06−0.26 | 0.09−0.25 | 0.17−0.35 | 0.03−0.35 | 0.26−0.47 | 0.04−0.28 | 0.30−0.51 |
| **S.Oregon** | 19 | 0.27−0.40 | 0.11−0.20 | 0.00−0.10 | - | -0.01−0.07 | 0.04−0.11 | 0.03−0.07 | 0.05−0.14 | 0.15−0.25 | 0.18−0.31 | 0.26−0.43 | 0.29−0.44 | 0.22−0.38 | 0.30−0.44 |
| **Applegate** | 1 | 0.30−0.51 | 0.04−0.24 | -0.36−-0.01 | 0.01−0.07 | - | 0.05−0.18 | 0.02−0.14 | 0.09−0.28 | 0.12−0.32 | 0.16−0.37 | 0.18−0.49 | 0.32−0.55 | 0.20−0.47 | 0.40−0.63 |
| **Border** | 7 | 0.35−0.48 | 0.11−0.23 | 0.01−0.12 | 0.04−0.11 | 0.05−0.18 | - | 0.00−0.06 | 0.07−0.17 | 0.15−0.27 | 0.19−0.32 | 0.23−0.40 | 0.27−0.44 | 0.22−0.37 | 0.29−0.43 |
| **N.California** | 19 | 0.29−0.42 | 0.10−0.18 | 0.00−0.10 | 0.03−0.07 | 0.02−0.14 | 0−0.06 | - | 0.02−0.09 | 0.14−0.24 | 0.17−0.30 | 0.23−0.37 | 0.27−0.41 | 0.20−0.34 | 0.27−0.41 |
| **MapleCrk** | 5 | 0.33−0.47 | 0.17−0.29 | 0.06−0.26 | 0.05−0.14 | 0.09−0.28 | 0.07−0.17 | 0.02−0.09 | - | 0.14−0.25 | 0.17−0.32 | 0.23−0.40 | 0.28−0.43 | 0.19−0.35 | 0.32−0.48 |
| OR.Casc | 9 | 0.30−0.46 | 0.18−0.30 | 0.09−0.25 | 0.15−0.25 | 0.12−0.32 | 0.15−0.27 | 0.14−0.24 | 0.14−0.25 | - | 0.11−0.22 | 0.07−0.23 | 0.14−0.27 | 0.12−0.26 | 0.22−0.35 |
| Lassen | 11 | 0.39−0.55 | 0.22−0.36 | 0.17−0.35 | 0.18−0.31 | 0.16−0.37 | 0.19−0.32 | 0.17−0.30 | 0.17−0.32 | 0.11−0.22 | - | 0.16−0.29 | 0.20−0.33 | 0.19−0.36 | 0.25−0.41 |
| WA.Casc | 2 | 0.43−0.61 | 0.31−0.46 | 0.03−0.35 | 0.26−0.43 | 0.18−0.49 | 0.23−0.40 | 0.23−0.37 | 0.23−0.40 | 0.07−0.23 | 0.16−0.29 | - | 0.09−0.27 | -0.03−0.24 | 0.18−0.41 |
| Blue Mtns | 10 | 0.45−0.60 | 0.32−0.46 | 0.26−0.47 | 0.29−0.44 | 0.32−0.55 | 0.27−0.44 | 0.27−0.41 | 0.28−0.43 | 0.14−0.27 | 0.20−0.33 | 0.09−0.27 | - | 0.14−0.33 | 0.17−0.35 |
| Wyoming | 2 | 0.40−0.56 | 0.26−0.43 | 0.04−0.28 | 0.22−0.38 | 0.20−0.47 | 0.22−0.37 | 0.20−0.34 | 0.19−0.35 | 0.12−0.26 | 0.19−0.36 | -0.03−0.24 | 0.14−0.33 | - | 0.08−0.29 |
| Colorado | 5 | 0.48−0.62 | 0.31−0.47 | 0.30−0.51 | 0.30−0.44 | 0.40−0.63 | 0.29−0.43 | 0.27−0.41 | 0.32−0.48 | 0.22−0.35 | 0.25−0.41 | 0.18−0.41 | 0.17−0.35 | 0.08−0.29 | - |

###### **Table S8.** Mitogenome nucleotide diversity estimate details for other mammalian carnivores.

| Common Name | Species | IUCN Status | Estimate | Variance | N | Source |
| --- | --- | --- | --- | --- | --- | --- |
| Striped hyena | *Hyaena hyaena* | VU | 0.00023 | - | 14 | Westbury et al. 2018 |
| Island gray fox (Santa Cruz) | *Urocyon littoralis* | NT | 0.00024 | - | 42 | Hofman et al. 2015 |
| European mink (France-Spain) | *Mustela lutreola* | CR | 0.00024 | 9.00 x  (10^-10) | 42 | Hofman et al. 2015 |
| Black-footed ferret | *Mustela nigripes* | EN | 0.000299 | - | 4 | Etherington et al. 2022 |
| Iberian lynx | *Lynx pardinus* | VU | 0.00034 | 1.02 x (10^-7) | 59 | Skorupski et al. 2024 |
| Malayan Tiger | *Panthera tigris jacksoni* | EN | 0.0004095 | - | 9 | Liu et al. 2018 |
| Mediterranean monk seal | *Monachus monachus* | EN | 0.0005 | - | 14 | Rey-Iglesia et al. 2021 |
| Cheetah (Namibia) | *Acinonyx jubatus* | VU | 0.00071 | 1.09 x (10^-7) | 4 | Dobrynin et al. 2015 |
| Iberian Wolf | *Canis lupus signatus* | NT | 0.00082 | 1.85 x (10^-7) | 29 | Salado et al. 2023 |
| Fisher | *Pekania pennanti* | LC | 0.00088 | - | 40 | Knaus et al. 2011 |
| African wild dogs | *Lycaon pictus* | EN | 0.0009 | - | 20 | Tensen et al. 2025 |
| Himalyan red panda | *Ailurus styani* | EN | 0.001005 | - | 13 | Hu et al. 2020 |
| Eurasian otter (UK) | *Lutra lutra* | NT | 0.0016 | - | 46 | Du Plessis et al. 2025 |
| Snow leopard | *Panthera uncia* | VU | 0.00177 | 6.40 x (10^-9) | 136 | Wang et al. 2025 |
| Chinese red panda | *Ailurus fulgens* | EN | 0.0037 | - | 36 | Hu et al. 2020 |
| Flat-headed cat | *Prionailurus planiceps* | EN | 0.00396 | 2.30 x (10^-7) | 21 | Patel et al. 2017 |
| Siberian weasel | *Mustela siberica* | LC | 0.00519 | - | 20 | Shalabi 2016 |
| Sable | *Martes zibellina* | LC | 0.0058 | 1.00 x (10^-8) | 75 | Le et al. 2021 |
| Japanese weasel | *Mustela itatsi* | NT | 0.00619 | - | 26 | Shalabi 2016 |

**LITERATURE CITED**

Andrews, S. (2010). FastQC: a quality control tool for high throughput sequence data [Online]. Available online at: <http://www.bioinformatics.babraham.ac.uk/projects/fastqc/>

Bresadola, L., Link, V., Buerkle, C.A., Lexer, C., Wegmann, D. (2020). Estimating and accounting for genotyping errors in RAD-seq experiments. *Molecular Ecology Resources*, 20(4), 856-870. <https://doi.org/10.1111/1755-0998.13153>

Campbell, G.M. , Pauli, J.N. , Thomas, J.G., McClean, T. (2010). Accuracy in molecular sexing of martens (*Martes americana* and *Martes caurina*) varies among sample types. *Molecular Ecology Resources*, 10(6), 1019-1022. <https://doi.org/10.1111/j.1755-0998.2010.02856.x>

Catchen, J., Hohenlohe, P.A., Bassham, S., Amores, A., Cresko, W.A. (2013). Stacks: an analysis tool set for population genomics. Molecular Ecology, 22(11), 3124-3140. <https://doi.org/10.1111/mec.12354>

Danecek P., Auton, A., Abecasis, G., Albers, C.A., Banks, E., DePristo, M.A., Handsaker, R.E., Lunter, G., Marth, G.T., Sherry, S.T., McVean, G., Durbin, R. (2011). The variant call format and VCFtools. *Bioinformatics*, 27(15), 2156-8. <https://doi.org/10.1093/bioinformatics/btr330>

Danecek, P., Bonfield, J.K., Liddle, J., Marshall, J., Ohan, V., Pollard, M.O., Whitwham, A., Keane, T., McCarthy, S.A., Davies, R.M., Li, H. (2021). Twelve years of SAMtools and BCFtools. *GigaScience*, 10(2), giab008, <https://doi.org/10.1093/gigascience/giab008>

Goudet, J. (2005). HIERFSTAT: a package for R to compute and test hierarchical F-statistics. *Molecular Ecology Notes*, 5(1), 184-186. <https://doi.org/10.1111/j.1471-8286.2004.00828.x>

Hall, E.R. (1981). The mammals of North America. 2d ed. New York: John Wiley & Sons. 1181 p.

Lucid, M., Cushman, S., Robinson, L., Kortello, A., Hausleitner, D., Mowat, G., Ehlers, S., Gillespie, S., Svancara, L.K., Sullivan, J., Rankin, A., Paetkau, D. (2020). Carnivore Contact: A Species Fracture Zone Delineated Amongst Genetically Structured North American Marten Populations (Martes americana and Martes caurina). *Frontiers in Genetics*, 11, 735. <https://doi.org/10.3389/fgene.2020.00735>

Mastretta‐Yanes, A., Arrigo, N., Alvarez, N., Jorgensen, T.H., Piñero, D., Emerson, B.C. (2014). Restriction site‐associated DNA sequencing, genotyping error estimation and de novo assembly optimization for population genetic inference. Molecular Ecology Resources, 15(1), 28–41. <https://doi.org/10.1111/1755-0998.12291>

McCartney‐Melstad, E., Gidiş, M., Shaffer, H. B. (2019). An empirical pipeline for choosing the optimal clustering threshold in RADseq studies. Molecular Ecology Resources, 19(5), 1195–1204. <https://doi.org/10.1111/1755-0998.13029>

McKinney, G.J., Waples, R.K., Seeb, L.W., Seeb, J. E. (2016). Paralogs are revealed by proportion of heterozygotes and deviations in read ratios in genotyping‐by‐sequencing data from natural populations. *Molecular Ecology Resources*, *17*(4), 656–669. <https://doi.org/10.1111/1755-0998.12613>

Mullins, J., Statham, M.J., Roche, T., Turner, P.D., O’Reilly, C. (2009). Remotely plucked hair genotyping: a reliable and non-invasive method for censusing pine marten (Martes martes, L. 1758) populations. European Journal of Wildlife Research, 56(3), 443–453. <https://doi.org/10.1007/s10344-009-0332-x>

O’Leary, S. J., Puritz, J. B., Willis, S. C., Hollenbeck, C. M., Portnoy, D. S. (2018). These aren’t the loci you’e looking for: Principles of effective SNP filtering for molecular ecologists. Molecular Ecology, 27(16), 3193–3206. https://doi.org/10.1111/mec.14792

Roffler, G. H., J. M. Allen, A. Massey, and T. Levi. (2021). Metabarcoding of fecal DNA shows dietary diversification in wolves substitutes for ungulates in an island archipelago. *Ecosphere*, 12(1), e03297. <https://doi.org/10.1002/ecs2.3297>

Paris, J. R., Stevens, J. R., Catchen, J. M. (2017). Lost in parameter space: a road map for stacks. Methods in Ecology and Evolution, 8(10), 1360–1373. <https://doi.org/10.1111/2041-210x.12775>

Pearman, W. S., Urban, L., Alexander, A. (2022). Commonly used Hardy–Weinberg equilibrium filtering schemes impact population structure inferences using RADseq data. *Molecular Ecology Resources*, *22*(7), 2599–2613. <https://doi.org/10.1111/1755-0998.13646>

Peterson, B.K., Weber, J.N., Kay, E.H., Fisher, H.S., Hoekstra, H.E. (2012). Double Digest RADSEQ: an inexpensive method for de novo SNP discovery and genotyping in Model and Non-Model species. *PLoS ONE*, *7*(5), e37135. <https://doi.org/10.1371/journal.pone.0037135>

Poland, J.A., Brown, P., Sorrells, M.E., Jannink, J.L. (2012). Development of high-density genetic maps for barley and wheat using a novel two-enzyme genotyping-by-sequencing approach. *PLOS One*, 7(2), e32253. <https://doi.org/10.1371/journal.pone.0032253>

Rochette, N.C., Catchen, J.M. (2017). Deriving genotypes from RAD-seq short-read data using Stacks. Nature Protocols, 12(12), 2640-2659. <https://doi.org/10.1038/nprot.2017.123>

Schwartz, M.K., Walters, A.D., Pilgrim, K.L., Moriarty, K.M., Slauson, K.M., Zielinski, W.J., Aubry, K.B., Sacks, B.N., Zarn, K.E., Quinn, C.B., Young, M.K. (2020). Pliocene–Early Pleistocene Geological Events Structure Pacific Martens (Martes caurina). *Journal of Heredity*, *111*(2), 169–181. <https://doi.org/10.1093/jhered/esaa005>

Shen, W., Sipos, B., Zhao, L. (2024). SeqKit2: A swiss army knife for sequence and alignment processing. *iMeta*, 3(3), e191. <https://doi.org/10.1002/imt2.191>

Untergasser, A., Cutcutache, I., Koressaar, T., Ye, J., Faircloth, B.C., Remm, M., Rozen, S.G. (2012). Primer3—new capabilities and interfaces. Nucleic Acids Research, 40(15), e115. <https://doi.org/10.1093/nar/gks596>

Weir, B.S., Goudet, J. (2017). A unified characterization of population structure and relatedness. Genetics, 206(4), 2085-2103. <https://doi.org/10.1534/genetics.116.198424>

Ye, J., Coulouris, G., Zaretskaya, I., Cutcutache, I., Rozen, S., Madden, T.L. (2012). Primer-BLAST: A tool to design target-specific primers for polymerase chain reaction. BMC Bioinformatics, 13(1), 134. <https://doi.org/10.1186/1471-2105-13-134>
